# Diagnostic yield of cancer predisposition in a nationwide prospective childhood acute leukemia cohort

**DOI:** 10.1038/s41467-026-78170-z

**Published:** 2026-09-28

**Authors:** Fulya Taylan, Anna Staffas, Sara Sjögren, Kristina Lagerstedt-Robinson, Ekaterina Kuchinskaya, Anna Poluha, Sofie Ivarsson, Maria Hellberg, Joakim Klar, Frida Abel, Jonas Abrahamsson, Cornelis Jan Pronk, Karin Belander Strålin, Arja Harila, Hartmut Vogt, Ulrika Norén-Nyström, Pernilla Grillner, Lars Palmqvist, Linda Arvidsson, Tatjana Pandzic, Marie-Louise Bondeson, Ninni Mu, Katja Ekholm, Britt-Marie Anderlid, Irina Golovleva, Carolina Maya-González, Pär Gideonsson, Emma Tham, Gisela Barbany, Päivi Lähteenmäki, Johanna Sandgren, Richard Rosenquist, Valtteri Wirta, Gustaf Ljungman, David Gisselsson, Josefine Palle, Bianca Tesi, Ann Nordgren

**Affiliations:** 1 Department of Molecular Medicine and Surgery, Karolinska Institutet, Stockholm, Sweden; 2 Department of Clinical Genetics and Genomics, Karolinska University Hospital, Stockholm, Sweden; 3 Department of Clinical Genetics and Genomics, Sahlgrenska University Hospital, Gothenburg, Sweden; 4 Department of Laboratory Medicine, Institute of Biomedicine, Sahlgrenska Academy, University of Gothenburg, Gothenburg, Sweden; 5 Department of Clinical Genetics, Linköping University Hospital, Linköping, Sweden; 6 Department of Clinical Genetics, Uppsala University Hospital, Uppsala, Sweden; 7 Department of Immunology, Genetics and Pathology, Uppsala University, Uppsala, Sweden; 8 Department of Clinical Genetics, Pathology and Molecular Diagnostics, Skåne University Hospital, Lund, Sweden; 9 Division of Clinical Genetics, Department of Laboratory Medicine, Lund University, Lund, Sweden; 10 Clinical Genomics Uppsala, Science for Life Laboratory, Uppsala University, Uppsala, Sweden; 11 Institution for Clinical Sciences, Department of Pediatrics, Sahlgrenska Academy, University of Gothenburg, Gothenburg, Sweden; 12 Childhood Cancer Center, Skåne University Hospital, Lund, Sweden; 13 Division of Molecular Hematology, Wallenberg Center for Molecular Medicine, Lund University, Lund, Sweden; 14 Department of Pediatric Hematology and Oncology, Karolinska University Hospital, Stockholm, Sweden; 15 Department of Medicine Huddinge, Center for Hematology and Regenerative Medicine, Karolinska Institutet, Stockholm, Sweden; 16 Department of Pediatric Hematology and Oncology, Uppsala University, Children’s Hospital, Uppsala, Sweden; 17 Department of Women’s and Children’s Health, Uppsala University, Uppsala, Sweden; 18 Department of Biomedical and Clinical Sciences, Linköping University, Linköping, Sweden; 19 Crown Princess Victoria Children´s Hospital, Region Östergötland, Linköping, Sweden; 20 Department of Clinical Sciences, Pediatrics, Umeå University, Umeå, Sweden; 21 Childhood Cancer Research Unit, Department of Women’s and Children’s Health, Karolinska Institutet, Stockholm, Sweden; 22 Department of Clinical Chemistry, Sahlgrenska University Hospital, Region Västra Götaland, Gothenburg, Sweden; 23 Department of Medical Biosciences, Medical and Clinical Genetics, Umeå University, Umeå, Sweden; 24 Department of Oncology-Pathology, Karolinska Institutet, Stockholm, Sweden; 25 Clinical Pathology and Cancer Diagnostics, Karolinska University Hospital, Stockholm, Sweden; 26 Genomic Medicine Center Karolinska, Karolinska University Hospital, Stockholm, Sweden; 27 Science for Life Laboratory, Department of Microbiology, Tumour and Cell Biology, Karolinska Institutet, Stockholm, Sweden; 28 Science for Life Laboratory, School of Engineering Sciences in Chemistry, Biotechnology and Health, KTH Royal Institutet of Technology, Stockholm, Sweden

**Keywords:** Paediatric cancer, Leukaemia, Cancer genetics

## Abstract

The utility of whole-genome sequencing (WGS) for detecting childhood leukemia predisposition remains unclear. We perform a nationwide, prospective, population-based study of 181 children with acute leukemia to assess diagnostic yield and clinical utility of a uniformly applied three-pronged strategy, including systematic phenotyping, germline WGS-based 189-gene panel and tumor sequencing of positive cases. Trio-WGS is performed in 11 high-suspicion families. Nine patients have pathogenic germline alterations: six (3.3%) with leukemia predisposition syndromes (*TP53, CEBPA, DNMT3A*, trisomy 21) and three (1.6%) with solid tumor predisposition syndromes (*MSH6, PALB2, SDHA*). Trio-WGS identifies one likely-pathogenic de novo *DNMT3A* variant. Six of nine diagnoses are previously unrecognized. Findings lead to tailored surveillance in 8/9 patients and treatment modifications in 4/9. Here, we show that this strategy gives a modest diagnostic yield. Nevertheless, the actionability of the findings supports its feasibility and validity in practice. Whether to restrict analysis to leukemia-relevant genes or broaden the panel should reflect local resources and counseling capacity.

## Abstract (web summary)

The potential of germline whole-genome sequencing (WGS) in detecting childhood leukemia predisposition remains to be elucidated. Here the authors show that a three-pronged diagnostic strategy including systematic phenotyping, germline WGS based 189-gene panel and tumor sequencing of positive cases may be feasible and clinically useful for cancer predisposition screening in childhood acute leukemia.

## Introduction

Leukemia represents the most common pediatric malignancy, accounting for approximately one-third of all childhood cancer cases worldwide^1,2^. Acute lymphoblastic leukemia (ALL) comprises approximately 80% of cases, with incidence peaking between two and five years of age^2–5^, while acute myeloid leukemia (AML) represents approximately 15-20% of pediatric leukemia cases, with a bimodal age distribution in childhood^6^. In Sweden, approximately 100 children are diagnosed with leukemia each year^7^.

While most leukemia cases arise sporadically, large-scale genomic studies involving analysis of 100 or more genes in cohorts exceeding 50 cases of pediatric leukemia have estimated that germline predisposition underlies 4–10% of pediatric hematological malignancies^8–12^. The prevalence varies significantly by subtype, with germline variants identified in 12–19% of childhood AML, compared to approximately 3% of childhood ALL^13–15^. Whether this discrepancy reflects true biological differences or arises from methodological factors -such as study design, cohort size, gene selection, or variant interpretation criteria—remains to be determined^8–11^.

To date, 45 genes have been associated with childhood leukemia predisposition^16^. These genes commonly affect core cellular pathways, including DNA repair (e.g., *ATM, NBN*, Fanconi anemia genes), apoptosis and cell cycle regulation (e.g., *TP53*), cellular signaling (e.g., *CBL, NF1, SOS1*), and hematological cell differentiation (e.g., *ETV6, RUNX1, IKZF1, PAX5*)^16–20^. Constitutional trisomy 21 (Down syndrome) confers elevated risk for both acute lymphoblastic and myeloid leukemias mediated by dosage-sensitive effects on hematopoiesis and cooperating somatic mutations^21,22^. Predisposing variants can be categorized as overt cancer predisposition syndromes (CPSs) which are associated with recognizable phenotypic features or as covert syndromes which present with subtle or no distinctive features^16^. Recognition of these genetic syndromes has led to their classification as distinct entities in the fifth editions of the World Health Organization (WHO) Classification of Haematolymphoid Tumors and WHO Classification of Pediatric Tumors, as well as in the 2022 International Consensus Classification of Myeloid Neoplasms and Acute Leukemias^5,23,24^. Consequently, recent clinical perspectives emphasize the systematic integration of germline testing into standard pediatric oncology workflows to ensure these patients receive optimized surveillance and personalized therapeutic interventions^25^.

Despite increasing recognition of germline predisposition in childhood leukemia, prospective population-based studies remain essential to study diagnostic strategies and clinical implications in routine care. The nationwide Genomic Medicine Sweden (GMS) Childhood Cancer Predisposition (ChiCaP) study is designed to evaluate the diagnostic yield and clinical utility of a three-pronged diagnostic strategy - systematic phenotyping, germline WGS-based analysis of a 189-gene panel, and tumor sequencing of positive cases - uniformly applied across Sweden and offered to all children at cancer diagnosis in Sweden^26^. Here, we show that our three-pronged diagnostic strategy is feasible and clinically valid for universal cancer predisposition screening in childhood acute leukemia. In the Swedish nationwide, population-based, prospective, multi-center study of 181 patients, this integrated approach identifies pathogenic germline alterations in nine patients (5%; 3.3% leukemia predisposition, 1.7% solid tumor predisposition), six of whom are previously unrecognized, prompting tailored surveillance in eight and treatment modification in four. Although the diagnostic yield is modest, the findings are clinically actionable and support implementation of this strategy in routine clinical practice.

## Results

### Baseline characteristics

**Fig. 1:**
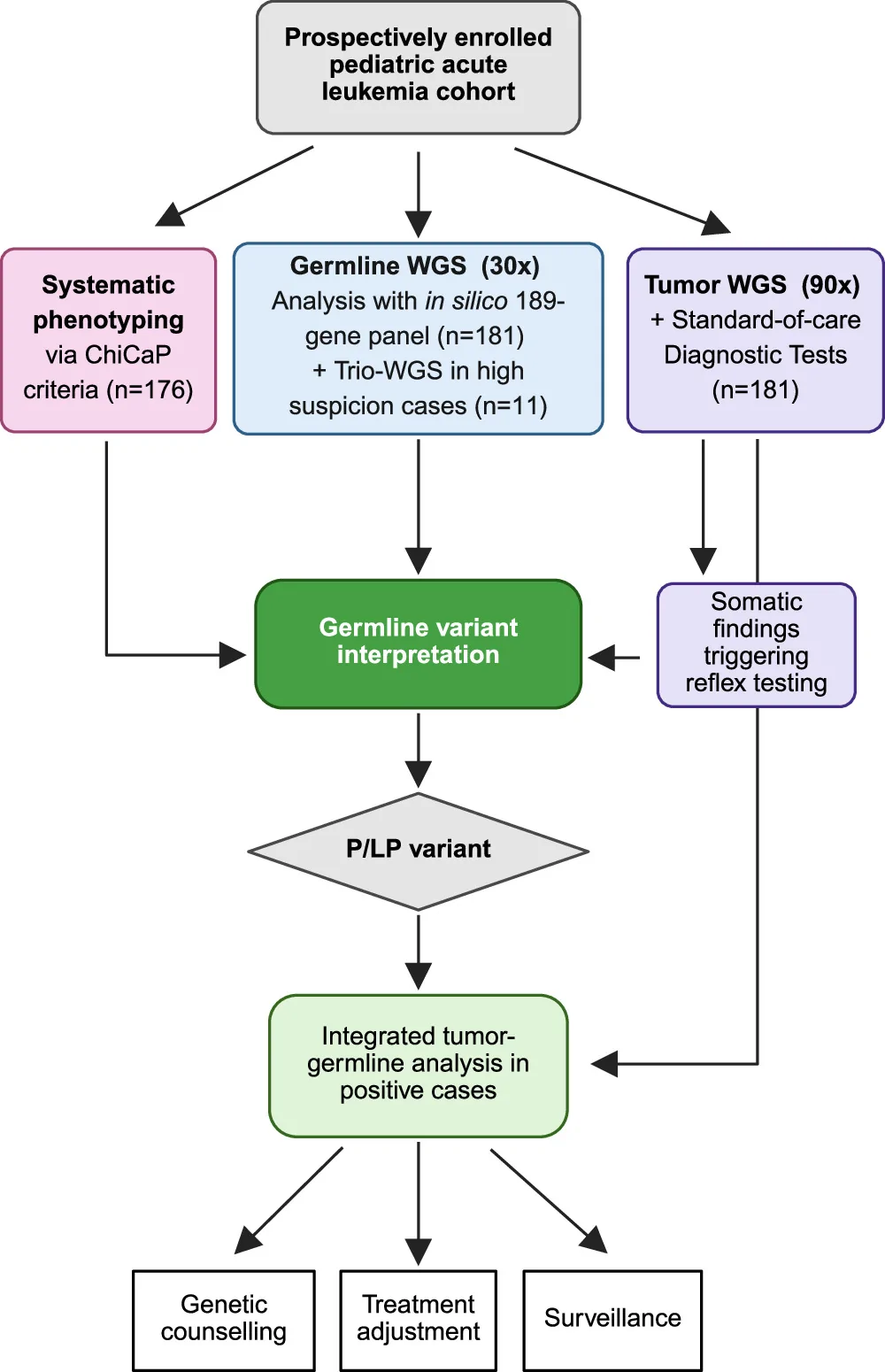
Three-pronged diagnostic strategy. The strategy combines systematic phenotyping, germline WGS-based analysis of a 189-gene panel, and analysis of tumor sequencing data of positive cases. Children with newly diagnosed acute leukemia (*n* = 181) were prospectively enrolled and evaluated through three parallel arms. Systematic clinical phenotyping was performed in 176 patients using the childhood cancer predisposition (ChiCaP) criteria. Germline whole-genome sequencing was undertaken in 181 patients and analyzed with an in silico 189-gene panel. Trio-WGS was performed in 11 cases with a high suspicion of a predisposition. In parallel, tumor WGS (90x coverage) was performed alongside standard-of-care diagnostic testing in the same patients; somatic findings suggestive of an underlying germline variant triggered reflex testing. Phenotypic, germline, and reflex-derived data were jointly considered during germline variant interpretation to identify pathogenic (P) or likely pathogenic (LP) variants. In patients harboring a P/LP variant, integrated tumor-germline analysis was performed to refine clinical actionability, informing genetic counseling, treatment adjustment, and long-term surveillance recommendations for patients and at-risk relatives. Created in BioRender. Taylan, F. (2026) https://BioRender.com/qmt8k5v.

**Fig. 2:**
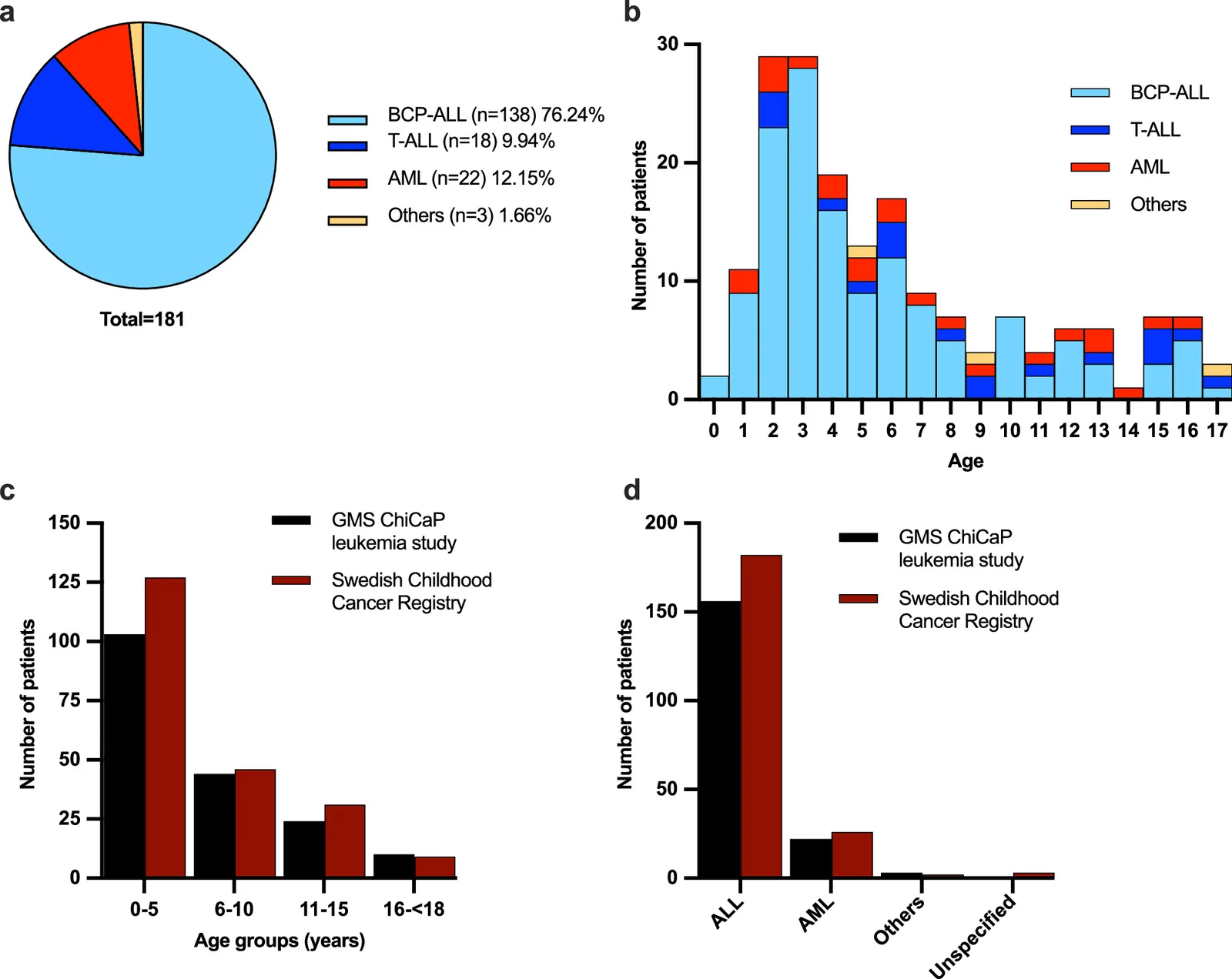
Overview of the GMS ChiCaP cohort in this study. **a** Pie chart of different leukemia types included and their share in the cohort (*n* = 181). The subtypes included in this study are B-cell precursor acute lymphoblastic leukemia (BCP-ALL; Blue, *n* = 138, 76.24%), acute myeloid leukemia (AML; Dark blue, *n* = 22, 12.15%), T-cell ALL (T-ALL; Red, *n* = 18, 9.94%), and other acute leukemias of ambiguous lineage (Yellow, *n* = 3, 1.66%) which are two mixed-phenotype acute leukemia (MPAL) and one acute undifferentiated leukemia (AUL). **b** Age distribution of patients (*n* = 181) in relation to their leukemia diagnosis. Comparison between GMS ChiCaP leukemia study cohort (Black) (*n* = 181) and leukemia diagnosis made in Sweden during the study period according to aggregated data from the Swedish Childhood Cancer Registry (SCCR; Dark red) (*n* = 213), grouped according to (**c**) age groups and (**d**) disease groups. Source data are provided as a Source data file.

Between 17 December 2021 and 30 April 2024, 181 children diagnosed with acute leukemia across all six pediatric oncology centers in Sweden were consecutively enrolled and investigated through our three-pronged diagnostic strategy (Fig. 1). Median age at diagnosis was 5 years (range 0–17), and 74 (41%) were female (Table 1). The most common diagnoses were B-cell precursor acute lymphoblastic leukemia (BCP-ALL; *n* = 138, 76%), followed by acute myeloid leukemia (AML; *n* = 22, 12%), T-cell ALL (T-ALL; *n* = 18, 10%), and acute leukemias of ambiguous lineage (ALALs) (*n* = 3, 2%) (Table 1, Fig. 2a). Distribution of ages showed a peak at 2–3 years which is driven by BCP-ALLs (Fig. 2b). At study enrollment, we collected family history of cancer, malignancy type, and clinical features suggestive of ChiCaP using the structured ChiCaP criteria form described in detail by Tesi et al.^26^ and in the Supplementary Information. Complete ChiCaP criteria forms were available for 174 of the 181 enrolled patients (96%) (Table 1).

**Table 1:** Patient characteristics (*n* = 181)

Characteristics	N	Overall (%)	95% CI
Age at diagnosis^a^	181	5.0 (3.0–8.5)	
Biological sex	181		
Female		74 (41%)	
Male		107 (59%)	
Leukemia origin	181		
Lymphoid		156 (86%)	
Myeloid		22 (12%)	
Ambiguous		3 (2%)	
≥1 positive ChiCaP criteria^b^	174	79 (45.4%)	38.1–52.8%
Family history for cancer	172	50 (29%)	22.8–36.3%
Multiple primary tumors	174	3 (1.7%)	0.5–4.9%
≥1additional features	173	29 (16.8%)	11.9–23.0%
≥2 additional features	173	8 (4.6%)	2.4–8.9%
≥2 positive ChiCaP criteria	174	10 (5.7%)	3.2–5.7%
P/LP variants in known cancer predisposition genes/syndromes	181	9 (4.9%)	2.6–9.2%
bona fide ChiCaP diagnosis^c^	181	6 (3.3%)	1.5–7.0%
Secondary findings	181	3 (1.6%)	0.5–4.8%

Only criteria 1–3 were evaluated in this study. Positive family history of cancer was defined as meeting at least one of the following across three generations: parental consanguinity; ≥1 relative with cancer before age 18; a parent or sibling with cancer before age 50; ≥2 relatives on the same side with cancer before age 50; ≥1 relative with multiple cancers; or ≥1 relative with the same cancer type as the child. Detailed descriptions of the ChiCaP criteria and questions to assess the family history of cancer were previously described by Tesi et al.^26^ and in the Supplementary Information. Binomial 95% confidence intervals for proportions were calculated using the hybrid Wilson/Brown method in GraphPad Prism version 11.0.2. Source data are provided as a Source Data file.

*N* number of patients.

^a^Median (IQR; interquartile range), *n* (%); *P* pathogenic, *LP* likely pathogenic, *CI* Confidence interval.

^b^ChiCaP criteria form^26^ was used to collect information categorized into four criteria: (1) family history (familial cancer or parental consanguinity); (2) multiple primary malignancies and/or severe treatment toxicity in the child; (3) other features (non-malignant phenotypes suggestive of a predisposition syndrome); and (4) malignancy type (tumor types enriched for genetic predisposition).

^c^Two Down syndrome and one mosaic trisomy 21 were clinically diagnosed prior to leukemia onset; three leukemia-associated predisposition syndromes were newly identified through this study (*TP53, CEBPA, DNMT3A*).

All samples were analyzed for variants in 189 established ChiCaP genes^26^ (Supplementary Data 1). Germline DNA was obtained primarily from cultured skin fibroblasts (*n* = 175, 97%). In six patients, a peripheral blood (*n* = 2) or bone marrow (*n* = 4) sample drawn at remission was used. To evaluate the diagnostic benefit of broader genetic testing, patients with negative analysis results but a strong clinical suspicion of ChiCaP (family history of any cancer (*n* = 6); non-malignant features suggestive of an underlying syndrome (*n* = 3); family history of cancer with multiple primary tumors (*n* = 1); and family history of cancer and non-malignant features (*n* = 1)), trio WGS - sequencing of proband (i.e., the child diagnosed with leukemia) alongside both parents —was performed in 11 cases (6 BCP-ALL, 3 T-ALL, 2 AML).

The Swedish Childhood Cancer Registry (SCCR) which captures ≥93% of cancer diagnoses in patients <16 years^7^ was used only for comparison of population coverage and representativeness. According to the SCCR, there were 213 first diagnoses of leukemia in children during the study period, while only 181 patients were included in the study, mainly due to logistic challenges in obtaining written informed consent. However, the age- (Fig. 2c) and disease-group (Fig. 2d) distribution of GMS ChiCaP participants closely matched the SCCR distribution for the study period, supporting that the cohort is broadly representative of the national childhood leukemia population.

### Frequency and Spectrum of Germline Variants

**Fig. 3:**
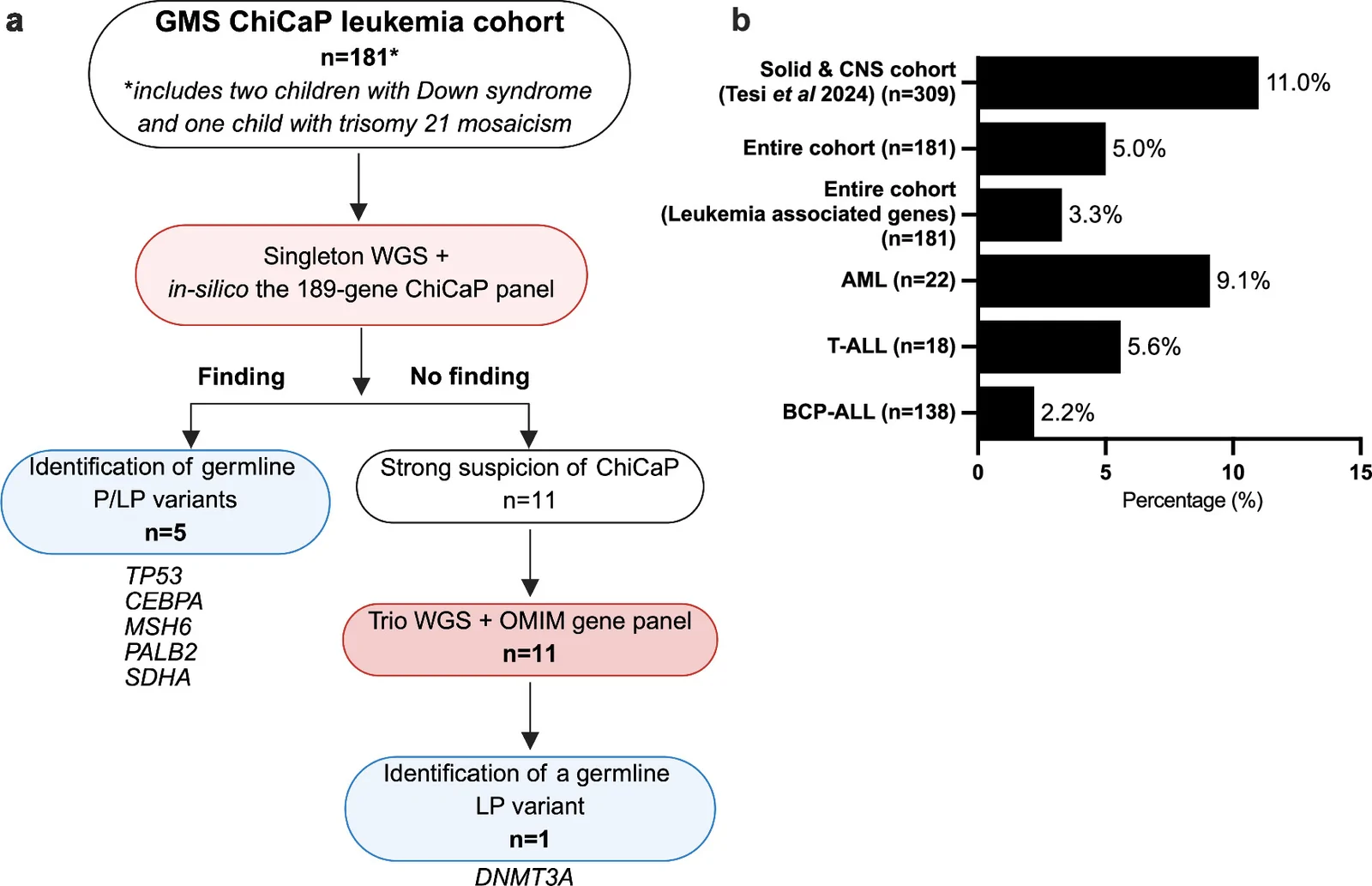
Germline findings. **a** Germline analysis workflow and yield in the GMS ChiCaP leukemia cohort (red boxes indicate analysis steps). A total of 181 children (including two with Down syndrome and one with trisomy 21 mosaicism) underwent singleton whole-genome sequencing (WGS) with in silico analysis of a 189-gene ChiCaP panel. Five children carried P/LP variants detected by the panel (*TP53, CEBPA, MSH6, PALB2, SDHA*). Among cases with no panel finding but high clinical suspicion of ChiCaP (*n* = 11), trio WGS with an OMIM-curated gene set identified one additional germline LP variant (*DNMT3A*). Created in BioRender. Taylan, F. (2026) https://BioRender.com/jn1iio1**b** Prevalence of P/LP variants (*n* = 9) in ChiCaP genes in this study based on gene composition and leukemia subtype and comparison to Tesi et al.^26^. Source data are provided as a Source data file.

The overall germline analysis workflow and the pathogenic (P)/likely pathogenic (LP) findings in our cohort are summarized in Fig. 3a. In silico analysis using the 189-gene ChiCaP panel revealed a germline P/LP variant in 5 of 181 (2.76%; 95% CI 1.2-6.3%) cases. Additionally, trio WGS identified a de novo heterozygous variant in *DNMT3A* (NM_022552.5: c.2635 A > G, p.(Asn879Asp)), absent in both parents and classified as LP according to ACMG criteria. Three patients had already been diagnosed with Down syndrome at enrollment, including one with mosaic trisomy 21. A cancer predisposition syndrome was identified in 9 of 181 patients, corresponding to an overall diagnostic yield of 5.0% (95% CI 2.6–9.2%) (Table 1, Fig. 3b, Supplementary Data 2). Six patients (6/181, 3.3%; 95% CI 1.5–7.0%) carried genetic aberrations associated with established leukemia predisposition syndromes (Fig. 3b), whereas three patients (3/181, 1.7%; 95% CI 0.5–4.8%) harbored heterozygous P/LP variants in ChiCaP genes associated with adult-onset predisposition to solid tumors (Table 1). ChiCaP diagnoses were identified in 2 of 22 AML cases (9.1%; 95% CI 1.6–27.8%), 1 of 18 T-ALL cases (5.6%; 95% CI 0.3–25.8%), and 3 of 138 BCP-ALL cases (2.2%; 95% CI 0.6–6.2%) (Fig. 3b). Due to small sample sizes in the AML and T-ALL subgroups, statistical comparison of prevalence across subtypes was not feasible.

Among the AML cases, one (SE_AL144) child had trisomy 21 (Down syndrome) without additional ChiCaP findings (Table 2). Another child (SE_AL154) carried a de novo heterozygous NM_004364.5(*CEBPA*):c.247del p.(Gln83Serfs*77) variant, classified as LP, suggesting predisposition to AML. Among T-ALLs, a child (SE_AL119) with syndromic features and T-ALL was diagnosed with Tatton-Brown-Rahman syndrome (TBRS; OMIM #615879), caused by a de novo heterozygous NM_022552.5(*DNMT3A*):c.2635 A > G (p.Asn879Asp) variant, classified as LP.

**Table 2:** Germline findings

Study ID	Leukemia diagnosis	Gene/Chromosome	Reference transcript	HGVS c.	HGVS p.	Genotype	ACMG classification	ChiCaP known at diagnosis	No. of positive ChiCaP criteria	Genetic counseling offered	Surveillance recommendation
SE_AL154	AML	*CEBPA*	NM_004364.5	c.247del	p.(Gln83Serfs*77)	Heterozygous de novo	LP	No	0	Yes	Yes
SE_AL144	AML	Trisomy 21	NA	NA	NA	NA	NA	Yes	1	Yes, before leukemia	Yes
SE_AL119	T-ALL	*DNMT3A*	NM_022552.5	c.2635 A > G	p.(Asn879Asp)	Heterozygous de novo	LP	No	1	Yes	Yes
SE_AL029	BCP-ALL	Trisomy 21 (Mosaic)	NA	NA	NA	NA	NA	Yes	2	Yes, before leukemia	Yes
SE_AL037	BCP-ALL	Trisomy 21	NA	NA	NA	NA	NA	Yes	1	Yes, before leukemia	Yes
SE_AL120	BCP-ALL	*TP53*	NM_000546.6	c.325 T > A	p.(Phe109Ile)	Heterozygous	LP	No	1	Yes	Yes
SE_AL027	BCP-ALL	*MSH6*	NM_000179.3	c.3261dup	p.(Phe1088Leufs*5)	Heterozygous (inherited from mother)	P	No	0	Yes	Yes, in adulthood
SE_AL162	BCP-ALL	*SDHA*	NM_004168.4	c.223 C > T	p.(Arg75*)	Heterozygous	P	No	0	No	No
SE_AL054	BCP-ALL	*PALB2*	NM_024675.4	c.2748+1 G > T	p.?	Heterozygous (inherited from father)	P	No	0	Yes	Yes, in adulthood

Each row represents a unique patient with germline findings (*n* = 9) in known ChiCaP genes.

*BCP-ALL* B-cell precursor acute lymphoblastic leukemia, *T-ALL* T-cell acute lymphoblastic leukemia, *AML* acute myeloid leukemia, *CNV* copy-number variant, *SNV* single-nucleotide variant, *INDEL* insertion–deletion (small insertion or deletion variant), *HGVS* Human Genome Variation Society, *ACMG* American College of Medical Genetics and Genomics, *LP* likely pathogenic, *P* pathogenic, *NA* not applicable.

Among BCP-ALL cases, one child (SE_AL037*)* had Down syndrome (trisomy 21) without additional ChiCaP findings. A second BCP-ALL patient (SE_AL120) had a Li–Fraumeni syndrome caused by a heterozygous germline NM_000546.6(*TP53*):c.325 T > A (p.Phe109Ile) variant (Table 2). No P/LP germline variants were identified in any of the six cases who were analyzed in blood/bone marrow remission samples with the 189-gene panel. However, 30x WGS analysis performed in one remission sample (SE_AL029) could not detect trisomy 21 mosaicism, previously found in approximately 90% of fibroblasts and in all leukemic cells. Three other BCP-ALL patients carried heterozygous P/LP variants in *MSH6, PALB2*, and *SDHA* - genes known for adult-onset solid tumor predisposition but not previously linked to childhood leukemia. The *MSH6* variant was maternally inherited with limited family cancer history (one colorectal cancer after age 50 years in a second-degree relative), while the *PALB2* variant was paternally inherited. On the paternal side, family history included breast cancer in the father’s cousin at age 50 and cervical, prostate, and colon cancers in different paternal relatives, all diagnosed after age 50. The *SDHA* variant was not considered actionable given its low penetrance and absence of family history of pheochromocytoma/paraganglioma, and no segregation analysis was performed.

Eight patients were offered genetic counseling. In addition to the individuals with Down syndrome, two children had de novo variants in *CEBPA* and *DNMT3A* respectively, while the *TP53* variant was inherited from a sperm donor^27^. In two families with inherited variants in *PALB2* and *MSH6*, family members were offered cascade testing, with surveillance offered to carriers.

### Somatic findings in cases with positive ChiCaP

Across ChiCaP-positive cases, WGS performed on leukemia cells identified large structural variants and canonical, subtype-specific drivers that were consistent with karyotypic abnormalities detected in standard-of-care investigations (details in Table 3). In the AML case (SE_AL154) with a germline heterozygous *CEBPA*, a somatic second hit causing a biallelic *CEBPA* disruption was observed. In the Down syndrome-associated AML (SE_AL144), the leukemic clone showed trisomy 21 with a truncating *GATA1* variant. In the T-ALL case (SE_AL119) harboring the germline *DNMT3A* variant, somatic focal 9p loss (including *PAX5*, and *CDKN2A*/*B)*, loss of *IKZF1* on 7p and partial *ETV6* deletion were detected.

**Table 3:** Clinical and genetic characteristics, treatment protocols, and surveillance recommendations for patients with germline predisposition variants

Study ID/Germline finding	Diagnosis	WBC counts at diagnosis (x10^9^/L) and CNS status	Leukemia karyotype	Leukemia WGS analysis findings with diagnostic/prognostic importance	NCI (USA) risk group at diagnosis	Treatment change due to germline finding	Induction therapy	Treatment protocol
**SE_AL154** *CEBPA*	AML	100.5 CNS1	46,XX	NM_004364.5(*CEBPA*):c.937_939dup; p.(Lys313dup), VAF 45%	NA	No	NA	NOPHO-DBH-AML-2012 protocol
**SE_AL144** *Trisomy 21*	AML	6.2 CNS1	47,XY,+21c	NM_002049.3(*GATA1*):c.167_182delinsTAGGCCAGTGCG; p.(Ala56ValfsTer80), VAF 9%	NA	Yes	No cortisone	Myeloid leukemia Down syndrome 2006 protocol
**SE_AL119** *DNMT3A*	T-ALL	10 CNS2	46,XY,del(9)(p13.2p21.3),del(9)(q21.33q31.2)	Deletion *CDKN2A* (all exons), *CDKN2B* (all exons), *ETV6* (exon 2, NM_001987.5), *IKZF1* (all exons), *IKZF1* (exon 2 – 3) (NM_006060.6), *PAX5* (all exons)	Intermediate high risk	No	Dexamethasone	ALLTogether protocol
**SE_AL029** *Mosaic Trisomy 21*	BCP-ALL	NA NA	47,XX,del(7)(p11.2p15.1),del(16)(q21),+der(17)del(17)(p11.2),+21c	Deletion *BTG1* (all exons), *IKZF1* (all exons)	Intermediate high risk	Yes	Dexamethasone	ALLTogether Down IR protocol
**SE_AL037** *Trisomy 21*	BCP-ALL	1.1 CNS1	48,XY,der(12)(p13),+del(16)(q23),dup(17)(q21q23),+21c	Deletion *CDKN2A* (all exons), *CDKN2B* (all exons)	Intermediate risk	Yes	Dexamethasone	ALLTogether Down IR protocol
**SE_AL120** *TP53*	BCP-ALL	2.9 CNS1	39,XY,−3,−4,del(5)(q31q35),−7,−9,−13,−15,−16,−17, + 21	Deletion *CDKN2A* (all exons), *CDKN2B* (all exons)*, EBF1* (all exons), *IKZF1* (all exons), *PAX5* (all exons), *RB1* (all exons)	High risk	Yes	Dexamethasone	ALLTogether High Risk protocol
**SE_AL027** *MSH6*	BCP-ALL	2.8 CNS2	53,XX, + 4, + 6, + 8, + 14, + 17, + 21, + 21	Deletion *CDKN2A (*exon 1, NM_000077.5), *CDKN2B* (all exons), *PAX5* (exon 6 – 8, NM_016734.3)	Intermediate low risk	No	Dexamethasone	ALLTogether protocol
**SE_AL162** *SDHA*	BCP-ALL	16 CNS3	47,XX, + X,del(4)(q22.3q24),qdp(21)(q21.1q22.3)	Deletion *CDKN2A* (all exons), *CDKN2A* (exon 1 – 2, NM_000077.5), *CDKN2B* (all exons), *RB1* (exon 18 – 27, NM_000321.3)	Low risk	No	Dexamethasone	Individualized because of severe infections, received blinatumomab and inotuzumab, BK virus hemorrhagic cystitis
**SE_AL054** *PALB2*	BCP-ALL	4.6 CNS1	46,XX,der(11)t(9;11)(p21;q23),der(15)t(1;15)(p22;q26)	Deletion *PAX5* (exon 6 – 8, NM_016734.2)	High risk	No	Dexamethasone	ALLTogether protocol IR-high, randomized to Inotuzumab treatment prior to standard maintenance treatment

Each row represents a unique patient with identified P/LP germline variants (*n* = 9). Clinical parameters include diagnostic features, risk stratification, and treatment modifications.

*WBC* white blood cells, *CNS1* central nervous system status 1: ≤5 WBC/μL cerebrospinal fluid (CSF) without evidence of leukemic cells after cytocentrifugation, *CNS2* central nervous system status 2: ≤5 WBC/μL CSF with ≥2 identifiable leukemic cells per slide by morphology, *CNS3* central nervous system status 3: >5 WBC/μL CSF with identifiable leukemic cells or any neurological symptoms at diagnosis, *WGS* whole-genome sequencing, *VAF* variant allele frequency, *NCI* National Cancer Institute, *IR* intermediate risk, *NA* not available.

The Down syndrome BCP-ALL case (SE_AL037) harbored multiple somatic copy-number alterations, including *CDKN2A/CDKN2B* deletions, without any known driver SNV/indel. All leukemic cells in the individual (SE_AL029) with trisomy 21 mosaicism, harbored +21 in combination with *BTG1* and *IKZF1* deletions, consistent with the molecular landscape of Down syndrome-associated BCP-ALL^28–30^. This patient had been diagnosed with AML and mosaic trisomy 21 at age two, four years prior to BCP-ALL diagnosis. The genetic profile of the BCP-ALL - harboring *IKZF1* and *BTG1* deletions without *KMT2A* rearrangement - is inconsistent with a therapy-related origin and instead supports a predisposition-driven second primary leukemia. Although the mosaicism was undetectable in peripheral blood and remission bone marrow, analysis of 24 informative SNPs across chromosome 21 confirmed duplication of the same chromosome 21 parental homolog in fibroblasts, AML, and BCP-ALL, which is in line with a shared constitutional origin, although an independent recurrent event involving the same homolog cannot be formally excluded.

In the *TP53*-related low-hypodiploid BCP-ALL case (SE_AL120), leukemic cells exhibited extensive whole-chromosome losses and copy-neutral loss of heterozygosity (CN-LOH), including monosomy 17 with biallelic loss of *TP53*, together with deletions of *CDKN2A*/*B*, *IKZF1, EBF1, PAX5*, and *RB1*. The germline *TP53* variant had a variant allele frequency (VAF) of 81%, consistent with loss of the wild-type chromosome 17.

Cases harboring germline variants in *MSH6*, *SDHA*, or *PALB2* each exhibited different BCP-ALL subtypes: the *MSH6* case was classified as high hyperdiploid ALL, the *SDHA* case as iAMP21 ALL, and the *PALB2* case as *KMT2A*-rearranged ALL. The somatic profiles in these cases also included recurrent secondary alterations typical of BCP-ALL, such as deletions at *PAX5* and *CDKN2A*/*CDKN2B*. No second hit was identified in the respective cancer predisposition genes; case-level specifics are provided in Table 3.

### Clinical characteristics and treatment implications

The child with Down syndrome and AML (SE_AL144) was treated according to the Myeloid Leukemia of Down Syndrome 2006 protocol without modifications and the patient with *CEBPA*-associated familial AML (SE_AL154) received standard treatment with the NOPHO-DBH-AML-2012 regimen without adjustment (Table 3). Patient SE_AL119 with T-ALL underwent trio-WGS following negative results from the 189-gene ChiCaP panel, prompted by clinical suspicion of an underlying syndrome. The patient presented with hydrocephalus, Chiari-1 malformation, hydrocele, developmental delay, hypotonia, nystagmus, and recurrent infections. Trio WGS identified a de novo LP *DNMT3A* variant that explained both the syndromic features, establishing a diagnosis of TBRS, and the predisposition to T-ALL^31–34^. This association is consistent with reports of a 4% prevalence of hematopoietic malignancies in TBRS, representing a > 250-fold increased risk compared to the general population^33^. The patient was treated according to the ALLTogether intermediate-high-risk protocol without modifications (Table 3). The two BCP-ALL patients, Down syndrome (SE_AL037) and trisomy 21 mosaicism (SE_AL029), were both stratified to intermediate risk and treated according to the ALLTogether Down IR protocol. The patient (SE_AL120) with Li–Fraumeni syndrome was classified as high-risk based on somatic genetic findings (low hypodiploidy) and enrolled in the ALLTogether high-risk treatment protocol (Table 3).

### ChiCaP criteria assessment

**Fig. 4:**
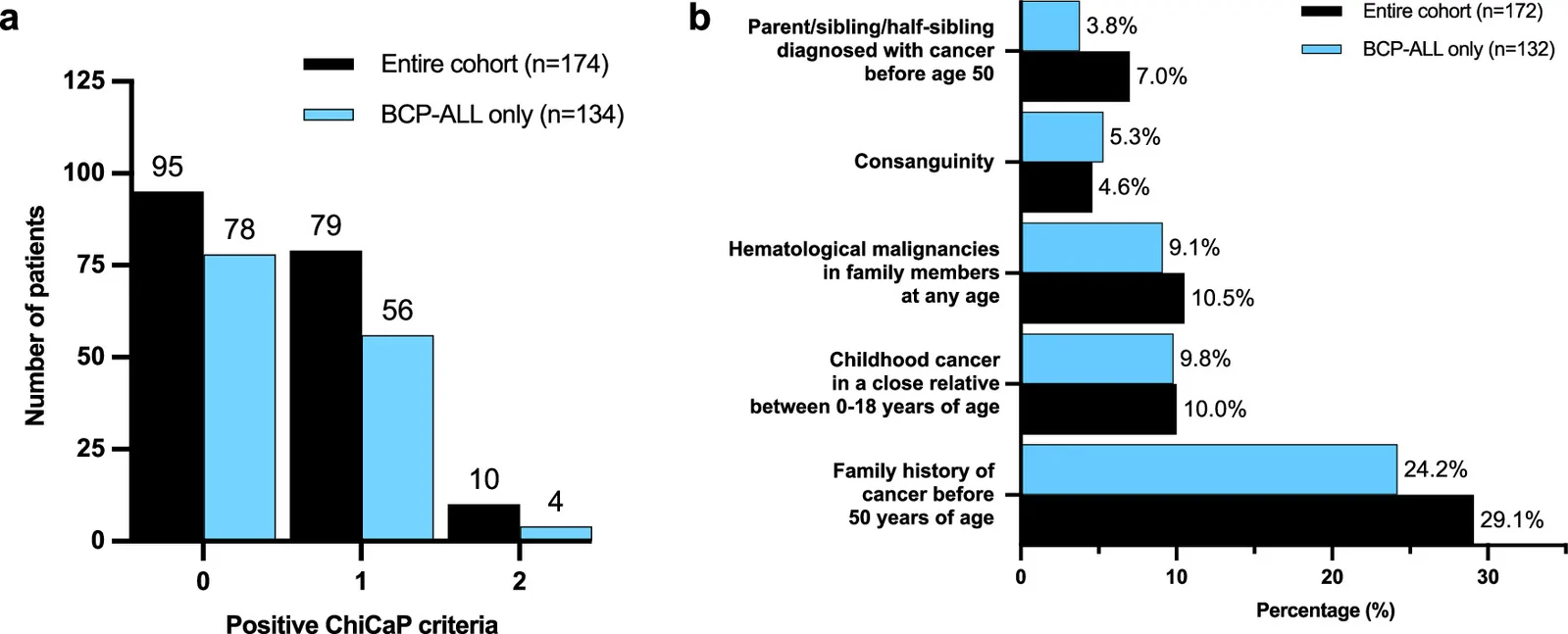
ChiCaP indicators. **a** Number of patients not fulfilling any criteria and fulfilling at least one or two ChiCaP criteria in the entire cohort (*n* = 174) and BCP-ALL only cohort (*n* = 134). No patient fulfilled 3 or 4 ChiCaP criteria. These include combinations of family cancer history, malignancy/toxicity history and non-malignant features. **b** Family history distribution in the entire cohort (*n* = 172) and BCP-ALL only cohort (*n* = 132). Percentage of patients with positive family history by category. Each bar represents the proportion of the total cohort (Black, *n* = 172) and BCP-ALL cohort (Blue, *n* = 132) with documented family history in the specified category. Family history of cancer before 50 years of age category includes at least two relatives with cancer before 50 years of age, cancer in a close relative before 18 years of age and first-degree relatives diagnosed with cancer before 50 years of age. Source data are provided as a Source data file.

Among the 174 patients with completed ChiCaP criteria forms^26^, 45.4% (79/174; 95% CI 38.2–52.8%) met at least one ChiCaP criterion, and 5.7% (10/174; 95% CI 3.2–10.3%) fulfilled two criteria (Table 1 and Fig. 4a). No patients met three or more criteria (Fig. 4a). Among patients with BCP-ALL, 41.8% (56/134; 95% CI 33.8–50.3%) fulfilled at least one criterion, and only 3.0% (4/134; 95% CI 1.2–7.4%) met two criteria (Fig. 4a). Positive family history of cancer was reported in 29.1% of patients (50/172; 95% CI 22.8–36.3%) across leukemia subtypes (Fig. 4b). Specifically, childhood cancer in a close relative (parent, sibling, or half-sibling) was noted in 10% (17/172; 95% CI 6.3–15.3%), and hematological malignancies in family members of any age were reported in 10.5% (18/172; 95% CI 6.7–15.9%). Consanguinity was present in 4.6% (8/172; 2.4–8.9%), and 7% of patients (12/172; 95% CI 4.0–11.8%) had a parent diagnosed with cancer before the age of 50 (Fig. 4b).

Among patients with BCP-ALL, a positive family history of cancer was reported in 24.2% of patients (32/132; 95% CI 17.7–32.2%) (Fig. 4b). Childhood cancer in a close relative was noted in 9.8% (13/132; 95% CI 5.8–16.1%), and hematological malignancies in family members of any age were reported in 9.1% (12/132; 95% CI 5.3–15.2%). Consanguinity was present in 5.3% of cases (7/132; 95% CI 2.6–10.5%). Additionally, 3.8% of patients (5/132; 95% CI 1.6–8.6%) had a parent diagnosed with cancer before age 50.

Multiple primary malignancies were documented in 3/174 patients (1.7%; 95% CI 0.5–4.9%). The first patient (SE_AL001) was initially diagnosed with BCP-ALL at 2 years of age and subsequently developed therapy-related acute myeloid leukemia (t-AML) 5 years later, at 7 years of age. The second patient (SE_AL060) was initially diagnosed with neuroblastoma at 5 years of age and subsequently developed t-AML during the same year, remaining 5 years of age at the time of the second diagnosis. The third patient (SE_AL029) was diagnosed with AML at age 2 and BCP-ALL four years later, at age 6. We conducted a comprehensive “tip-to-toe” clinical review to identify congenital malformations and other non-cancer symptoms using the ChiCaP criteria form. Among the 173 patients with a completed ChiCaP criteria form, at least one non-malignant feature was present in 16.8% (29/173; 95% CI 11.9–23.0%), and 4.6% (8/173; 95% CI 2.4–8.9%) had more than one. The most frequent features were cognitive or behavioral disorders (6.9%, 12/173; 95% CI 4.0–11.7%), congenital malformations (4.0%, 7/173; 95% CI 2.0–8.1%), and skin manifestations (3.5%, 6/173; 95% CI 1.6–7.4%). The two patients with non-mosaic Down syndrome were diagnosed prior to leukemia onset, while the patient with BCP-ALL was diagnosed with mosaic trisomy 21 at age 2 when the AML diagnosis was established. One patient had a known genetic condition, X-linked deafness type 2 (OMIM 304400) caused by a *POU3F4* variant, which has not been associated with cancer predisposition. This patient had no additional ChiCaP findings.

Among the six patients with genetically confirmed ChiCaP findings in leukemia-associated genes, four met the ChiCaP criteria based on syndromic features (trisomy 21, *DNMT3A*, *TP53*) (Table 3) and one also met the criteria due to a previous primary malignancy (AML in the child with mosaic trisomy 21).

The individual with a germline *TP53* variant presented with hypodiploid ALL and somatic *TP53* aberrations. This child also met the ChiCaP criteria due to an abnormal thorax shape, which we consider an incidental finding with no apparent relation to the *TP53* variant. Although the individual with familial *CEBPA*-associated AML did not meet ChiCaP criteria, paired tumor–normal analysis identified a pathogenic somatic *CEBPA* p.(Lys313dup), and subsequent testing revealed a germline *CEBPA* p.(Gln83Serfs*77).

In contrast, none of the three individuals with variants in established solid tumor predisposition genes met the ChiCaP criteria (Table 3). Notably, no child with leukemia and a positive family history carried a pathogenic variant in the ChiCaP 189-gene list.

## Discussion

In this nationwide prospective cohort of 181 children with newly diagnosed acute leukemia 3.3% (6/181; 95% CI 1.5–7.0%) harbored a childhood leukemia predisposition syndrome and 1.7% (3/181; 95% CI 0.5–4.8%) harbored pathogenic variants in solid tumor predisposition genes. Using a three-pronged approach uniformly applied across Sweden, we identified cancer predisposition syndromes in 5.0% (9/181; 95% CI 2.6–9.2%) of leukemia patients overall, often previously unrecognized and with direct clinical implications. These findings indicate a modest but clinically actionable yield in an unselected population of childhood acute leukemias.

Previous pan-cancer pediatric studies have reported overall cancer predisposition syndrome prevalences of 7–18%^8–10,18,26,35–38^, but most of these estimates derive from targeted panels or pre-selected cohorts. Also, these figures encompass all tumor types and varying definitions, including adult-onset cancer predisposition syndromes, variants without established causality to the child’s neoplasm, and in some studies, heterozygous carriers of autosomal recessive genes^8–11^. When the comparison is restricted to P/LP variants in genes with an established association with childhood leukemia and matching inheritance pattern—the definition used for ChiCaP syndromes in our study -, the prevalence across studies narrows to 0.5–8.1%, and drops further to 0.5–4% when Down syndrome is excluded, with our 1.7% falling within both of these ranges (Supplementary Table 1). Overall, current data seem to point to a relatively low ChiCaP prevalence in childhood leukemia. In our cohort, prevalence appeared to vary by leukemia type, with AML showing a higher rate (2/22 cases) compared with BCP-ALL (2.2%, 3/138), although the small sample sizes preclude definitive conclusions.

Although the number of children diagnosed with a ChiCaP syndrome was modest, the clinical benefit of identifying a leukemia predisposition at the individual level was substantial. Only 3/9 ChiCaP diagnoses (constitutional and mosaic trisomy 21) were known prior to leukemia diagnosis. The ChiCaP findings directly influenced treatment in 4/9 of the cases, with management aligned to established protocols for Down syndrome and *TP53*-related cancer predisposition disorders and led to gene specific implementation of surveillance recommendations in 88% (8/9) of cases (Table 3). In addition, the ChiCaP results complemented somatic genetic findings; integrating germline data with leukemia karyotypes and clinical phenotypes revealed associations that refined risk assessment and guided genetic counseling. An illustrative example is the *TP53* (c.325 T > A) variant, previously reported in five pediatric patients across malignancies, including hypodiploid BCP-ALL^27^. Li–Fraumeni syndrome is associated with poor treatment outcome in ALL and an elevated risk of therapy-related secondary malignancies^39,40^. Similarly, detection of a somatic *CEBPA* variant should prompt germline assessment to exclude hereditary predisposition^41–45^. These findings underscore the clinical value of routine germline evaluation even when overall prevalence is modest.

A separate category of findings comprised three patients carrying P/LP variants in solid-tumor predisposition genes (*MSH6, SDHA, PALB2*) who met no ChiCaP criteria; their family cancer histories, where present, exceeded the age-of-onset thresholds, and these patients would have been missed, had patient selection relied solely on phenotype-driven triage with reflex testing. A review of the existing literature reveals no confirmed leukemia-associated monoallelic germline variants in these three genes. Hematological malignancy associated with *MSH6* and *PALB2* has predominantly been described in the context of biallelic inactivation^46,47^, whereas evidence for childhood leukemia in monoallelic carriers is limited to isolated case reports^10,48,49^ and a comprehensive review of germline *SDHA* variants across cancer types found no cases of hematological malignancy among affected individuals^50,51^. Taken together with the absence of second hits in the leukemias of these patients, the lack of established evidence linking heterozygous variants in these genes to childhood BCP-ALL supports the interpretation that these findings are likely unrelated to leukemia development^52^. Larger cohorts and functional studies are required to establish whether leukemia falls within the phenotypic spectrum associated with these solid tumor predisposition genes. Importantly, identifying the *MSH6* and *PALB2* variants nonetheless conferred immediate clinical benefit through risk-appropriate surveillance for the associated solid tumor predisposition syndromes and by facilitating genetic counseling with cascade testing of at-risk relatives.

Restricting attention to the leukemia-associated ChiCaP syndromes, all six genetically confirmed cases would have been triaged for germline analysis under a phenotype-driven diagnostic strategy, either through fulfillment of established clinical criteria or through somatic findings prompting reflex testing, in accordance with current diagnostic guidelines and published recommendations^23,24,40,45,53–56^. However, this observation is contingent on systematic somatic profiling of the leukemia at diagnosis, since reflex testing depends on the detection of informative somatic variants. We did not perform a direct prospective comparison between phenotype-driven triage and phenotype-agnostic germline sequencing, an analysis recently undertaken by Bakhuizen and colleagues in a pediatric pan-cancer cohort^11^, who demonstrated phenotype-driven genetic testing and phenotype-agnostic CPS gene panel sequencing to be complementary. These considerations support a phenotype-driven strategy as a pragmatic and resource-efficient framework, particularly in settings where universal germline sequencing is not feasible, provided that it is coupled with thorough somatic workup of the leukemia. However, several limitations of a phenotype-driven approach nevertheless warrant emphasis: variants are expected to escape detection in patients with (i) de novo variants without supportive family history, (ii) variants in low-penetrance genes, (iii) absent or as-yet-unrecognized phenotypic features, and (iv) insufficient tumor material for somatic analyses. Heterozygous variants in genes associated with adult-onset predisposition - findings beyond the scope of the analysis would also be missed by a phenotype-driven approach. On the other hand, a universal genotype-first approach, in which sequencing is performed without prior phenotypic anchoring, may result in lower-quality variant interpretation, which depends critically on detailed phenotypic information. Instead, phenotypic information is integral to our three-pronged strategy, ensuring high-quality variant interpretation.

Several observations in our cohort indicate that the spectrum of leukemia predisposition extends beyond what current ChiCaP gene panels capture. Approximately 10% of children in our cohort had a reported family history of childhood cancer and 9% had relatives with hematological malignancies at any age, suggesting undiagnosed predisposition in a proportion of cases. In our previous study of the children with solid/CNS tumors, 66% (194/293) met at least one ChiCaP criterion and among those 17% (33/194) received a ChiCaP diagnosis using the same gene panel as we have used in this study^26^. In the present leukemia cohort, 45.4% (79/174) met at least one ChiCaP criterion and 6.3% (5/79) of these had a confirmed leukemia-associated ChiCaP syndrome. The lower yield among criteria-positive leukemia patients, combined with the family history data, supports the interpretation that ChiCaP syndromes caused by currently known ChiCaP genes are infrequently identified in leukemia and that undiscovered leukemia predisposition syndromes likely exist. The low overall prevalence in BCP-ALL (2.2%) may, however, be consistent with previous hypotheses that most cases arise from somatic events—chromosomal rearrangements, aneuploidy, and gene fusions - during early development or in response to environmental factors, including infections during immune maturation in early childhood^57,58^.

The identification of a de novo LP *DNMT3A* variant in a patient with T-ALL and a clear TBRS phenotype is in line with emerging evidence that germline *DNMT3A* variants may predispose to hematological malignancies in children^31,33,34^ and illustrates the value of trio-WGS in patients with syndromic features of undetermined etiology, where panel-based approaches remain constrained by the timeliness of gene content updates and may fail to capture clinically relevant variants in genes not yet incorporated into curated lists. The patient with mosaic trisomy 21 and BCP-ALL illustrates how low-level mosaicism in the hematopoietic compartment can confer clinically significant leukemia predisposition that may be overlooked when only single tissue is analyzed, underscoring the importance of examining multiple tissues with appropriate methodologies when the clinical phenotype or somatic findings raise suspicion of ChiCaP.

A key strength of this study lies in its three-pronged diagnostic strategy uniformly applied across centers, integrating systematic clinical phenotyping, germline WGS analyzed through the 189-gene panel, and analysis of tumor data of cases harboring candidate predisposing variants. This multimodal framework was applied to nearly all children diagnosed with acute leukemia in Sweden during the study period, reflecting the unselected, prospective, and nationwide design of the GMS-ChiCaP cohort. The use of fibroblast-derived DNA as the germline source in the majority of cases further strengthens the analytical validity of our findings. Although the pathogenic variants identified in this study were confined to exonic regions and would have been detectable by exome sequencing or targeted panels, WGS enables detection of variant classes beyond the reach of exome-based approaches.

Limitations include reliance on a predefined in silico gene panel, which may miss relevant variants outside the curated list; failure to detect mosaic variants present at levels below approximately 20%; and the exclusion of novel gene discovery approaches apart from the 11 patients in whom trio WGS was performed even though this was not within the scope of our study. Reasons for declining study participation were not systematically recorded during the consent process, limiting our ability to fully characterize non-participation. The small T-ALL and AML cohort sizes constrain statistical power. In approximately 3% of the cohort, germline DNA was derived from remission blood or bone marrow rather than fibroblasts, which in principle carries a risk of somatic-to-germline misclassification; apart from the case with mosaic trisomy 21, none of these patients harbored a germline finding. Although cultured skin fibroblasts remain the preferred germline source in hematological malignancies because the culture process eliminates contaminating hematopoietic cells, cultures typically require 3–4 weeks from biopsy to DNA extraction^59^, contributing to the inherent delay in germline analysis in this clinical setting.

Looking ahead, trio WGS remains particularly advantageous, for resolving previously undiagnosed syndromes and sequencing of parents supports segregation analysis that reduces the interpretive burden associated with the large number of variants of uncertain significance. Large, harmonized multicenter studies pairing standardized phenotyping with comprehensive omics—including long-read trio-WGS, deep exome sequencing for mosaicism detection, transcriptomics, epigenomics, functional assays and proteomics—combined with systematic somatic-germline integration, will be essential to advance ChiCaP diagnostics, refine risk stratification, and identify new leukemia predisposition genes.

In conclusion, we demonstrate that our three-pronged diagnostic strategy is feasible in clinical practice and valid for universal cancer predisposition screening in childhood acute leukemia, and we therefore advocate its implementation where resources allow. Because the genetic basis of leukemia predisposition is highly heterogeneous, focused germline gene panels restricted to actionable cancer predisposition genes are unlikely to capture the full spectrum of ChiCaP diagnoses. The choice of testing strategy should accordingly reflect local policies and resources, including the capacity for pre-test genetic counseling, as well as the decision of whether to restrict analysis to genes directly relevant to the child’s cancer or to extend it to a broader set encompassing solid tumor predisposition genes.

## Methods

### Ethical approval and written informed consents

Ethical approval was obtained from the Swedish Ethical Review Authority (Etikprövningsmyndigheten), the single national authority responsible for ethical review of research involving human participants in Sweden. Under Swedish law (the Ethical Review Act, SFS 2003:460), approval from this authority applies to all participating sites; no separate site-specific approvals are required or issued by individual hospitals or regions. Accordingly, the following permits cover sample and data collection at all six participating pediatric oncology centers (Astrid Lindgren Children’s Hospital, Karolinska University Hospital; Akademiska Children’s Hospital, Uppsala University Hospital; Crown Princess Victoria Children’s Hospital, Linköping University Hospital; Queen Silvia Children’s Hospital, Sahlgrenska University Hospital; Childhood Cancer Center, Skåne University Hospital; and Norrlands University Hospital, Umeå): 2021-04135 (tumor–normal WGS analysis) and 2019-04746 (trio WGS analysis). Register data were analyzed under ethical permits 2018/1849-32 and 2023-06901-01. Written informed consent was obtained from all participants, either from legal guardians or for patients aged ≥15 years, directly from the patients themselves. Participants received no compensation. Study information and counseling were provided by trained clinicians in pediatric hemato-oncology and clinical genetics. Consent included the disclosure of germline findings consistent with ChiCaP syndromes, as well as other actionable incidental findings when identified.

### Study design, cohort description, and workflow

Children under 18 years of age (median age at diagnosis 5 years, IQR [3.0-8.5]; [*n* = 74 female, *n* = 107 male]) diagnosed with acute leukemia between 17 December 2021 and 30 April 2024 at one of the six pediatric oncology centers in Sweden were eligible for inclusion. Families were approached for participation at the time of leukemia diagnosis. Participation required written informed consent and the availability of germline DNA from either cultured skin fibroblasts (from skin biopsy), remission blood, or remission bone marrow sample. In addition, blood samples from both parents were collected. To account for the possibility of dual diagnoses, all children diagnosed with cancer during the study period were included regardless of pre-existing genetic predisposition syndromes, as patients may harbor additional, previously unrecognized pathogenic germline variants. Not all eligible patients could be enrolled primarily due to logistic challenges in obtaining written informed consent.

Baseline characteristics were recorded for all participants, including sex, age at diagnosis and diagnosis type. Sex was obtained from medical records and verified using sex-chromosome dosage from WGS. Sex and gender were not analyzed as variables, as germline cancer-predisposition variants were evaluated irrespective of sex and gender.

At enrollment, pre-test genetic counseling was performed and a standardized clinical assessment for potential underlying ChiCaP was conducted using a form adapted from established screening tools for all types of childhood cancers, as described by Tesi et al.^26^ and in the Supplementary Information. Information was categorized into four ChiCaP criteria: (1) family history, including familial cancer or parental consanguinity; (2) multiple primary malignancies and/or severe toxicity to treatment in the child; (3) other features, including non-malignant phenotypes suggestive of a predisposition syndrome; and (4) malignancy type, based on tumor types known to be enriched for genetic predisposition^26^. Positive family history of cancer was defined as meeting at least one of the following criteria across three generations (parents, siblings, parents’ siblings, cousins, grandparents, and grandparents’ siblings): consanguinity of the child’s parents; at least one relative with cancer before age 18; a parent or sibling/half-sibling with cancer before age 50; at least two relatives on the same side of the family with cancer before age 50; at least one relative with multiple cancers; or at least one relative with the same type of cancer/tumor as the child. For this study, only criteria 1, 2, and 3 were evaluated. A supplementary ChiCaP criteria form was completed exclusively for children who exhibited an unusually severe toxicity reaction or were diagnosed with one of the following conditions: AML or MDS with karyotype monosomy 7/7q-, BCP-ALL with low hypodiploidy (31–39 chromosomes), or BCP-ALL with iAMP21. Fulfilling at least two of the main criteria, one main criteria with two or more sub-criteria or a somatic variant triggering reflex testing was regarded as high-risk clinical criteria.

While patients were recruited across six centers, germline and tumor genomic analyses were centralized to four regional genomics nodes—Gothenburg, Lund, Stockholm, Uppsala - following local protocols. Results were returned in written form to the treating physicians and documented in patients’ medical records. If ChiCaP was diagnosed, the patient was referred for genetic counseling. Clinical follow-up data were obtained from chart review.

### Germline WGS and data analysis

Primary dermal fibroblasts were isolated from skin punch biopsies obtained simultaneously with diagnostic bone marrow sampling. Biopsies were enzymatically dissociated with collagenase type II (Worthington Biochemical Corporation) prior to plating. Fibroblasts were cultured either in AmnioMAX-II Complete Medium (Gibco, Thermo Fisher Scientific) or in an *in-house* medium comprising RPMI 1640 (Gibco, Thermo Fisher Scientific) and Ham’s F-10 Nutrient Mix (Gibco, Thermo Fisher Scientific), according to site-specific routine practice. Genomic DNA was extracted from cultured fibroblasts, remission blood, or bone marrow using standard protocols.

Germline WGS was performed at 30x coverage using clinical bioinformatic pipelines established for rare disease diagnostics across four national Clinical genomics nodes. Pipelines used in all nodes (Stockholm, Lund, Gothenburg and Uppsala) were described in detail in the Supplementary Information. Depending on local protocols, sequence alignment was performed to either the hg19 or hg38 human reference genome. In general, our bioinformatic pipelines for WGS were designed to detect SNVs, indels, CNVs, and SVs. For SNV and indel calling, the Stockholm and Uppsala nodes employed DeepVariant (v.1.2.0-v.1.6.0)^60^, a deep neural network-based germline variant caller, was employed. During candidate variant generation, DeepVariant requires a minimum of two reads supporting the alternate allele and a minimum variant-supporting read fraction of 0.12 for both SNVs and indels (the vsc_min_fraction_snps and vsc_min_fraction_indels parameters). While this means that variants with a VAF as low as approximately 12% may be considered as candidates, DeepVariant is trained on germline data where expected allele fractions cluster around 50% (heterozygous) or 100% (homozygous). Consequently, its sensitivity for mosaic variants decreases at lower VAFs and may vary with variant type and genomic context. The Gothenburg and Lund nodes used Sentieon DNAscope (version 202308.01), a machine learning-based germline variant caller. In the Lund pipeline, a hard VAF filter of 20% was applied, excluding any variant below this threshold; no such hard filter was applied in the other pipelines. As all callers used in this study are designed and trained for germline variant detection, reliable detection of mosaic variants below approximately 20% VAF cannot be guaranteed, and sensitivity may further vary with variant type, genomic context, and local sequencing coverage.

Variant filtering was restricted to 189 established cancer predisposition genes (Supplementary Data 1) included in the ChiCaP panel, which was designed in 2020 based on stringent evidence criteria. The gene list followed certain rules such as replicated links to cancer risk, high or well-documented penetrance, and supporting segregation or functional data, as well as supporting evidence from at least five unrelated affected individuals^61^. The panel was version-locked in 2020 and applied unchanged to all samples. Evidence for leukemia risk in TBRS was only emerging, with three hematological cases reported by 2021^32,33^; therefore, *DNMT3A* was not included. Variant assessment followed routine clinical diagnostic practice at each center, focused on coding and splice-region variants within the gene panel. Predicted splice effects were assessed using in silico tools, including SpliceAI (generated as a separate annotation within the analysis pipelines where available) and the splice-prediction algorithms implemented in Alamut^TM^ Visual Plus (SOFiA GENETICS), alongside population allele frequencies (gnomAD). Review was extended to deeper intronic regions on a case-by-case basis where clinical suspicion warranted, or where variants had previously been reported as (likely) pathogenic in ClinVar. No clinically significant deep intronic variants were identified.

SNVs, insertions/deletions (INDELs), CNVs and structural variants (SVs) were interpreted by clinical laboratory geneticists and clinical geneticists according to guidelines from the American College of Medical Genetics (ACMG)/Association for Molecular Pathology (AMP) or gene-specific guidelines, considering the syndrome’s known inheritance pattern^62,63^. We established harmonized stringent variant reporting criteria to be used for variants identified in the ChiCaP study: P and LP variants scored as ACMG class 4 (LP) and 5 (P) were reported if: (1) they fitted the known inheritance pattern for the specific ChiCaP syndrome (e.g., biallelic P/LP variants in *ATM*) or constituted heterozygous P/LP variants in genes causative of high-risk dominant adult-onset cancer predisposition syndromes (e.g., *BRCA1/2*, Lynch syndrome, *PTEN*, etc.). Heterozygous variants in moderate-risk genes for adult-onset cancers (e.g., heterozygous P/LP variant in *ATM*) were not reported. Variants of uncertain significance defined as ACMG class 3 were reported only when both the variant characteristics indicated pathogenicity (i.e., not present in normal variation databases, high local ranking, compound variant present for autosomal recessive genes) and the patients´ phenotype had clinical presentation in line with the phenotype described for the affected gene. In these cases, segregation analysis with parental samples was performed—e.g., to prove de novo origin of the variant - prior to variant classification as P/LP.

For the six patients where a remission blood or bone marrow sample was used as the germline DNA source, candidate germline variants were cross-referenced against paired leukemia WGS data. Variant origin was assessed using variant allele fraction information to distinguish true germline variants from potential somatic contamination.

### Trio whole genome sequencing

In patients with positive ChiCaP criteria but a negative 189-gene panel result, trio WGS was performed where parental samples were available, extending the analysis a broader set of disease-associated genes comprising all OMIM^64^ Morbid genes known at the time of analysis (Supplementary Data 3). WGS was performed at Clinical Genomics, Stockholm, Sweden using the Illumina HiSeq X Ten or NovaSeq 6000 platforms, employing a 30x PCR-free paired-end WGS protocol. Genomic variants (SNVs and INDELs) were called using the Mutation Identification Pipeline (MIP; https://github.com/Clinical-Genomics/MIP)^65^, with DeepVariant as the variant caller, aligned to the GRCh37 (hg19) reference genome. In addition to SNVs and INDELs, the pipeline includes analysis of structural variants (deletions, duplications, and inversions), short tandem repeat expansions, and *SMN1* copy number assessment. Variants were filtered per family for de novo events, X-linked inheritance, autosomal dominant and autosomal recessive inheritance (homozygous or compound heterozygous). For autosomal recessive inheritance, two variants in trans in the same gene were required. Only sequence variants with an allele frequency below 1% in the general population, affecting exons or exon-intron boundaries, were considered. Called variants were annotated and prioritized using an in-house pathogenicity scoring system, genmod (https://github.com/Clinical-Genomics/genmod), that assigns a rank score based on multiple parameters including allele frequency, predicted protein impact, gene intolerance, inheritance model compatibility, variant consequence, evolutionary conservation, variant call quality, predicted deleteriousness, clinical significance, and splicing predictions. Variants classified as P/LP in ClinVar with a gold star review status were always retained regardless of frequency or consequence. A detailed description of the trio WGS analysis pipeline used here has been reported previously^65–67^. Confirmatory testing of identified variants was carried out using orthogonal methods, following established procedures at each participating laboratory.

### Tumor sequencing

DNA extracted from diagnostic bone marrow specimens underwent WGS analysis at 90x coverage using clinical bioinformatic pipelines established for tumor diagnostics across four national clinical genomics nodes. Pipelines used in all nodes (Gothenburg, Lund, Stockholm and Uppsala) were described in detail in the Supplementary Information.

A somatic finding with VAF above 40% in genes associated with leukemia predisposition triggered a reflex testing. Second-hit analysis was conducted on tumor samples from patients harboring P/LP germline variants in tumor suppressor genes. Second hits were categorized into three groups: (1) second mutations, comprising somatic SNVs and INDELs affecting the same gene; (2) CN-LOH, defined as LOH detected through B-allele frequency analysis or variant allele frequency exceeding 70% for the germline variant in the absence of copy-number alterations; and (3) SVs. Somatic tumor findings were limited to P/LP variants of diagnostic and/or prognostic relevance defined by study protocols ALLTogether for ALL and NOPHO-DBH-AML-2012 for AML^68,69^ and through an additional analysis using the ChiCaP gene panel for second-hit detection.

### Standard-of-care diagnostic tests

Standard-of-care diagnostic tests for acute leukemia were performed alongside WGTS according to ongoing study protocols (mentioned above). Standard-of-care diagnostic tests for pediatric ALL and AML in Sweden comprise various cytogenetic and molecular genetic analyses, including chromosomal G-banding analysis, fluorescence in situ hybridization (FISH) analysis, targeted analysis, gene panel sequencing, chromosomal microarray analysis (CMA) or multiplex ligation-dependent probe amplification (MLPA) according to well-established standard protocols. The analyses were performed in five Swedish university hospital laboratories in Gothenburg, Lund, Stockholm, Uppsala and Umeå.

Systematic germline predisposition testing was not conducted. Reflex germline testing was performed when the leukemia karyotype (i.e., monosomy 7/7q-, low hypodiploidy (31-39 chromosomes), iAMP21) indicated an underlying germline predisposition.

### Investigation of mosaic trisomy 21

To determine whether the same parental chromosome 21 was duplicated across tissues and malignancies in SE_AL029, we identified 24 informative single nucleotide polymorphisms (SNPs) distributed across chromosome 21. SNPs (hg19) were selected based on a biased signal on the SNP array in the skin fibroblast sample (array ratio <0.65 or >2.0), indicating allelic imbalance consistent with trisomy. Genomic positions were translated to the GRCh38 reference assembly. For the ALL sample, variant allele frequencies (VAFs) were extracted from WGS data. For the fibroblast sample, the allelic ratio was calculated from SNP array signal intensities as “alternative signal”/(“alternative signal”+“reference signal”) for the corresponding allele. Concordance of the duplicated allele was assessed across all available sample types.

### Comparison with the Swedish childhood cancer registry data

To assess the population coverage of the GMS-ChiCaP study and address potential sources of bias, we conducted a comparative analysis using aggregated data from the Swedish Childhood Cancer Registry (SCCR)^7^. The SCCR is a National Quality Registry used to evaluate treatment outcomes for children diagnosed with cancer. No individual-level registry data were analyzed. For comparison with data from the GMS-ChiCaP study, we extracted aggregated data on cancer diagnoses registered between 17 December 2021 and 30 April 2024. Patients were included in the study cohort based on the date when their sample (e.g., skin biopsy or remission blood) for germline analysis reached the Department of Clinical Genetics, not on the date of leukemia diagnosis. Therefore, the cohorts differ slightly at the beginning and end of the study period.

### Analysis of data from ChiCaP criteria form

Data collected through the ChiCaP criteria form were manually reviewed and assessed for fulfillment of ChiCaP criteria developed by Tesi et al.^26^ and detailed in Supplementary Information. Additional conditions were added specifically for familial history of hematological malignancies: FDR_SDR_blood_cancer (first- and second-degree relative with hematological malignancy regardless of age at diagnosis); FDR_blood_cancer (first-degree relative with hematological malignancy regardless of age at diagnosis); SDR_blood_cancer (second-degree relative with hematological malignancy regardless of age at diagnosis); TDR_blood_cancer (third-degree relative with hematological malignancy regardless of age at diagnosis). All the conditions used to evaluate the family history and the non-cancer medical history in the ChiCaP criteria form are available in Supplementary Information.

### Statistics and reproducibility

No statistical method was used to predetermine sample size; all eligible patients who consented were included. No data were excluded from the analyses. As this was an observational cohort study, randomization and blinding were not applicable. Data were analyzed as recorded, without imputation. Proportions are reported using the number of patients with available data for the variable in question as the denominator; denominators therefore vary between variables and are reported throughout as *n*/*N* (%) or % (*n*/*N*). Germline sequencing results were available for all 181 patients, so the denominator for the primary diagnostic yield estimate is complete. Descriptive statistics were used for demographic and clinical characteristics: categorical variables are presented as frequencies and percentages, and continuous variables as medians with interquartile ranges (IQR). No inferential hypothesis testing was performed. Binomial 95% confidence intervals for proportions were calculated using the hybrid Wilson/Brown method. All analyses and figure visualizations were performed using GraphPad Prism version 10.5.0 or 11.0.2 and can be reproduced using the Source Data and Supplementary files.

### Reporting guideline

The study is reported in accordance with the STROBE (Strengthening the Reporting of Observational Studies in Epidemiology) statement for cohort studies. The completed STROBE checklist is provided with this article.

### Reporting summary

Further information on research design is available in the Nature Portfolio Reporting Summary linked to this article.

## Supplementary information

Supplementary Information

Description of Additional Supplementary Files

Supplementary Data 1

Supplementary Data 2

Supplementary Data 3

Reporting Summary

Transparent Peer Review file

## Source data

Source data

## Data Availability

The raw and processed genomic data generated in this study constitute sensitive personal data under the GDPR (Regulation (EU) 2016/679) and are therefore available under restricted access to safeguard participant privacy, consistent with the signed informed-consent forms and the approval of the Swedish Ethical Review Authority (no. 2021-04135). Region Uppsala is the responsible research principal and data controller. Access is currently open for research within the approved study (no. 2021-04135), governed by a Joint Controller Agreement. Access to the data outside the approved study is under development. Future access outside the approved study will be subject to the GDPR (Regulation (EU) 2016/679), the terms of the signed informed consent, and the requirement of a study approved by the Swedish Ethical Review Authority; other legal agreements governing data access and processing may also apply. Requests regarding the status of access outside the approved study (no. 2021-04135) should be directed to Region Uppsala and the Principal Investigator (josefine.palle@akademiska.se). Response times may vary depending on the other obligations of the Principal Investigator. Source Data are provided with this paper. Pseudonymized personal data were obtained from the national Swedish Registry holders after ethical approval and secrecy assessment. According to Swedish laws and regulations, personal sensitive data can only be made available to researchers who fulfill legal requirements for access. Contact Professor Ann Nordgren (ann.nordgren@ki.se) for questions about data access. Source data are provided with this paper.

## Code availability

This study did not involve any custom code. All bioinformatic analyses were carried out using previously published, publicly available tools listed in the Methods section.

## References

[1] Coebergh, J. W. et al. Leukaemia incidence and survival in children and adolescents in Europe during 1978-1997. Report from the Automated Childhood Cancer Information System project. *Eur. J. Cancer* **42**, 2019–2036 (2006). 10.1016/j.ejca.2006.06.005 16919768

[2] Ward, E., DeSantis, C., Robbins, A., Kohler, B. & Jemal, A. Childhood and adolescent cancer statistics, 2014. *CA Cancer J. Clin.* **64**, 83–103 (2014). 10.3322/caac.21219 24488779

[3] Ssenyonga, N. et al. Worldwide trends in population-based survival for children, adolescents, and young adults diagnosed with leukaemia, by subtype, during 2000-14 (CONCORD-3): analysis of individual data from 258 cancer registries in 61 countries. *Lancet Child Adolesc. Health* **6**, 409–431 (2022). 10.1016/S2352-4642(22)00095-5 35468327

[4] Hjalgrim, L. L. et al. Age- and sex-specific incidence of childhood leukemia by immunophenotype in the Nordic countries. *J. Natl. Cancer Inst.* **95**, 1539–1544 (2003). 10.1093/jnci/djg064 14559876

[5] Pfister, S. M. et al. A summary of the inaugural WHO classification of pediatric tumors: transitioning from the optical into the molecular era. *Cancer Discov.* **12**, 331–355 (2022). 10.1158/2159-8290.CD-21-1094 PMC9401511 34921008

[6] de Rooij, J. D., Zwaan, C. M. & van den Heuvel-Eibrink, M. Pediatric AML: from biology to clinical management. *J. Clin. Med.* **4**, 127–149 (2015). 10.3390/jcm4010127 PMC4470244 26237023

[7] Swedish Childhood Cancer Registry Annual Report Barncancerregistret). Swedish Childhood Cancer Registry (2023).

[8] Zhang, J. et al. Germline mutations in predisposition genes in pediatric cancer. *N. Engl. J. Med.* **373**, 2336–2346 (2015). 10.1056/NEJMoa1508054 PMC4734119 26580448

[9] Byrjalsen, A. et al. Nationwide germline whole genome sequencing of 198 consecutive pediatric cancer patients reveals a high incidence of cancer prone syndromes. *PLoS Genet.* **16**, e1009231 (2020). 10.1371/journal.pgen.1009231 PMC7787686 33332384

[10] Newman, S. et al. Genomes for kids: the scope of pathogenic mutations in pediatric cancer revealed by comprehensive DNA and RNA sequencing. *Cancer Discov.* **11**, 3008–3027 (2021). 10.1158/2159-8290.CD-20-1631 PMC8783930 34301788

[11] Bakhuizen, J. J. et al. Comparison of clinical selection-based genetic testing with phenotype-agnostic extensive germline sequencing to diagnose genetic predisposition in children with cancer: a prospective diagnostic study. *Lancet Child Adolesc. Health* **8**, 751–761 (2024). 10.1016/S2352-4642(24)00144-5 39159644

[12] Bakhuizen, J. J. et al. Assessment of cancer predisposition syndromes in a national cohort of children with a neoplasm. *JAMA Netw. Open* **6**, e2254157 (2023). 10.1001/jamanetworkopen.2022.54157 PMC9898819 36735256

[13] Jeong, D. et al. Prevalence of germline predisposition gene mutations in pediatric acute myeloid leukemia: Genetic background of pediatric AML. *Leuk. Res.* **85**, 106210 (2019). 10.1016/j.leukres.2019.106210 31470354

[14] Samaraweera, S. E. et al. Childhood acute myeloid leukemia shows a high level of germline predisposition. *Blood* **138**, 2293–2298 (2021). 10.1182/blood.2021012666 34521114

[15] Chen, C. et al. Cancer germline predisposing variants and late mortality from subsequent malignant neoplasms among long-term childhood cancer survivors: a report from the St Jude Lifetime Cohort and the Childhood Cancer Survivor Study. *Lancet Oncol.* **24**, 1147–1156 (2023). 10.1016/S1470-2045(23)00403-5 PMC10712938 37797633

[16] Stoltze, U. et al. Overt and covert genetic causes of pediatric acute lymphoblastic leukemia. *Leukemia* **39**, 1031–1045 (2025). 10.1038/s41375-025-02535-4 40128563

[17] Bloom, M., Maciaszek, J. L., Clark, M. E., Pui, C. H. & Nichols, K. E. Recent advances in genetic predisposition to pediatric acute lymphoblastic leukemia. *Expert Rev. Hematol.* **13**, 55–70 (2020). 10.1080/17474086.2020.1685866 PMC10576863 31657974

[18] Kratz, C. P. Re-envisioning genetic predisposition to childhood and adolescent cancers. *Nat. Rev. Cancer* **25**, 109–128 (2025). 10.1038/s41568-024-00775-7 39627375

[19] Ripperger, T. et al. Childhood cancer predisposition syndromes-A concise review and recommendations by the Cancer Predisposition Working Group of the Society for Pediatric Oncology and Hematology. *Am. J. Med. Genet. A* **173**, 1017–1037 (2017). 10.1002/ajmg.a.38142 28168833

[20] Porter, C. C. Germ line mutations associated with leukemias. *Hematol. Am. Soc. Hematol. Educ. Program* **2016**, 302–308 (2016). 10.1182/asheducation-2016.1.302 PMC6142470 27913495

[21] Roberts, I. & Izraeli, S. Haematopoietic development and leukaemia in Down syndrome. *Br. J. Haematol.* **167**, 587–599 (2014). 10.1111/bjh.13096 25155832

[22] Wachtmeister, A. et al. Lifetime risk of solid tumors and leukemia in Down Syndrome: a population-based Swedish matched cohort study. *Br. J. Cancer* **134**, 790–798 (2026). 10.1038/s41416-025-03318-5 PMC12905194 41454185

[23] Arber, D. A. et al. International Consensus Classification of Myeloid Neoplasms and Acute Leukemias: integrating morphologic, clinical, and genomic data. *Blood* **140**, 1200–1228 (2022). 10.1182/blood.2022015850 PMC9479031 35767897

[24] Khoury, J. D. et al. The 5th edition of the World Health Organization Classification of Haematolymphoid Tumours: Myeloid and Histiocytic/Dendritic Neoplasms. *Leukemia* **36**, 1703–1719 (2022). 10.1038/s41375-022-01613-1 PMC9252913 35732831

[25] Junk, S. V. et al. A multi-dimensional approach to recognize genetic predisposition in children with acute lymphoblastic leukemia. *EJC Paediatr. Oncol.* **6**, 100320 (2025).

[26] Tesi, B. et al. Diagnostic yield and clinical impact of germline sequencing in children with CNS and extracranial solid tumors-a nationwide, prospective Swedish study. *Lancet Reg. Health Eur.* **39**, 100881 (2024). 10.1016/j.lanepe.2024.100881 PMC11129334 38803632

[27] Byrjalsen, A. et al. Novel germline TP53 variant (p.(Phe109Ile)) confers high risk of cancer. *J. Med Genet* **61**, 1023–1025 (2024). 10.1136/jmg-2024-110255 39317423

[28] Lundin, C. et al. High frequency of BTG1 deletions in acute lymphoblastic leukemia in children with down syndrome. *Genes Chromosomes Cancer* **51**, 196–206 (2012). 10.1002/gcc.20944 22072402

[29] Hanada, I. et al. Gene alterations involving the CRLF2-JAK pathway and recurrent gene deletions in Down syndrome-associated acute lymphoblastic leukemia in Japan. *Genes Chromosomes Cancer* **53**, 902–910 (2014). 10.1002/gcc.22201 25044358

[30] Buitenkamp, T. D. et al. Outcome in children with Down’s syndrome and acute lymphoblastic leukemia: role of IKZF1 deletions and CRLF2 aberrations. *Leukemia* **26**, 2204–2211 (2012). 10.1038/leu.2012.84 22441210

[31] Kosaki, R., Terashima, H., Kubota, M. & Kosaki, K. Acute myeloid leukemia-associated DNMT3A p.Arg882His mutation in a patient with Tatton-Brown-Rahman overgrowth syndrome as a constitutional mutation. *Am. J. Med Genet. A* **173**, 250–253 (2017). 10.1002/ajmg.a.37995 27991732

[32] Tatton-Brown, K. et al. The Tatton-Brown-Rahman syndrome: a clinical study of 55 individuals with de novo constitutive DNMT3A variants. *Wellcome Open Res.* **3**, 46 (2018). 10.12688/wellcomeopenres.14430.1 PMC5964628 29900417

[33] Ferris, M. A. et al. DNMT3A overgrowth syndrome is associated with the development of hematopoietic malignancies in children and young adults. *Blood* **139**, 461–464 (2022). 10.1182/blood.2021014052 PMC8777205 34788385

[34] Tovy, A. et al. Perturbed hematopoiesis in individuals with germline DNMT3A overgrowth Tatton-Brown-Rahman syndrome. *Haematologica* **107**, 887–898 (2022). 10.3324/haematol.2021.278990 PMC8968878 34092059

[35] Fiala, E. M. et al. Prospective pan-cancer germline testing using MSK-IMPACT informs clinical translation in 751 patients with pediatric solid tumors. *Nat. Cancer* **2**, 357–365 (2021). 10.1038/s43018-021-00172-1 PMC8294573 34308366

[36] Grobner, S. N. et al. The landscape of genomic alterations across childhood cancers. *Nature* **555**, 321–327 (2018). 10.1038/nature25480 29489754

[37] Wagener, R. et al. Comprehensive germline-genomic and clinical profiling in 160 unselected children and adolescents with cancer. *Eur. J. Hum. Genet.* **29**, 1301–1311 (2021). 10.1038/s41431-021-00878-x PMC8385053 33840814

[38] Stoltze, U. K. et al. Childhood cancer predisposition and evolutionary constraints: novel lessons from germline genomes from 1,127 children with cancer. *Clin. Cancer Res*. **31**, 4495–4509 (2025). 10.1158/1078-0432.CCR-25-0153 40815213

[39] Winter, G. et al. Clinical and genetic characteristics of children with acute lymphoblastic leukemia and Li-Fraumeni syndrome. *Leukemia* **35**, 1475–1479 (2021). 10.1038/s41375-021-01163-y PMC8102191 33580201

[40] Qian, M. et al. TP53 Germline Variations Influence the Predisposition and Prognosis of B-Cell Acute Lymphoblastic Leukemia in Children. *J. Clin. Oncol.* **36**, 591–599 (2018). 10.1200/JCO.2017.75.5215 PMC5815403 29300620

[41] Pabst, T., Eyholzer, M., Haefliger, S., Schardt, J. & Mueller, B. U. Somatic CEBPA mutations are a frequent second event in families with germline CEBPA mutations and familial acute myeloid leukemia. *J. Clin. Oncol.* **26**, 5088–5093 (2008). 10.1200/JCO.2008.16.5563 18768433

[42] Taskesen, E. et al. Prognostic impact, concurrent genetic mutations, and gene expression features of AML with CEBPA mutations in a cohort of 1182 cytogenetically normal AML patients: further evidence for CEBPA double mutant AML as a distinctive disease entity. *Blood* **117**, 2469–2475 (2011). 10.1182/blood-2010-09-307280 21177436

[43] Ng, A. P. et al. Reporting bone marrow biopsies for myelodysplastic neoplasms and acute myeloid leukaemia incorporating WHO 5th edition and ICC 2022 classification systems: ALLG/RCPA joint committee consensus recommendations. *Pathology* **56**, 459–467 (2024). 10.1016/j.pathol.2024.02.002 38580613

[44] Baliakas, P. et al. Nordic Guidelines for Germline Predisposition to Myeloid Neoplasms in Adults: recommendations for genetic diagnosis, clinical management and follow-up. *Hemasphere* **3**, e321 (2019). 10.1097/HS9.0000000000000321 PMC6924562 31976490

[45] Dohner, H. et al. Diagnosis and management of AML in adults: 2022 recommendations from an international expert panel on behalf of the ELN. *Blood* **140**, 1345–1377 (2022). 10.1182/blood.2022016867 35797463

[46] Ripperger, T. & Schlegelberger, B. Acute lymphoblastic leukemia and lymphoma in the context of constitutional mismatch repair deficiency syndrome. *Eur. J. Med. Genet.* **59**, 133–142 (2016). 10.1016/j.ejmg.2015.12.014 26743104

[47] McReynolds, L. J., Biswas, K., Giri, N., Sharan, S. K. & Alter, B. P. Genotype-cancer association in patients with Fanconi anemia due to pathogenic variants in FANCD1 (BRCA2) or FANCN (PALB2). *Cancer Genet.* **258-259**, 101–109 (2021). 10.1016/j.cancergen.2021.10.001 PMC8628873 34687993

[48] Onder, G. et al. Genetic heterogeneity in childhood leukemia/lymphoma: a Turkish cohort with strong predisposition. *Front. Genet.* **16**, 1624306 (2025). 10.3389/fgene.2025.1624306 PMC12454056 40995433

[49] McGee, R. B. et al. Pathogenic variants in adult-onset cancer predisposition genes in pediatric cancer: prevalence and impact on tumor molecular features and clinical management. *Clin. Cancer Res.* **29**, 1243–1251 (2023). 10.1158/1078-0432.CCR-22-2482 PMC10642481 36693186

[50] Dubard Gault, M. et al. Germline SDHA mutations in children and adults with cancer. *Cold Spring Harb. Mol. Case Stud.* **4**, a002584 (2018). 10.1101/mcs.a002584 PMC6071569 30068732

[51] Casey, R. T. et al. SDHA related tumorigenesis: a new case series and literature review for variant interpretation and pathogenicity. *Mol. Genet. Genom. Med.* **5**, 237–250 (2017). 10.1002/mgg3.279 PMC5441402 28546994

[52] Weijers, D. D. et al. Molecular analysis of cancer genomes in children with Lynch syndrome: Exploring causal associations. *Int J. Cancer* **154**, 1455–1463 (2024). 10.1002/ijc.34832 38175816

[53] Holmfeldt, L. et al. The genomic landscape of hypodiploid acute lymphoblastic leukemia. *Nat. Genet* **45**, 242–252 (2013). 10.1038/ng.2532 PMC3919793 23334668

[54] Tawana, K. et al. Disease evolution and outcomes in familial AML with germline CEBPA mutations. *Blood* **126**, 1214–1223 (2015). 10.1182/blood-2015-05-647172 26162409

[55] Porter, C. C. et al. Recommendations for surveillance for children with leukemia-predisposing conditions. *Clin. Cancer Res.* **23**, e14–e22 (2017). 10.1158/1078-0432.CCR-17-0428 28572263

[56] Kratz, C. P. et al. Cancer screening recommendations for individuals with Li-Fraumeni syndrome. *Clin. Cancer Res* **23**, e38–e45 (2017). 10.1158/1078-0432.CCR-17-0408 28572266

[57] Ruchel, N. et al. In utero development and immunosurveillance of B cell acute lymphoblastic leukemia. *Curr. Treat. Options Oncol.* **23**, 543–561 (2022). 10.1007/s11864-022-00963-3 PMC8924576 35294722

[58] Cobaleda, C., Vicente-Duenas, C., Nichols, K. E. & Sanchez-Garcia, I. Childhood B cell leukemia: Intercepting the paths to progression. *Bioessays* **46**, e2400033 (2024). 10.1002/bies.202400033 PMC11864036 39058907

[59] DeRoin, L. et al. Feasibility and limitations of cultured skin fibroblasts for germline genetic testing in hematologic disorders. *Hum. Mutat.* **43**, 950–962 (2022). 10.1002/humu.24374 PMC9177640 35419889

[60] Poplin, R. et al. A universal SNP and small-indel variant caller using deep neural networks. *Nat. Biotechnol.* **36**, 983–987 (2018). 10.1038/nbt.4235 30247488

[61] Byrjalsen, A. et al. Selection criteria for assembling a pediatric cancer predisposition syndrome gene panel. *Fam. Cancer* **20**, 279–287 (2021). 10.1007/s10689-021-00254-0 PMC8484084 34061292

[62] Richards, S. et al. Standards and guidelines for the interpretation of sequence variants: a joint consensus recommendation of the American College of Medical Genetics and Genomics and the Association for Molecular Pathology. *Genet Med.* **17**, 405–424 (2015). 10.1038/gim.2015.30 PMC4544753 25741868

[63] Fortuno, C. et al. Specifications of the ACMG/AMP variant interpretation guidelines for germline TP53 variants. *Hum. Mutat.* **42**, 223–236 (2021). 10.1002/humu.24152 PMC8374922 33300245

[64] Hamosh, A., Amberger, J. S., Bocchini, C., Scott, A. F. & Rasmussen, S. A. Online Mendelian Inheritance in Man (OMIM(R)): Victor McKusick’s magnum opus. *Am. J. Med. Genet. A* **185**, 3259–3265 (2021). 10.1002/ajmg.a.62407 PMC8596664 34169650

[65] Stranneheim, H. et al. Integration of whole genome sequencing into a healthcare setting: high diagnostic rates across multiple clinical entities in 3219 rare disease patients. *Genome Med.* **13**, 40 (2021). 10.1186/s13073-021-00855-5 PMC7968334 33726816

[66] Malmgren, H. et al. Diagnostic yield of 1000 trio analyses with exome and genome sequencing in a clinical setting. *Front. Genet.* **16**, 1580879 (2025). 10.3389/fgene.2025.1580879 PMC12226303 40620702

[67] Lindstrand, A. et al. The genomic medicine center Karolinska 10-year report on genome sequencing for rare diseases and a strategy for stepwise clinical implementation. *Genome Med.* **18**, 30 (2026). 10.1186/s13073-026-01611-3 PMC13034595 41913253

[68] Thangavelu, T. et al. Micro-costing of genetic diagnostics in acute leukemia in Sweden: from standard-of-care to whole-genome sequencing. *J. Med. Econ.* **27**, 1053–1060 (2024). 10.1080/13696998.2024.2387515 39101813

[69] Rezayee, F. et al. Feasibility to use whole-genome sequencing as a sole diagnostic method to detect genomic aberrations in pediatric B-cell acute lymphoblastic leukemia. *Front. Oncol.* **13**, 1217712 (2023). 10.3389/fonc.2023.1217712 PMC10470829 37664045

