# Supplementary material for: Diagnostic yield of cancer predisposition in a nationwide prospective childhood acute leukemia cohort
Source: Nat Commun. 2026 Sep 28;17:10280. doi: 10.1038/s41467-026-78170-z (PMC13619629; doi:10.1038/s41467-026-78170-z)
Supplement: Supplementary file 1 [file 41467_2026_78170_MOESM1_ESM.pdf]

Supplementary Methods

Germline Whole Genome Sequencing

Germline whole genome sequencing (gWGS) was performed on1 µg DNA extracted from skin fibroblasts obtained by skin biopsy. Libraries were sequenced in paired-end 150-bp mode on flow cells using the NovaSeq 6000 or NovaSeq X instruments (Illumina).

gWGS data were analyzed using locally established rare disease (germline) pipelines of the four participating clinical genetics departments (detailed below). At all centres, analysis was restricted to a Childhood Cancer Predisposition (ChiCaP) gene list encompassing 189 genes (Supplementary Data 1), and variants were classified according to the guidelines of the American College of Medical Genetics and Genomics (ACMG)[1].

Karolinska University Hospital, Department of Clinical Genetics and Genomics (samples from Stockholm and Linköping): SNVs, indels and structural variants (SVs) were called with the Mutation Identification Pipeline (MIP, v9.1.3 or later; https://github.com/Clinical-Genomics/MIP), the standard-of-care rare-germline-variant pipeline at the Genomic Medicine Center Karolinska[2], which annotates and ranks variants in a VCF file. Ranked VCFs were uploaded to the in-house open-source visualisation tool Scout (v4.36 or later; https://github.com/Clinical-Genomics/scout), where variants with a gnomAD popmax minor allele frequency >0.01 and variants outside exonic and splice regions were filtered out prior to evaluation and interpretation. Reference genome: GRCh37 (hg19).

Sahlgrenska University Hospital, Gothenburg, Department of Clinical Genetics and Genomics (samples from Gothenburg). SNVs and small indels were called with Sentieon DNAscope (v201911 and v202112)[3] and copy number variants (CNVs) with Canvas (v1.40.0.1613, Illumina)[4]. SNVs and CNVs were filtered in Alissa Interpret (v5.3.3, Agilent), retaining (i) variants annotated as pathogenic or likely pathogenic in HGMD Professional and/or ClinVar, and (ii) non-synonymous and/or putative splice-site variants with a frequency below 5% in population databases (gnomAD, 1000 Genomes, ExAC). Retained variants were interpreted in Alamut Visual Plus (Sophia Genetics). Reference genome: GRCh38 (hg38).

Region Skåne, Lund, Department of Clinical Genetics, Pathology and Molecular Diagnostics (Samples from Lund). Analysis used the diagnostic rare-disease pipeline in clinical use at the centre (https://github.com/SMD-Bioinformatics-Lund/nextflow_wgs). SNVs and indels were called according to the Sentieon DNAscope (v202112)[3] best-practice workflow, with the callable region defined by a BED file covering coding sequence ±20 bp and non-coding positions then classified as pathogenic or likely pathogenic in ClinVar; calls were annotated with VEP[5], vcfanno, CADD, ClinVar and Genmod (https://github.com/moonso/genmod). SVs were called with three callers (GATK[6], TIDDIT[7] and Manta[8]); calls supported by more than one caller with ≥70% overlap were merged using in-house software and annotated with VEP[5], AnnotSV[9], an in-house Perl pre-score script, Genmod and an in-house compound finder. All variants (SNVs, indels, SVs) were scored and ranked based on these annotations, and the resulting VCFs were uploaded to Scout for clinical interpretation and documentation. Panel variants exceeding a predefined rank-score cut-off were assessed manually in Scout, supported by Alamut Visual Plus, IGV[10] and the locally developed visualization tool Gens (https://github.com/SMD-Bioinformatics-Lund/gens). Reference genome: GRCh38 (hg38).

Uppsala University Hospital, Department of Clinical Genetics (Samples from Uppsala and Umeå). SNVs, indels and CNVs were called with the Poirot RD WGS rare-disease pipeline (v0.8.0; https://github.com/clinical-genomics-uppsala/poirot_rd_wgs) and filtered and interpreted in Alissa Interpret (v5.4.2, Agilent). Reference genome: GRCh38 (hg38).

Tumor Sequencing and Data Analysis

Tumour DNA was extracted from bone marrow samples and analyzed by whole-genome sequencing (WGS) to a target coverage of ~90×. Libraries were prepared from 1 µg DNA using the TruSeq DNA PCR-free protocol (Illumina, San Diego, CA, USA) with a 350-bp insert size. When DNA was limiting, 100 ng input was used with the TruSeq DNA Nano protocol (Illumina). Libraries were sequenced in paired-end 150-bp mode on flow cells using the NovaSeq 6000 or NovaSeq X instruments (Illumina).

Tumour-normal pairs were analyzed at four clinical genetics units, each using a locally validated bioinformatic pipeline, as described below.

Karolinska University Hospital, Department of Clinical Genetics and Genomics (samples from Stockholm and Linköping): Samples were analyzed with BALSAMIC (Bioinformatic Analysis pipeline for Somatic Mutations in Cancer, v7.2.2 or higher; https://balsamic.readthedocs.io). Reads were aligned to hg19 with Sentieon bwa-mem. Somatic SNVs and indels were called with TNscope (Sentieon), copy-number alterations (CNAs) with ascatngs, and structural variants (SVs) with Manta[8]. Variants were annotated with the Ensembl Variant Effect Predictor (VEP)[5] and retained if total read depth was ≥10, alternative allele depth ≥3, and variant allele frequency (VAF) ≥0.05 and <1. Filtered VCFs were uploaded to SCOUT (https://github.com/Clinical-Genomics/scout) for inspection and interpretation.

Ploidy changes and CNAs were visualized with vcf2cytosure v0.7.1, included in BALSAMIC, which converts variant-calling output to the ".cgh" format used by CytoSure™ Interpret Software (Oxford Gene Technologies, Oxford, UK), originally developed for oligonucleotide microarray data. Output files were binned and 20 bins pooled per probe. Coverage along the genome was expressed as the log2 ratio of each bin relative to all bins, with the sample mean coverage set to 0 and interpreted as the diploid state (n = 2); ratios above and below this line indicate gains and losses, respectively. Because the threshold is derived from average genome-wide coverage, it was adjusted manually in samples with multiple tri-/tetrasomies or monosomies. Allele-specific copy-number analysis[11] (ASCAT, also included in BALSAMIC) was used to visualize aneuploidies and copy-number-neutral loss of heterozygosity (CN-LOH); the latter requires a paired germline sample.

Sahlgrenska University Hospital, Gothenburg, Department of Clinical Genetics and Genomics (samples from Gothenburg).

Samples from Gothenburg were analysed with the in-house pipeline "WGS Somatic". Reads were aligned to hg38 (GRCh38). Somatic SNVs and indels were called with TNscope (Sentieon Inc, Mountain View, CA, USA)[3], supplying the matched normal to remove germline variants, and likely artefacts were removed using Sentieon machine-learning models (Sentieon v201911). CNAs were identified with Canvas v1.40.0.1613 (Illumina)[4] and SVs and larger indels with the Manta Structural Variant Caller (MantaSV v1.6.0, Illumina)[8].

Somatic SNVs and indels were filtered in QIAGEN Clinical Insight (QCI) Interpret v8.1.20210827 (https://digitalinsights.qiagen.com), retaining only non-synonymous and potential splice-site variants (±10 bp from exons) with total read depth >10 and VAF ≥0.10. Variants present at >1% allele frequency in gnomAD[12] v2.1.1 (https://gnomad.broadinstitute.org/), 1000 Genomes or the Exome Aggregation Consortium (ExAC; Cambridge, MA, USA; http://exac.broadinstitute.org) were excluded unless already established as pathogenic. SVs were retained only when supported by both spanning paired reads and spanning split reads, with ≥3 supporting reads in the tumor and no more than 1 supporting read in the matched normal. Remaining variants were inspected manually in the Integrative Genomics Viewer (IGV)[10], and reported according to their diagnostic, prognostic and therapeutic relevance.

Region Skåne, Lund, Department of Clinical Genetics, Pathology and Molecular Diagnostics (Samples from Lund). Tumour–normal pairs were analysed with an in-house Nextflow[13]-based pipeline(https://github.com/SMD-Bioinformatics-Lund/nextflow_tumwgs). Reads were aligned to hg38 using Sentieon distributed bwa-mem (v202112.05). Somatic SNVs and indels were called with freebayes and VarDict[14], filtered against a customized in-house panel of normals (PON) and annotated with VEP[5] (ensembl-vep release 98.0). CNAs were called with the GATK copy-number variant module and filtered against the same PON. SVs, including gene fusions, were called with MantaSV[8] v1.6.0 and annotated with SnpEff[15].

SNVs and indels were retained with total read depth >10, alternative allele read depth >7 and VAF >0.1. All somatic variants (SNVs, indels, CNAs and SVs/fusions) were annotated and compared with public databases in the in-house software Coyote(https://github.com/SMD-Bioinformatics-Lund/coyote3), and inspected manually in IGV or the locally developed visualization tool, Gens (https://github.com/SMD-Bioinformatics-Lund/gens). False positives were discarded and remaining variants classified according to clinical impact.

Uppsala University Hospital, Department of Clinical Genetics (Samples from Uppsala and Umeå). Tumour-normal pairs were processed with the Snakemake-based workflow fluffy_hematology_wgs (https://github.com/clinical-genomics-uppsala/fluffy_hematology_wgs). Raw reads were assessed with FastQC v0.11.9 (http://www.bioinformatics.babraham.ac.uk/projects/fastqc/) and adapter-trimmed and quality-filtered with fastp v0.20.1. Reads were aligned to GRCh38 with BWA-MEM as implemented in the NVIDIA Parabricks toolkit. Quality control comprised mapping statistics (samtools stats v1.15), coverage and depth (mosdepth v0.3.2), duplicate rates and insert-size distributions (Picard v2.25.0; http://broadinstitute.github.io/picard) and sex concordance (Peddy v0.4.8); metrics were aggregated with MultiQC v1.25.1-v1.25.13.

Somatic SNVs and indels were called with GATK Mutect2 (Parabricks v4.1.1-1), complemented by Pindel v0.2.5b9 for indel detection in targeted regions. CNAs and aneuploidy were inferred with CNVkit v0.9.9 and GATK GermlineCNVCaller (GATK v4.2.2.0), using sex-specific panels of normals to improve resolution. SVs, including larger indels, duplications and translocations, were called with Manta[8] v1.6.0 and merged and annotated across samples with SVDB v2.6.0 (https://github.com/J35P312/SVDB). Variants were normalized with VT (2015-11-10), annotated with SnpEff[15] v5.0 and VEP[5] v111.0, filtered, and interpreted and classified in QCI Interpret (QIAGEN).

A curated panel used for clinical prioritization of somatic findings at all centers comprised[16]: (i) all aberrations mandatory to investigate according to the current ALL treatment protocol[17]; (ii)

the genes and genomic regions included in the UKALL-CNA classifier and IKZF1+ profiles[18, 19]; and (iii) key genes defining genetic subtypes with potential diagnostic or therapeutic implications among B-other ALL[20, 21, 22, 23, 24, 25, 26, 27, 28, 29, 30, 31] or included in the WHO 2022 classification[32] and the International Consensus Classification of hematological malignancies[33].

ChiCaP Criteria Form

At inclusion, information on family history of cancer, child's medical history of malignancy, clinical features suggestive of ChiCaP, and malignancy type was collected through the ChiCaP criteria form which was developed for all types of cancer and hematological malignancies. This form was developed by Tesi et al.[34]. Translation of the ChiCaP criteria form from Swedish to English was done using DeepL (https://www.deepl.com/en/translator), and is provided below. The form was built upon existing clinical screening tools such as Jongmans criteria, MIPOGG, etc[35, 36, 37, 38]. Focus was given to clinical features that could be assessed at diagnosis. The form collects information pertinent to four domains: 1) family history of cancer and/or parental consanguinity; 2) cancer history in the child in terms of multiple primary malignancies and/or bilateral tumors; 3) "tip-to-toe" review of non-cancer related features; 4) specific tumor diagnosis with higher prior likelihood of underlying predisposition. A box for comments in free text was available at the end of the form for the physicians to provide further information. Domains 1, 2 and 4 were designed after a comparison of existing screening tools to create a combined list[35, 36, 37, 38]. Therefore, by design, the ChiCaP criteria form was expected to turn out positive in a larger proportion of children compared to other tools. The ChiCaP criteria form was filled out by physicians specialized in pediatric, pediatric oncology or medical oncology shortly after the collection of informed consent from parents or legal guardians.

1. **Family history:** *Ask about cancer in at least three generations i.e. parents, siblings, parents' siblings, cousins, grandparents, grandparents' siblings, grandparents, grandparents' siblings, possibly other relatives. Mark with a cross.*

☐ The child's parents are related.

☐ At least one relative with cancer before age 18.

☐ A parent or sibling/half-sibling affected by cancer before the age of 50.

☐ At least 2 relatives on the same side of the family with cancer before the age of 50.

☐ At least one relative with multiple cancers.

☐ At least one relative with the same type of cancer/tumor as the child.

Indicate which relative, if on mother's/father's side and type of cancer

☐ None of the above

2. **Child's medical history of malignancy/tumor:** *Mark with a cross.*

☐ > 1 primary malignancy/tumor (synchronous - within 6 months or metachronous - after 6 months) regardless of diagnosis.

☐ Bilateral or multifocal primary malignancy/tumor regardless of diagnosis

☐ None of the above

3. **Congenital malformations or other specific symptoms**. *Mark with a cross.*

Category		
**Cognition / Behavior**	☐ Age-appropriate, without remarks<br>☐ Intellectual disability<br>☐ Development delay<br>☐ Autism spectrum disorder	☐ Other:
**Abnormal growth* (*If abnormal, attach growth charts)**	☐ Growth and proportions, etc.<br>☐ Small for gestational age (SGA) height or weight < -2 SDS for gestational length<br>☐ Short-grown (< -2.5 SD)<br>☐ Tall (> 2.5 SD)<br>☐ Macrocephaly (> 2 SD)<br>☐ Microcephaly (< -2 SD)<br>☐ Obesity (Iso-BMI > 30)<br>☐ Abnormal body proportions	☐ Wide neck<br>☐ hemihypertrophy<br>☐ Macroglossia<br>☐ Other asymmetry<br>☐ Other anomaly:
**Facial dysmorphology**	☐ None<br>☐ Clear suspicion<br>☐ Some suspicion	☐ In case of suspicion, try to describe what is involved:
**Congenital malformations/ Other abnormalities**	☐ No malformations/other abnormalities<br>☐ Congenital severe visual impairment<br>☐ Congenital severe hearing loss<br>☐ Cleft lip and palate<br>☐ Internal malformations (heart, vessels, CNS, kidneys, GI, airways)<br>☐ Skeletal dysplasia/skeletal malformation<br>☐ Abnormal shape of the chest<br>☐ Radial anomalies including thumbs<br>☐ Other limb abnormalities<br>☐ Craniosynostosis<br>☐ Abnormal urogenitalia	☐ Strabismus<br>☐ Glaucoma/Cataract<br>☐ Aniridia<br>☐ White pupil reflex<br>☐ Other eye malformations<br>☐ Cysts in internal organs<br>☐ Other anomaly:

**Hair and skin**	☐ Unremarkable<br>☐ >2 café-au-lait spots<br>☐ Hypo/hyper-pigmentation<br>☐ Freckles on an unusual area (e.g. lips, inside the mouth, on the glans, on fingers and toes, axillae or groin).<br>☐ Teleangiectasia<br>☐ Other vascular skin changes<br>☐ Multiple benign skin tumours<br>☐ Hairy skin plaques<br>☐ Hypersensitivity to sunlight	☐ Brittle / grey hair<br>☐ Abnormal hairiness<br>☐ Brittle nails<br>☐ Leukoplakia<br>☐ Pit / fold in ear helix<br>☐ Supernumerary nipple<br>☐ Other anomaly:
**Neurological/ Muscular/ Connective tissue abnormalities (not due to cancer)**	☐ No neurological / muscular / connective tissue abnormalities<br>☐ Ataxia<br>☐ Epilepsy<br>☐ Spasticity<br>☐ Muscular hypotonia	☐ Dystonia<br>☐ hypermobility<br>☐ Vascular emergencies<br>☐ Hernia<br>☐ Other anomaly:
**Hematological abnormalities (not due to the cancer)**	☐ No hematological abnormalities<br>☐ Pancytopenia<br>☐ Anemia<br>☐ Thrombocytopenia<br>☐ Neutropenia	☐ Leukopenia<br>☐ Macrocytic erythrocytes<br>☐ Other anomaly:
**Immune system**	☐ No immunological defects<br>☐ Investigated for immune deficiency<br>☐ Hypogammaglobulinemia<br>☐ Autoimmunity	☐ Inflammation<br>☐ Other anomaly:
**Endocrine abnormalities**	☐ No endocrine abnormalities<br>☐ Primary hyperparathyroidism<br>☐ Early/late puberty<br>☐ Hypogonadism<br>☐ Prolactinoma<br>☐ Gigantism / acromegaly	☐ Cushing's syndrome<br>☐ Goiter<br>☐ Other anomaly:

☐ none of the above congenital malformations or other specific symptoms

1. **<u>Please indicate below the child's confirmed/suspected cancer/tumor *diagnosis:*</u>**

☐ Pathology report not ready - suspected diagnosis:

____

☐ The child has one of the following diagnoses. *Check the box if the diagnosis has been established.*

Any of the following malignancies/tumors of the nervous system:	☐ Atypical teratoid rhabdoid tumor<br>☐ Dysplastic cerebellar gangliocytoma<br>☐ Endolymphatic sac tumors<br>☐ Ependymoma<br>☐ Hemangioblastoma<br>☐ Pituitary gland: adenoma/tumor/blastoma<br>☐ Malignant/benign peripheral nerve sheath tumor<br>☐ Medulloblastoma<br>☐ Medulloepithelioma<br>☐ Meningioma<br>☐ Optic glioma<br>☐ Pineoblastoma<br>☐ Choroidal plexus carcinoma<br>☐ Retinoblastoma<br>☐ Schwannomas (vestibular schwannomas or schwannomatosis)<br>☐ Subependymal giant cell astrocytoma	
Any of the following solid tumors	☐ Angiomyolipoma (kidneys and/or liver)<br>☐ Adrenalcortical carcinoma<br>☐ Cystic nephroma<br>☐ Fetal rhabdomyoma<br>☐ Gastrointestinal stromal tumor<br>☐ Gonadoblastoma<br>☐ Hepatoblastoma<br>☐ Infantile myofibromatosis<br>☐ Chondromesenchymal harmatoma<br>☐ Gonadal stromal cell tumor with annular tubules<br>☐ Myxoma<br>☐ Neuroblastoma<br>☐ Neuroendocrine tumors<br>☐ Paraganglioma/Pheochromocytoma<br>☐ Parathyroid cancer/adenoma<br>☐ Pleuropulmonary blastoma<br>☐ Rhabdoid tumor (RT): Atypical teratoid RT or renal / extrarenal malignant RT<br>☐ Rhabdomyosarcoma<br>☐ Sertoli-Leydig cell tumor<br>☐ Small cell ovarian cancer of hypercalcemic type<br>☐ Wilms' tumor	
Any cancer usually seen in adults:	☐ Basalioma<br>☐ Colorectal cancer	☐ Ovarian cancer<br>☐ Squamous cell

	☐ Liver cancer<br>☐ Malignant melanoma<br>☐ Kidney cancer	carcinoma<br>☐ Thyroid cancer<br>☐ Other tumor:
Any other benign tumor that may indicate cancer predisposition syndrome	☐ Angiofibroma<br>☐ Fibrofolliculoma<br>☐ Keratocystic odontogenic tumor<br>☐ Myofibromatosis	☐ Neurofibroma<br>☐ Trichodiscoma<br>☐ Other tumor:

☐ None of the above confirmed / suspected cancer / tumor diagnosis

Assessment of family history of cancer was facilitated through a questionnaire that could be filled out by the child's parents. This questionnaire was optional. The following questions were asked:

1) Is there any relationship between the two of you? (For example, are you cousins, second cousins or do you know that you are distantly related to each other?) If yes - please describe how you are related;
2) Have any of you had cancer? If yes - please indicate which of you has had cancer and what type of cancer and at what age;
3) Do you have other children (from the same or different relationship) who have had cancer in childhood or adulthood? If yes - please specify who and if possible what type of cancer and at what age;
4) Is there anyone else in your family who got cancer before the age of 18? If yes - please specify who and if possible what type of cancer and at what age;
5) Is there anyone among your parents and siblings who got cancer before the age of 50? If yes - please describe on whose side of the family, how that person is related to you, and, if possible, what type of cancer and at what age;
6) Are there any other relatives with multiple cancers? If yes, please specify who and, if possible, what type of cancer and at what age.

Analysis of Data from ChiCaP Criteria Form

Data collected through the ChiCaP criteria form were manually reviewed and assessed for fulfillment of ChiCaP criteria. The family history criterion was considered fulfilled if any of the following conditions were met:

- Parent_cancer_before_50yo: parent with any type of cancer before age 50 years;
- 2_rel_cancer_before_50yo: 2 relatives with any type of cancer on same side before age 50 years;
- rel_cancer_before_18yo: relative with any type cancer before age 18 years;
- rel_same_organ: relative with cancer affecting the same organ, regardless of age at diagnosis;
- rel_same_cancer: relative with same type of cancer, regardless of age at diagnosis;
- rel_2_cancer_before_60yo: relative with 2 primary malignancies before age 60 (excluding nonmelanoma skin cancer);

- halfsib_cancer_before_50yo: half sibling with any type of cancer before age 50;
- Consanguinity: consanguineous parents (any level).

The criterion for child's medical history of malignancy was considered fulfilled only in children with multiple primary tumors, i.e. included at diagnosis of second/third primary tumor.

The criterion for other symptoms was considered fulfilled if any features were reported that could not be explained by the underlying CNS or extracranial solid tumor. For further analysis, clinical features were grouped as follows (examples of reported clinical features are provided for each category):

- Cutaneous: any reported skin/hair feature – e.g. stork bite, hemangioma, telangiectasia, hypo/hyperpigmentation, cafe-au-lait spots;
- Cognition_behaviour: any reported anomaly of cognition/behaviour – e.g. ADD, ADHD, autism, autism spectrum disorder, intellectual disability, developmental delay, ongoing neuropsychiatric assessment;
- Growth: any reported growth abnormality – e.g. length > 2SD, obesity, short stature, microcephaly;
- Inflammation_immune: any reported immunological defect – e.g. immunodeficiency, Periodic Fever Aphthous Stomatitis Pharyngitis Adenitis, systemic juvenile arthritis;
- Malformation: any reported congenital malformation – e.g. kidney cysts, kidney malformation, umbilical hernia, single testicle, heart malformation, polydactyly;
- Neuro_muscular: any reported neuromuscular symptoms – e.g. hypotonia, epilepsy, tics, motor developmental delay;
- Endocrine: any reported hormonal defect – e.g. diabetes mellitus type 1, abnormal gonadotropine levels;
- Dysmorphism: facial dysmorphism;
- Skeletal_abnormality: any reported skeletal abnormality
- Other: any more rarely reported abnormality, occurring ≤2 in the whole cohort – e.g. hearing loss, blindness, blood abnormality

The child was classified as having ≥2 features in case of multiple congenital malformations, multiple type of skin changes (eg. pigmentation and vascular), or symptoms/signs affecting >1 system, e.g. cutaneous and growth. The child was classified as having intellectual disability (ID) or developmental delay (DD) only when the respective boxes on the form were ticked. The criterion for malignancy type was considered fulfilled in all children with a final diagnosis listed under that criterion.

Supplementary Tables

Supplementary Table 1. Comparison of ChiCaP prevalence in different studies

	**Zhang *et al.*, 2015**[39] (excl. Down syndrome)	**Byrjalsen *et al.*, 2020**[40] (incl. Down syndrome)	**Newman *et al.*, 2021**[41] (excl. Down syndrome)	**Bakhuizen *et al.*, 2023**[42] (incl. Down syndrome)	**Bakhuizen *et al.*, 2024**[43] (Supplemental table 8 of the study)	**Bakhuizen *et al.*, 2024**[43] (Table 2 of the study) Newly sequenced cohort	**This study** (incl. Down syndrome)
Total number of leukemia patients in the study (n)	588	74	126	216	280	184	181
P/LP in all CPS genes (child+adult, genes for all cancer types) *n (percentage)*	26 (4.4%)	11 (14.9%)	16 (12.7%)	12 (5.5%)	20 (7.1%)	7 (3.8%)	9 (5.0%)
P/LP in leukemia-associated CPS genes in children *n (percentage)*	16 (2.7%)	6 (8.1%)	9 (7.1%)	11(5%)	NA	1 (0.5%)	6 (3.3%)
P/LP in leukemia-associated CPS genes in children (excl. Down syndrome) *n (percentage)*	16 (2.7%)	3 (4%)	4 (3.2%)	2 (0.9%)	NA	1 (0.5%)	3 (1.7%)

REFERENCES

1. Richards S, *et al.* Standards and guidelines for the interpretation of sequence variants: a joint consensus recommendation of the American College of Medical Genetics and Genomics and the Association for Molecular Pathology. *Genet Med* **17**, 405–424 (2015).

2. Stranneheim H, *et al.* Integration of whole genome sequencing into a healthcare setting: high diagnostic rates across multiple clinical entities in 3219 rare disease patients. *Genome Medicine* **13**, 40 (2021).

3. Aldana R, Freed D. Data Processing and Germline Variant Calling with the Sentieon Pipeline. *Methods Mol Biol* **2493**, 1–19 (2022).

4. Roller E, Ivakhno S, Lee S, Royce T, Tanner S. Canvas: versatile and scalable detection of copy number variants. *Bioinformatics* **32**, 2375–2377 (2016).

5. McLaren W, *et al.* The Ensembl Variant Effect Predictor. *Genome Biol* **17**, 122 (2016).

6. McKenna A, *et al.* The Genome Analysis Toolkit: a MapReduce framework for analyzing next-generation DNA sequencing data. *Genome Res* **20**, 1297–1303 (2010).

7. Eisfeldt J, Vezzi F, Olason P, Nilsson D, Lindstrand A. TIDDIT, an efficient and comprehensive structural variant caller for massive parallel sequencing data. *F1000Res* **6**, 664 (2017).

8. Chen X, *et al.* Manta: rapid detection of structural variants and indels for germline and cancer sequencing applications. *Bioinformatics* **32**, 1220–1222 (2016).

9. Geoffroy V, *et al.* AnnotSV: an integrated tool for structural variations annotation. *Bioinformatics* **34**, 3572–3574 (2018).

10. Robinson JT, *et al.* Integrative genomics viewer. *Nat Biotechnol* **29**, 24–26 (2011).

11. Van Loo P, *et al.* Allele-specific copy number analysis of tumors. *Proc Natl Acad Sci U S A* **107**, 16910–16915 (2010).

12. Karczewski KJ, *et al.* The mutational constraint spectrum quantified from variation in 141,456 humans. *Nature* **581**, 434–443 (2020).

13. Di Tommaso P, Chatzou M, Floden EW, Barja PP, Palumbo E, Notredame C. Nextflow enables reproducible computational workflows. *Nat Biotechnol* **35**, 316–319 (2017).

14. Lai Z*, et al.* VarDict: a novel and versatile variant caller for next-generation sequencing in cancer research. *Nucleic Acids Res* **44**, e108 (2016).

15. Cingolani P*, et al.* A program for annotating and predicting the effects of single nucleotide polymorphisms, SnpEff: SNPs in the genome of Drosophila melanogaster strain w1118; iso-2; iso-3. *Fly (Austin)* **6**, 80–92 (2012).

16. Rezayee F*, et al.* Feasibility to use whole-genome sequencing as a sole diagnostic method to detect genomic aberrations in pediatric B-cell acute lymphoblastic leukemia. *Front Oncol* **13**, 1217712 (2023).

17. Heyman M. A Treatment Protocol for Participants 0-45 Years With Acute Lymphoblastic Leukaemia.).

18. Moorman AV*, et al.* A novel integrated cytogenetic and genomic classification refines risk stratification in pediatric acute lymphoblastic leukemia. *Blood* **124**, 1434–1444 (2014).

19. Stanulla M, Cave H, Moorman AV. IKZF1 deletions in pediatric acute lymphoblastic leukemia: still a poor prognostic marker? *Blood* **135**, 252–260 (2020).

20. Roberts KG*, et al.* Genetic alterations activating kinase and cytokine receptor signaling in high-risk acute lymphoblastic leukemia. *Cancer Cell* **22**, 153–166 (2012).

21. Gu Z*, et al.* Genomic analyses identify recurrent MEF2D fusions in acute lymphoblastic leukaemia. *Nat Commun* **7**, 13331 (2016).

22. Inaba H, Azzato EM, Mullighan CG. Integration of Next-Generation Sequencing to Treat Acute Lymphoblastic Leukemia with Targetable Lesions: The St. Jude Children's Research Hospital Approach. *Front Pediatr* **5**, 258 (2017).

23. Gu Z*, et al.* PAX5-driven subtypes of B-progenitor acute lymphoblastic leukemia. *Nat Genet* **51**, 296–307 (2019).

24. Hirabayashi S*, et al.* ZNF384-related fusion genes define a subgroup of childhood B-cell precursor acute lymphoblastic leukemia with a characteristic immunotype. *Haematologica* **102**, 118–129 (2017).

25. Schwab C*, et al.* Integrative genomic analysis of childhood acute lymphoblastic leukaemia lacking a genetic biomarker in the UKALL2003 clinical trial. *Leukemia* **37**, 529–538 (2023).

26. Krstic A, Rezayee F, Saft L, Hammarsjo A, Svenberg P, Barbany G. Case Report: Whole genome sequencing identifies CCDC88C as a novel JAK2 fusion partner in pediatric T-cell acute lymphoblastic leukemia. *Front Pediatr* **10**, 1082986 (2022).

27. Lilljebjorn H*, et al.* Identification of ETV6-RUNX1-like and DUX4-rearranged subtypes in paediatric B-cell precursor acute lymphoblastic leukaemia. *Nat Commun* **7**, 11790 (2016).

28. Boer JM*, et al.* Favorable outcome of NUTM1-rearranged infant and pediatric B cell precursor acute lymphoblastic leukemia in a collaborative international study. *Leukemia* **35**, 2978–2982 (2021).

29. Russell LJ*, et al.* Deregulated expression of cytokine receptor gene, CRLF2, is involved in lymphoid transformation in B-cell precursor acute lymphoblastic leukemia. *Blood* **114**, 2688–2698 (2009).

30. Raffini LJ*, et al.* Panhandle and reverse-panhandle PCR enable cloning of der(11) and der(other) genomic breakpoint junctions of MLL translocations and identify complex translocation of MLL, AF-4, and CDK6. *Proc Natl Acad Sci U S A* **99**, 4568–4573 (2002).

31. Clappier E*, et al.* An intragenic ERG deletion is a marker of an oncogenic subtype of B-cell precursor acute lymphoblastic leukemia with a favorable outcome despite frequent IKZF1 deletions. *Leukemia* **28**, 70–77 (2014).

32. Khoury JD*, et al.* The 5th edition of the World Health Organization Classification of Haematolymphoid Tumours: Myeloid and Histiocytic/Dendritic Neoplasms. *Leukemia* **36**, 1703–1719 (2022).

33. Arber DA*, et al.* International Consensus Classification of Myeloid Neoplasms and Acute Leukemias: integrating morphologic, clinical, and genomic data. *Blood* **140**, 1200–1228 (2022).

34. Tesi B*, et al.* Diagnostic yield and clinical impact of germline sequencing in children with CNS and extracranial solid tumors-a nationwide, prospective Swedish study. *Lancet Reg Health Eur* **39**, 100881 (2024).

35. Ripperger T, *et al.* Childhood cancer predisposition syndromes-A concise review and recommendations by the Cancer Predisposition Working Group of the Society for Pediatric Oncology and Hematology. *Am J Med Genet A* **173**, 1017–1037 (2017).

36. Jongmans MC, *et al.* Recognition of genetic predisposition in pediatric cancer patients: An easy-to-use selection tool. *Eur J Med Genet* **59**, 116–125 (2016).

37. Goudie C, Coltin H, Witkowski L, Mourad S, Malkin D, Foulkes WD. The McGill Interactive Pediatric OncoGenetic Guidelines: An approach to identifying pediatric oncology patients most likely to benefit from a genetic evaluation. *Pediatr Blood Cancer* **64**, (2017).

38. Postema FA, *et al.* Validation of a clinical screening instrument for tumour predisposition syndromes in patients with childhood cancer (TuPS): protocol for a prospective, observational, multicentre study. *BMJ Open* **7**, e013237 (2017).

39. Zhang J, *et al.* Germline Mutations in Predisposition Genes in Pediatric Cancer. *New England Journal of Medicine* **373**, 2336–2346 (2015).

40. Byrjalsen A, *et al.* Nationwide germline whole genome sequencing of 198 consecutive pediatric cancer patients reveals a high incidence of cancer prone syndromes. *PLoS Genet* **16**, e1009231 (2020).

41. Newman S, *et al.* Genomes for Kids: The Scope of Pathogenic Mutations in Pediatric Cancer Revealed by Comprehensive DNA and RNA Sequencing. *Cancer Discov* **11**, 3008–3027 (2021).

42. Bakhuizen JJ, *et al.* Assessment of Cancer Predisposition Syndromes in a National Cohort of Children With a Neoplasm. *JAMA Netw Open* **6**, e2254157 (2023).

43. Bakhuizen JJ, *et al.* Comparison of clinical selection-based genetic testing with phenotype-agnostic extensive germline sequencing to diagnose genetic predisposition in children with cancer: a prospective diagnostic study. *Lancet Child Adolesc Health* **8**, 751–761 (2024).
