# Supplementary material for: Diagnostic yield of cancer predisposition in a nationwide prospective childhood acute leukemia cohort
Source: Nat Commun. 2026 Sep 28;17:10280. doi: 10.1038/s41467-026-78170-z (PMC13619629; doi:10.1038/s41467-026-78170-z)
Supplement: Supplementary file 2 [file 41467_2026_78170_MOESM2_ESM.pdf]

Description of Additional Supplementary Files

Supplementary Data 1. Gene list used in this study. The list was developed in 2020 and used throughout the study.

Supplementary Data 2. Overview of clinical features of the included patients (n=181) and germline findings. Each row represents a unique individual.

Supplementary Data 3. Online Mendelian Inheritance in Man (OMIM) gene panel (n=4640) and version numbers in which the gene included in the list in this study.
