# Supplementary material for: Diagnostic yield of cancer predisposition in a nationwide prospective childhood acute leukemia cohort
Source: Nat Commun. 2026 Sep 28;17:10280. doi: 10.1038/s41467-026-78170-z (PMC13619629; doi:10.1038/s41467-026-78170-z)
Supplement: Supplementary file 3 [file 41467_2026_78170_MOESM6_ESM.pdf]

nature portfolio

Corresponding author(s): Fulya Taylan
Ann Nordgren

Last updated by author(s): Jun 30, 2026

Reporting Summary

Nature Portfolio wishes to improve the reproducibility of the work that we publish. This form provides structure for consistency and transparency in reporting. For further information on Nature Portfolio policies, see our Editorial Policies and the Editorial Policy Checklist.

Statistics

For all statistical analyses, confirm that the following items are present in the figure legend, table legend, main text, or Methods section.

n/a	Confirmed	
☐	☒	The exact sample size (*n*) for each experimental group/condition, given as a discrete number and unit of measurement
☒	☐	A statement on whether measurements were taken from distinct samples or whether the same sample was measured repeatedly
☒	☐	The statistical test(s) used AND whether they are one- or two-sided<br>*Only common tests should be described solely by name; describe more complex techniques in the Methods section.*
☒	☐	A description of all covariates tested
☒	☐	A description of any assumptions or corrections, such as tests of normality and adjustment for multiple comparisons
☒	☐	A full description of the statistical parameters including central tendency (e.g. means) or other basic estimates (e.g. regression coefficient) AND variation (e.g. standard deviation) or associated estimates of uncertainty (e.g. confidence intervals)
☒	☐	For null hypothesis testing, the test statistic (e.g. *F*, *t*, *r*) with confidence intervals, effect sizes, degrees of freedom and *P* value noted<br>*Give P values as exact values whenever suitable.*
☒	☐	For Bayesian analysis, information on the choice of priors and Markov chain Monte Carlo settings
☒	☐	For hierarchical and complex designs, identification of the appropriate level for tests and full reporting of outcomes
☒	☐	Estimates of effect sizes (e.g. Cohen's *d*, Pearson's *r*), indicating how they were calculated

Our web collection on statistics for biologists contains articles on many of the points above.

Software and code

Policy information about availability of computer code

Data collection	No software was used for the collection of data
Data analysis	Germline analysis pipelines used in this study:<br>https://github.com/Clinical-Genomics/MIP<br>https://github.com/clinical-genomics-uppsala/poirot_rd_wgs<br>https://github.com/ClinicalGenomicsGBG/wgs_somatic<br>https://github.com/SMD-Bioinformatics-Lund/nextflow_wgs<br><br>Leukemia analysis pipelines used in this study:<br>https://github.com/clinical-genomics-uppsala/fluffy_hematology_wgs<br>https://github.com/Clinical-Genomics/BALSAMIC<br>https://github.com/ClinicalGenomicsGBG/wgs_somatic<br>https://github.com/SMD-Bioinformatics-Lund/nextflow_tumwgs<br><br>Data visualization and interpretation tools used in this study:<br>https://github.com/Clinical-Genomics/scout<br>https://github.com/SMD-Bioinformatics-Lund/coyote3<br>Alissa Interpret (Agilent)<br>QIAGEN Clinical Insight Interpret<br>Integrative Genomics Viewer (IGV)

Statistical analysis and figure generation were performed using GraphPad Prism version 10.5.0 or 11.0.2.

For manuscripts utilizing custom algorithms or software that are central to the research but not yet described in published literature, software must be made available to editors and reviewers. We strongly encourage code deposition in a community repository (e.g. GitHub). See the Nature Portfolio guidelines for submitting code & software for further information.

Data

Policy information about availability of data

All manuscripts must include a data availability statement. This statement should provide the following information, where applicable:

- Accession codes, unique identifiers, or web links for publicly available datasets
- A description of any restrictions on data availability
- For clinical datasets or third party data, please ensure that the statement adheres to our policy

The raw and processed genomic data generated in this study constitute sensitive personal data under the GDPR (Regulation (EU) 2016/679) and are therefore available under restricted access to safeguard participant privacy, consistent with the signed informed-consent forms and the approval of the Swedish Ethical Review Authority (no. 2021-04135). Region Uppsala is the responsible research principal and data controller.
Access is currently open for research within the approved study (no. 2021-04135), governed by a Joint Controller Agreement. Access to the data outside the approved study is under development. Future access outside the approved study will be subject to the GDPR (Regulation (EU) 2016/679), the terms of the signed informed consent, and the requirement of a study approved by the Swedish Ethical Review Authority; other legal agreements governing data access and processing may also apply. Requests regarding the status of access outside the approved study (no. 2021-04135) should be directed to Region Uppsala and the Principal Investigator (josefine.palle@akademiska.se). Response times may vary depending on the other obligations of the Principal Investigator. Source Data are provided with this paper.

Research involving human participants, their data, or biological material

Policy information about studies with human participants or human data. See also policy information about sex, gender (identity/presentation), and sexual orientation and race, ethnicity and racism.

Reporting on sex and gender

Baseline characteristics, including sex, age at diagnosis and diagnosis type, were recorded for all 181 participants (74 female, 41%; 107 male, 59%). Disaggregated by leukemia subtype: BCP-ALL, n = 138 (62 female, 76 male); AML, n = 22 (10 female, 12 male); T-ALL, n = 18 (2 female, 16 male); acute leukemias of ambiguous lineage (MPAL and AUL), n = 3 (0 female, 3 male). Sex was obtained from medical records and verified using sex-chromosome dosage from whole-genome sequencing. Gender identity was not assessed, as the cohort comprised children under 18 years of age enrolled at diagnosis. We do not report analyses stratified by sex or gender: the study evaluates germline cancer-predisposition variants, and neither prior evidence nor the biological rationale indicates that sex or gender influences the presence of these constitutional variants; the subgroup sizes would also not have supported adequately powered sex-stratified analysis. Sex-disaggregated data are provided at the individual level in the Source Data.

Reporting on race, ethnicity, or other socially relevant groupings

All disease-associated variants were evaluated without stratification by race, ethnicity, or other socially defined characteristics of study participants.

Population characteristics

The cohort comprised 181 children under 18 years of age (74 female, 107 male; median age at diagnosis 5 years, IQR 3.0–8.5) with a new (primary) diagnosis of acute leukemia, diagnosed between 17 December 2021 and 30 April 2024. The most common diagnoses were B-cell precursor acute lymphoblastic leukemia (BCP-ALL; n = 138, 76%), acute myeloid leukemia (AML; n = 22, 12%), T-cell ALL (T-ALL; n = 18, 10%), and acute leukemias of ambiguous lineage (MPAL and AUL; n = 3, 2%). Age, sex, and leukemia subtype were the principal participant-level covariates available. Cancer-predisposition prevalence was reported descriptively, including by leukemia subtype; no formal statistical comparison across subtypes was performed, owing to the small AML and T-ALL subgroup sizes. Age and sex were not incorporated as variables in the analysis. No additional covariate-relevant population characteristics were included in this analysis.

Recruitment

Children under 18 years of age with a new (primary) diagnosis of acute leukemia, diagnosed between 17 December 2021 and 30 April 2024 at one of the six pediatric oncology centers in Sweden, were eligible for inclusion; all eligible patients were approached for participation. Patients with relapsed disease were not eligible, as their initial diagnosis predated the recruitment period. Inclusion required written informed consent and an available germline DNA source. Participants received no compensation.
Because the six centers constitute all pediatric oncology centers in Sweden and enrollment was consecutive, ascertainment was population-based. To assess completeness, the cohort was benchmarked against first-diagnosis leukemia cases registered in the Swedish Childhood Cancer Registry (SCCR) over the same period. Enrollment was high at five of six centers (difference of 0–6 cases each). The largest discrepancy was at one center (Uppsala; 27 enrolled versus 44 SCCR-registered): 3 approached patients were excluded for unavailable germline whole-genome sequencing data, and the remaining shortfall (~14 cases) is attributed to difficulties in obtaining written informed consent and limited research-nurse availability during parts of the enrollment period. The unenrolled cases were concentrated in the ALL subgroup and the 0–5-year age group (24 of 32 missing cases), consistent with consent challenges and the heightened burden on families of very young children during the early diagnostic period. Reasons for declining participation were not systematically recorded, which is acknowledged as a limitation.
The principal potential bias is therefore non-random under-enrollment driven by consent and logistic factors rather than by clinical or genetic characteristics. Because these factors are not expected to correlate with germline cancer-predisposition genotype, they are unlikely to materially bias the overall prevalence estimate. The shortfall was concentrated among the

	youngest children and, correspondingly, the BCP-ALL subgroup, the subtype with the lowest observed predisposition prevalence and the largest denominator, so any residual effect would reduce the precision of subtype-specific estimates rather than systematically inflate the overall prevalence.
Ethics oversight	The study was approved by the Swedish Ethical Review Authority [Etikprövningsmyndigheten, approval no. 2021-04135 and 2019-04746].

Note that full information on the approval of the study protocol must also be provided in the manuscript.

Field-specific reporting

Please select the one below that is the best fit for your research. If you are not sure, read the appropriate sections before making your selection.

☒ Life sciences ☐ Behavioural & social sciences ☐ Ecological, evolutionary & environmental sciences

For a reference copy of the document with all sections, see nature.com/documents/nr-reporting-summary-flat.pdf

Life sciences study design

All studies must disclose on these points even when the disclosure is negative.

Sample size	No statistical method was used to predetermine sample size. The sample size was determined by the total number of eligible patients: all children under 18 years of age with a new (primary) diagnosis of acute leukemia at the six Swedish pediatric oncology centers between 17 December 2021 and 30 April 2024 who met the inclusion criteria (n = 181); patients with relapsed disease whose initial diagnosis predated the recruitment period were not eligible. Because the cohort reflects consecutive, population-based, nationwide ascertainment of newly diagnosed cases over the defined interval, it approximates the complete eligible population rather than a statistical sample, which maximizes the achievable size and minimizes selection bias and is sufficient for the descriptive aims of the study. No power calculation was performed, as the study was exploratory and descriptive without a pre-specified primary hypothesis.
Data exclusions	Patients were excluded if written informed consent was not obtained or if a suitable germline DNA source was unavailable. Eligible germline DNA sources comprised cultured skin fibroblasts (derived from skin biopsy), remission peripheral blood, or remission bone marrow. Inclusion required a new (primary) diagnosis of acute leukemia within the recruitment period; patients presenting with relapsed disease were not eligible, as their initial diagnosis predated the recruitment period of 17 December 2021 to 30 April 2024. Individuals diagnosed outside this period, or treated at centers outside Sweden, were not eligible for inclusion. No exclusion criteria were applied after data collection (no post hoc exclusions).
Replication	This was a prospective, population-based cohort study; replication was not applicable, as the study describes a single complete cohort rather than repeated experiments.
Randomization	This was an observational, population-based cohort study; randomization was not applicable, as participants were not allocated to experimental groups and no intervention was assigned.
Blinding	This was an observational, population-based cohort study of exploratory design; blinding was not applicable, as there was no group allocation or intervention to which investigators could be blinded.

Reporting for specific materials, systems and methods

We require information from authors about some types of materials, experimental systems and methods used in many studies. Here, indicate whether each material, system or method listed is relevant to your study. If you are not sure if a list item applies to your research, read the appropriate section before selecting a response.

Materials & experimental systems

n/a	Involved in the study
☒	☐ Antibodies
☒	☐ Eukaryotic cell lines
☒	☐ Palaeontology and archaeology
☒	☐ Animals and other organisms
☒	☐ Clinical data
☒	☐ Dual use research of concern
☒	☐ Plants

Methods

n/a	Involved in the study
☒	☐ ChIP-seq
☒	☐ Flow cytometry
☒	☐ MRI-based neuroimaging

Plants

Seed stocks	N/A
Novel plant genotypes	N/A
Authentication	N/A
