# Supplementary material for: Diagnostic yield of cancer predisposition in a nationwide prospective childhood acute leukemia cohort
Source: Nat Commun. 2026 Sep 28;17:10280. doi: 10.1038/s41467-026-78170-z (PMC13619629; doi:10.1038/s41467-026-78170-z)
Supplement: Supplementary file 4 [file 41467_2026_78170_MOESM7_ESM.pdf]

nature portfolio

Peer Review File

Diagnostic Yield of Cancer Predisposition in a Nationwide Prospective Childhood Acute Leukemia Cohort

Corresponding Author: Dr Fulya Taylan

This file contains all reviewer reports in order by version, followed by all author rebuttals in order by version.

Version 0:

Reviewer comments:

Reviewer #1

(Remarks to the Author)
This study by Taylan et al describes a comprehensive nation-wide Swedish study for childhood cancer predisposition in a cohort of 181 children with acute leukemia using upfront germline whole genome sequencing combined with detailed clinical phenotyping. The aim of the study appears to be to obtain information on the prevalence of these syndromes, as well as the impact of a CPS diagnosis on therapy decisions and surveillance. Overall, the study is well-designed, the representative composition of the cohort and methodology used is clear and sophisticated, and the manuscript is well written. The authors state that the data provide valuable insights but, due to the low prevalence of established germline diagnoses in their study, the impact is limited as it mainly confirms findings from previous studies (e.g. Byrjalsen et al., 2020 [74 leukemia patients, WGS-based] and Bakhuizen et al., 2024 [184 acute leukemia patients, WES-based]). The authors themselves also cite these studies in the introduction.

A major limitation of the study is that the comprehensive set-up of the study, with germline WGS for the entire cohort of 181 children with acute leukemia has been used for only a very basal analysis of coding regions in a gene panel and, consequently, has provided information for only a handful of patients. Those with a positive result were already diagnosed earlier or could have easily been preselected by clinical features (as also stated by the authors in the discussion). New or unexpected additional insights have not been made, partly perhaps because the authors have not made specific efforts to look for this to reveal additional benefits for the use of WGS for such a study.

Some observations in their study might have been worthwhile looking further into, including e.g. the case with mosaic trisomy 21. The presence of this mosaicism (already known before inclusion) was not detected in peripheral blood or bone marrow, suggesting that the mosaicism was very low in this lineage or the trisomy 21, which is very common in ALL was unrelated. Have the authors checked whether the duplicated chromosome 21 was identical in the AML, the ALL and the fibroblasts? If it was, it may suggest that the mosaicism was very low and yet resulted in a diagnosis of acute leukemia twice. It would also be intriguing to see whether the second diagnosis was somehow induced by the prior treatment, as late effects in children with increased risk are one of the reasons for CPS diagnosis in general, and therefore also important in this study.

Specific comments:
1. The abstract does not formulate a clear aim of the study, and also in the rest of the study this is basically lacking. If the aim is to identify the prevalence of CPS in pediatric acute leukemia the power appears insufficient. If the aim is to establish whether (trio) WGS is a recommendable strategy for all patients the answer is probably no.
2. In the abstract, the authors make a statement of the prevalence of AML, T-ALL and BCP-ALL relative to each other. These percentages are likely very inaccurate as numbers are simply too low, and we would recommend not to use them. In fact, the authors indicate this themselves as well in the results.
3. The authors' conclusion and recommendation do not fully follow logically from the results presented and are not entirely clear. The manuscript states that "their findings reveal underrecognized, clinically actionable germline predisposition and support integrated workflows combining phenotype-guided selection with routine germline WGS at diagnosis." However, in this study a WGS-based 189-gene panel was offered to all children with acute leukemia, without comparison to outcomes based on phenotype-guided selection. As we understand the discussion, the authors do not necessarily recommend continuing this approach as standard practice, unless sufficient resources are available - and then primarily for research purposes rather than for routine diagnostics. If this interpretation is correct, this should be stated more explicitly in the

abstract. In addition, it is unclear what the authors mean by “routine germline WGS at diagnosis” Does this refer to a broad gene panel, or to open, genome-wide analysis?
4. Line 449: What were the predefined somatic genetic criteria? Please specify these, as we cannot find a description of these criteria in the article in which ChiCap was previously described (reference 23).
5. The authors state: “Based on these findings, we recommend gene-panel testing for all children with somatic genetic findings suggestive of a predisposition.” We have two questions regarding this recommendation: 1) Could the authors specify what they mean by somatic genetic findings suggestive of a predisposition? (See also point 4) 2) Could the authors clarify what they mean by gene-panel testing? Are they referring to a broad gene panel, such as the one used in this study, or a leukemia-specific gene panel?
6. What was the reason to include patients with confirmed diagnosis of Down syndrome? Have these patients undergone the same genetic analysis as the other samples, and what was the reason to do this?
7. Although more and more pediatric oncology centers are moving from WES to WGS, WGS is certainly not yet available in all centers. Could the authors specify what the added value of WGS was compared with WES in this study? Were there variants detected by WGS that would not have been captured by WES? To what extent were intronic variants assessed, and how were these variants analyzed?
8. In the methods, a clear description of the trio analysis is lacking. Is there a restricted focus on de novo variants? Which methods are used to identify de novo variants? Considering the long list of OMIM genes included in the analysis of these selected cases, there must be a long list of pathogenic or likely pathogenic variants as well. How was priorization performed here? And why were variants not reported in the list?
9. In the text (both abstract and Results), the authors are not very clear about the de novo nature of the DNMT3A variant. For example, in the Results (line 179) they state “a DNMT3A variant was identified by trio”. Apart from the fact that it is grammatically incomplete, it also does not make clear that the variant was de novo and why this variant was considered relevant.
10. The Methods section describes the standard-of-care somatic diagnostic tests, but not germline testing. Was there no standard approach for germline diagnostics. Please clarify.
11. The authors compare their cohort with data from the Swedish Childhood Cancer Registry and (rightly) conclude that the representativeness of their cohort is good. However, it would also be informative to provide absolute numbers. How many of the 32 patients who were not enrolled in the study (based on SCCR data) were approached for participation? Is it known what the reasons were for declining study participation?
12. In the Discussion, the authors state that the prevalence of ChiCap syndromes (3.3%) in their cohort is lower than that reported in previous studies, such as Byrjalsen et al. and Bakhuizen et al. Which definition of a ChiCap syndrome did the authors use for this comparison?
For their own cohort, only pediatric-onset leukemia predisposition syndromes are used fort his comparison. In contrast, Byrjalsen et al. distinguish between pediatric-onset and adult-onset cancer predisposition syndromes (CPSs), while Bakhuizen et al. classify CPSs as causative, possibly causative, or unlikely causative. Were these differences in classification considered when comparing prevalence across studies?
The authors suggest that the observed differences may be due to stringent variant reporting criteria and the inclusion of only newly diagnosed cases. However, both the Danish cohort (Byrjalsen et al.) and the Dutch cohort (Bakhuizen et al.) also included only newly diagnosed cases, and we question whether their variant reporting criteria were substantially less stringent.
13. In the Discussion, the authors state that “a phenotype-first approach based on ChiCaP criteria combined with reflex testing based on somatic findings would have identified all patients with pathogenic variants in leukemia predisposition genes.” What did standard germline genetic diagnostics in children with leukemia look like prior to this study? Was this phenotype-driven? And was such testing no longer performed once patients were enrolled in this study? For example, was targeted TP53 germline testing no longer carried out in a patient with low-hypodiploid ALL?

Minor comments:
• At what point were patients approached for participation in this study (i.e. the time interval between leukemia diagnosis and informed consent)?
• The authors mention as a limitation of their study the “failure to detect mosaic variants present at levels below 20%” (lines 409–410). However, we do not find any description in the Methods section of a variant allele frequency (VAF)-based filter. Were variants with a VAF <20% directly filtered out? If so, please specify this explicitly in the Methods section.
• Line 417: What do the authors mean by “patients meeting high-risk clinical criteria”? Please specify which criteria are being referred to.

Reviewer #2

(Remarks to the Author)
I co-reviewed this manuscript with one of the reviewers who provided the listed reports. This is part of the Nature Communications initiative to facilitate training in peer review and to provide appropriate recognition for Early Career Researchers who co-review manuscripts.

Reviewer #3

(Remarks to the Author)
This manuscript describes the clinical yield of universal predisposition screening on childhood acute leukemia patients. Given the generally accepted rate of ~5-15% predisposing-allele associated cancers, it would be helpful to know if applying universal sequencing would be clinically appropriate in a “real world” unselected population. The study found a low prevalence of predisposing alleles (~5%); however, most (6 of 9) would be missed without this screening. Most of the

mutation positive (for known leukemia genes) cases had met clinical criteria based on ChiCaP criteria, but the three with sold tumor mutations did not. The authors argue against universal screening for mutations but referral to screening if ChiCaP criteria eg., family history and/or other defined abnormalities) are met.

Major:
1. Lines 319-325; 334-335; 359-361: Given the relatively low prevalence of pathogenic variants identified using the ChiCaP framework, please discuss whether incorporating additional cancer predisposition gene lists or datasets (beyond the 189 ChiCaP genes) could complement the current analysis. In particular, comment on which alternative or expanded gene sets might be appropriate and how their inclusion could potentially influence variant yield and interpretation. The investigators settled on the pre-designed 189 gene set but a post-hoc analysis could be fruitful. (and the data is available)

Minor:
2. Lines 160-161: The manuscript states that 176 samples were derived from cultured skin fibroblasts and five samples from remission blood or bone marrow. Please clarify whether any patients identified as having ChiCaP syndromes were among these five cases. If so, discuss whether the use of remission blood or bone marrow as a germline DNA source could introduce bias and why or why not.

3. Table 1:
a. The use of “N” for the characteristic “ChiCaP criteria available” (rows 10–16, including the header) is potentially misleading, as it may be misinterpreted at first glance to indicate that the ChiCaP criteria available for 181 patients. Please revise the labeling/table to improve clarity.
b. Add a citation for the ChiCaP criteria.
c. As family history and detailed ChiCaP criteria are described later in the Results section and in the Supplementary Materials, please expand the table legend to briefly define how family history was assessed and include explicit references to the relevant supplementary tables or sections where the full criteria (including family history and additional features) are described.

4. Lines 166-167: “The manuscript states that “trio WGS—sequencing of the proband and both unaffected parents—was performed in 11 cases.” Please clarify whether all parents in these 11 trios were indeed free of any cancer diagnosis. Additionally, clarify whether the term “proband” refers exclusively to the affected child or could also include a parent with a cancer diagnosis.

5. Lines 237-242; 347-349: Please indicate whether there are previous publications reporting associations between the mutated genes identified in this study and specific leukemia subtypes. If exists, expand the discussion to place the current findings in the context of prior reports.

6. Lines 263-265: As the ChiCaP criteria constitute a central methodological component of this study, the description of the use of “the structured ChiCaP criteria form described in detail by Tesi et al. (2024)” should be introduced earlier in the results section, rather than appearing only at the last part of the results.

7. The 181 patients were lower than expected for the ~28+ months of recruitment – what reason were the 32 patients missed? (ie., did they get treatment outside of Sweden, or refused consent, or other reasons?). There is some confusion about diagnosis date and referral to the laboratory dates – is this the reason? (paragraph after line 558)

8. Unclear why the DMT3A mutation was not found in the individual case, but then found in the trio analysis? Actually this reviewer just figured out that DNMT3A was not part of the 189 gene panel. Which brings up another question - why not evaluate all patients with the OMIM list (without needing to do the trio).

Version 1:

Reviewer comments:

Reviewer #1

(Remarks to the Author)
We thank the authors for extensively addressing our comments. We believe that the revisions made have substantially improved the readability of the manuscript. The aim of the study is clearer and conclusions are better in line with the results. The validity of this three-pronged diagnostic strategy in clinical practice is still a matter of debate. In the Swedish context, where WGS is already implemented as standard clinical practice, this strategy makes perfect sense, but for many other locations it will not be cost-effective.

We have a few additional points that the authors may take into consideration:
- The authors have explained, in response to our questions, that all (likely) pathogenic variants that have now been detected with WGS would, in theory, also have been detectable with WES. However, this information is still missing in the manuscript. In our view, it would be useful to briefly mention this in the discussion. In addition, the methods section still lacks further explanation of how intronic variants were assessed. For example, was RNA-seq data used for intronic variants with suspected splice predictions? Precisely because this study uses WGS, this is relevant and useful information.

- Regarding our question related to the identity of the duplicated chr 21 in the different malignancies in the patient suspected of mosaic trisomy 21. We believe, however, that the statement: “establishing a shared constitutional origin” (page 15) is too strong because the chance that this has occurred coincidently cannot be ignored. Instead, “..is in line with..” may be a better formulation.
- On page 13 they refer to “…a TP53-related cancer predisposition syndrome…”. Although this wording may follow the rend to use gene-based references to syndromes, the authors may wish to consider whether Li–Fraumeni syndrome should be referred to explicitly by name in this section. Given its prominence as one of the best-recognised hereditary cancer predisposition syndromes, this would help to make the clinical context clearer for the reader.

Reviewer #2

(Remarks to the Author)
I co-reviewed this manuscript with one of the reviewers who provided the listed reports. This is part of the Nature Communications initiative to facilitate training in peer review and to provide appropriate recognition for Early Career Researchers who co-review manuscripts.

Reviewer #3

(Remarks to the Author)
The authors thoughtfully considered the comments and revised the manuscript with these in mind. This did not lead to any changes in the approach or the data presented, but will aid the reader in gaining an accurate assessment of the study purpose and yield. As the studies purpose did not include "discovery" of potential new relevant variants outside of the gene panel selected, such discovery potential is unrealized; but certainly falls out of the intended scope.

Open Access This Peer Review File is licensed under a Creative Commons Attribution 4.0 International License, which permits use, sharing, adaptation, distribution and reproduction in any medium or format, as long as you give appropriate credit to the original author(s) and the source, provide a link to the Creative Commons license, and indicate if changes were made.
In cases where reviewers are anonymous, credit should be given to 'Anonymous Referee' and the source.
The images or other third party material in this Peer Review File are included in the article’s Creative Commons license, unless indicated otherwise in a credit line to the material. If material is not included in the article’s Creative Commons license and your intended use is not permitted by statutory regulation or exceeds the permitted use, you will need to obtain permission directly from the copyright holder.
To view a copy of this license, visit https://creativecommons.org/licenses/by/4.0/

NCOMMS-25-103638 Diagnostic Yield of Cancer Predisposition in Childhood Acute Leukemia: A Nationwide Prospective Swedish Study

REVIEWER COMMENTS & RESPONSES TO THE COMMENTS

We are deeply grateful to the three reviewers for their careful reading of our manuscript and for the detailed, insightful comments that have significantly strengthened this work. We thank them for the time and expertise they have devoted to evaluating our study. In the following, we respond to each comment in turn; we hope the reviewers will forgive the length of some of our replies, which reflects our wish to address every point thoroughly.

Reviewer #1 (Remarks to the Author):

This study by Taylan et al describes a comprehensive nation-wide Swedish study for childhood cancer predisposition in a cohort of 181 children with acute leukemia using upfront germline whole genome sequencing combined with detailed clinical phenotyping. The aim of the study appears to be to obtain information on the prevalence of these syndromes, as well as the impact of a CPS diagnosis on therapy decisions and surveillance. Overall, the study is well-designed, the representative composition of the cohort and methodology used is clear and sophisticated, and the manuscript is well written. The authors state that the data provide valuable insights but, due to the low prevalence of established germline diagnoses in their study, the impact is limited as it mainly confirms findings from previous studies (e.g. Byrjalsen et al., 2020 [74 leukemia patients, WGS-based] and Bakhuizen et al., 2024 [184 acute leukemia patients, WES-based]). The authors themselves also cite these studies in the introduction.

A major limitation of the study is that the comprehensive set-up of the study, with germline WGS for the entire cohort of 181 children with acute leukemia has been used for only a very basal analysis of coding regions in a gene panel and, consequently, has provided information for only a handful of patients. Those with a positive result were already diagnosed earlier or could have easily been preselected by clinical features (as also stated by the authors in the discussion). New or unexpected additional insights have not been made, partly perhaps because the authors have not made specific efforts to look for this to reveal additional benefits for the use of WGS for such a study.

Response: We sincerely thank the reviewer for their thoughtful evaluation and for recognizing the strengths of our study design, methodology, and manuscript. We are grateful for this constructive feedback and welcome the opportunity to clarify the distinctive contributions of our work.

While we acknowledge that our findings align with those of previous studies, including Byrjalsen et al., 2020[1] and Bakhuizen et al., 2024[2], our three-pronged diagnostic strategy (systematic phenotyping, germline WGS-based *in silico* 189-gene panel encompassing clinically actionable ChiCaP genes combined with tumor sequencing of positive cases) has strengths that, we believe, make our results extend beyond confirmation of prior observations. The Bakhuizen et al., 2024 study, which has a similar design, used data from both whole exome and genome sequencing, and a smaller set of genes were analyzed (either 143- or 49-gene panels depending on obtained consent) combined with tumor WES, WGS or RNA-seq data if available. The Danish study (Byrjalsen et al., 2020), which also is germline WGS-based, did not include sequencing analysis of paired leukemia samples. None of the studies included data on the observed clinical implications of their findings for treatment and surveillance of included patients.

We recognize that the prevalence of established leukemia associated predisposition genes is relatively low in our cohort. However, we believe that this finding is informative, especially since the WGS-based analysis was comprehensive. In fact, we took advantage of the WGS data to interrogate different variant types within the *in-silico* gene panel used, including SNVs, indels, CNVs and structural variants.

We could have analyzed the entire genome. However, the intention of this study was to assess diagnostic yield and clinical utility of our three-pronged diagnostic strategy to be used in a clinical setting, where analyses are restricted to a set of well-established ChiCaP genes in all children and trio-WGS in cases with high clinical suspicion of ChiCaP. Novel gene discovery was not the intention of this study. However, we show how trio WGS identified a *de novo DNMT3A* variant in a patient with T-ALL and syndromic features, supporting our diagnostic strategy.

We have now revised the abstract and the discussion to clarify this and we also added a new figure (Figure 1) which visualizes our three-pronged diagnostic strategy.

Some observations in their study might have been worthwhile looking further into, including e.g. the case with mosaic trisomy 21. The presence of this mosaicism (already known before inclusion) was not detected in peripheral blood or bone marrow, suggesting that the mosaicism was very low in this lineage or the trisomy 21, which is very common in ALL was unrelated. Have the authors checked whether the duplicated chromosome 21 was identical in the AML, the ALL and the fibroblasts? If it was, it may suggest that the mosaicism was very low and yet resulted in a diagnosis of acute leukemia twice. It would also be intriguing to see whether the second diagnosis was somehow induced by the prior

treatment, as late effects in children with increased risk are one of the reasons for CPS diagnosis in general, and therefore also important in this study.

Response: We thank the reviewer for this insightful suggestion. We identified informative SNPs across the AML (WGS), ALL (WGS), and skin fibroblasts (SNP array). Variant allele frequencies from WGS and signal intensities from the SNP array using 24 informative SNPs confirm that the same chromosome 21 was duplicated in all three sample types. In the skin fibroblasts, the mosaic trisomy 21 level is approximately 90%. In contrast, the trisomy 21 signal in AML and ALL is considerably lower and in line with the blast counts of 20% in AML and 51% in ALL. It is non-detectable in remission bone marrow using WGS, indicating that mosaicism for trisomy 21 in the hematopoietic compartment is below 20%.

The BCP-ALL was diagnosed four years after the initial AML. Although a therapy-related origin is an important consideration given this interval, several lines of evidence favor a second primary leukemia arising in the context of constitutional predisposition. The ALL-sample harbored deletions of both *BTG1* and *IKZF1*. The *BTG1* deletions occur at a frequency of 25–29% in Down syndrome-associated ALL (DS-ALL) compared to ~9% in non-DS BCP-ALL[3, 4, 5], and *IKZF1* deletions at ~35%[6]. Therapy-related ALL is a recognized entity distinct from *de novo* ALL, characteristically enriched for *KMT2A* rearrangements, particularly following exposure to topoisomerase II inhibitors, and chromosomes 5 and 7 abnormalities[7, 8]. The genetic profile of the ALL in our patient - harboring *IKZF1* and *BTG1* deletions without *KMT2A* rearrangement - is inconsistent with a therapy-related origin and instead consistent with DS-ALL biology, supporting its origin as a predisposition-driven second primary leukemia from a rare hematopoietic progenitor carrying the mosaic trisomy 21. Together with the confirmation that the same parental chromosome 21 was duplicated across the AML, ALL, and skin fibroblasts, these findings further support a constitutional predisposition-driven origin.

We have clarified this on the manuscript in the results section under the title of “Somatic Findings in Cases with Positive ChiCaP” (lines 239-250, page 13), discussed the risk of missing mosaicism with our approach in the discussion (lines 429-434, page 21) and we also added our analysis in the methods (lines 658-668, pages 30-31).

Specific comments:

1. The abstract does not formulate a clear aim of the study, and also in the rest of the study this is basically lacking. If the aim is to identify the prevalence of CPS in pediatric acute leukemia the power appears insufficient. If the aim is to establish whether (trio) WGS is a recommendable strategy for all patients, the answer is probably no.

Response: We thank the reviewer for this important observation. We agree that the aim of our study was not sufficiently clear in the original manuscript and have now revised both the abstract and the introduction (lines 143-148, page 9) accordingly.

Our aim was to evaluate the diagnostic yield and clinical utility of a three-pronged diagnostic strategy uniformly applied across centers, performed at diagnosis an unselected, population-based cohort of all children diagnosed with acute leukemia in Sweden. The strategy included systematic phenotyping, germline WGS-based *in silico* 189-gene panel combined with tumor sequencing of positive cases.

2. In the abstract, the authors make a statement of the prevalence of AML, T-ALL and BCP-ALL relative to each other. These percentages are likely very inaccurate as numbers are simply too low, and we would recommend not to use them. In fact, the authors indicate this themselves as well in the results.

Response: We thank the reviewer for this insightful comment and fully agree that the small sample sizes preclude meaningful comparison of prevalence between subtypes. We have removed the comparative statement from the abstract.

3. The authors' conclusion and recommendation do not fully follow logically from the results presented and are not entirely clear. The manuscript states that "these findings reveal underrecognized, clinically actionable germline predisposition and support integrated workflows combining phenotype-guided selection with routine germline WGS at diagnosis." However, in this study a WGS-based 189-gene panel was offered to all children with acute leukemia, without comparison to outcomes based on phenotype-guided selection. As we understand the discussion, the authors do not necessarily recommend continuing this approach as standard practice, unless sufficient resources are available - and then primarily for research purposes rather than for routine diagnostics. If this interpretation is correct, this should be stated more explicitly in the abstract. In addition, it is unclear what the authors mean by "routine germline WGS at diagnosis" Does this refer to a broad gene panel, or to open, genome-wide analysis?

Response: We thank the reviewers for this important observation. We fully agree that our original conclusions and recommendations were insufficiently clear, and we have carefully revised them throughout the manuscript.

We acknowledge that we have not designed our study to perform parallel investigations such as: A) a phenotype-guided selection approach, B) a genotype-first approach, C) a combined genotype-phenotype approach. We performed only option C combined with tumor sequencing of positive cases and evaluated retrospectively whether option A would

have identified all positive cases. We have clarified our three-pronged approach in the abstract and in the introduction. We also revised our conclusions in the abstract and in the discussion.

In well-resourced settings such as Sweden, where all children with acute leukemia already undergo tumor-normal WGS analysis at diagnosis as part of standard clinical practice, we believe continuing this three-pronged approach to detect ChiCaP is beneficial. In settings with limited resources, a phenotype-guided selection strategy appears to be a reasonable alternative. However, some cases might escape detection, such as patients with i) *de novo* variants, ii) variants in low-penetrant genes, iii) no phenotypic features, and iv) no material for somatic analyses. We emphasize that the choice of testing strategy should reflect local policies and resources, including the capacity for pre-test genetic counselling, as well as the decision of whether to restrict analysis to genes directly relevant to the child's cancer or to extend it to a broader set encompassing adult-onset disease. We also now emphasize that further research is needed to evaluate whether broader genomic approaches would identify additional clinically relevant findings. Finally, regarding the reviewers' question about "routine germline WGS at diagnosis", we apologize for this local expression, and we have removed it from the abstract. To clarify, “routine germline WGS at diagnosis" in Sweden means analysis of a whole-genome sequencing based predefined gene panel, not an open genome-wide analysis.

4. *Line 449: What were the predefined somatic genetic criteria? Please specify these, as we cannot find a description of these criteria in the article in which ChiCaP was previously described (reference 23).*

Response: We thank the reviewer for this important observation. We agree that the original phrasing was insufficiently specific. We have now revised the sentence to explicitly list all somatic genetic criteria that triggered completion of the supplementary ChiCaP criteria form. The revised text (lines 503-508, page 24, lines 636-637 page 29) now reads:

"A supplementary ChiCaP criteria form was completed exclusively for children with leukemia who exhibited an unusually severe toxicity reaction or were diagnosed with one of the following conditions: AML or MDS with karyotype monosomy 7/7q−, BCP-ALL with low hypodiploidy (31–39 chromosomes), or BCP-ALL with iAMP21.”

“A somatic finding with VAF above 40% in genes associated with leukemia predisposition also triggered a reflex testing in the clinical setting.”

5. The authors state: “Based on these findings, we recommend gene-panel testing for all children with somatic genetic findings suggestive of a predisposition.” We have two

questions regarding this recommendation: 1) Could the authors specify what they mean by somatic genetic findings suggestive of a predisposition? (See also point 4) 2) Could the authors clarify what they mean by gene-panel testing? Are they referring to a broad gene panel, such as the one used in this study, or a leukemia-specific gene panel?

Response: We thank the reviewer for this important clarification request.

1. In the supplementary ChiCaP criteria, the somatic genetic findings considered suggestive of a predisposition are: AML or MDS with monosomy 7/7q−; BCP-ALL with low hypodiploidy (31–39 chromosomes); and BCP-ALL with iAMP21. Beyond these formal criteria, we also regarded a somatic variant with VAF >40% in a gene associated with leukemia predisposition as suggestive of a predisposition, and this is reflected in our current recommendation. This has been added to the methods section where we describe leukemia analysis (lines 636–637 and 655–657, pages 29-30).
2. We think a leukemia predisposition specific gene panel is enough to identify germline leukemia predisposition when somatic genetic findings are suggestive of a predisposition. We have clarified this in the manuscript, and we believe each center should decide what is most suitable for them. To streamline the diagnostic workflow, we chose one broader gene panel, the ChiCaP 189-gene panel, that covers both leukemia and solid tumors.

6. What was the reason to include patients with confirmed diagnosis of Down syndrome? Have these patients undergone the same genetic analysis as the other samples, and what was the reason to do this?

Response: We thank the reviewer for this important question. Our study was designed as a prospective, population-based investigation, and we therefore included all children diagnosed with cancer, irrespective of any pre-existing diagnosis of a genetic predisposition syndrome, including Down syndrome.

There were two key reasons for this approach. First, patients with a known predisposition syndrome may carry additional, previously unrecognized germline variants that contribute to their cancer risk. Although dual diagnoses are uncommon, excluding these patients a priori would risk missing clinically relevant findings.

Second, from a practical standpoint, the Genomic Medicine Sweden (GMS) infrastructure offers tumor–normal genome sequencing combined with careful phenotyping to all children with cancer, regardless of previously known genetic conditions. Because the somatic analysis is performed as a paired tumor–normal approach, germline data is already generated as part of the standard diagnostic workflow in all children.

We added a sentence about this in the methods section under “Study Design, Patients, and Workflow” (lines 483-488, page 23).

7. Although more and more pediatric oncology centers are moving from WES to WGS, WGS is certainly not yet available in all centers. Could the authors specify what the added value of WGS was compared with WES in this study? Were there variants detected by WGS that would not have been captured by WES? To what extent were intronic variants assessed, and how were these variants analyzed?

Response: We thank the reviewer for this important question.

In this particular study, the pathogenic and likely pathogenic variants identified were SNVs and indels located within exons. Therefore, in this specific case, the variants detected by WGS would have been captured by WES or targeted gene panels.

In general, short-read WGS has better possibilities to detect SNVs, indels, CNVs, SVs, MEIs and STR expansions. In our study design, WGS is used for both germline and paired tumor-normal analysis. In this clinical study, we looked for SNVs, indels, CNVs and SVs located in the exon or exon-intron boundaries.

8. In the methods, a clear description of the trio analysis is lacking. Is there a restricted focus on de novo variants? Which methods are used to identify de novo variants? Considering the long list of OMIM genes included in the analysis of these selected cases, there must be a long list of pathogenic or likely pathogenic variants as well. How was priorization performed here? And why were variants not reported in the list?

Response: We thank the reviewer for this important comment. We have added a detailed description of the trio WGS analysis.

The trio WGS analysis was not restricted to *de novo* variants. Variants were evaluated under multiple inheritance models, including *de novo*, autosomal recessive (homozygous and compound heterozygous), and X-linked inheritance. For compound heterozygous candidates, two variants *in trans* in the same gene were required for the gene to be considered disease-causing.

Trio-WGS data was analyzed with Mutation Identification Pipeline (MIP; https://github.com/Clinical-Genomics/MIP)[9] which includes calling of SNVs, INDELs, structural variants, short tandem repeats, mobile element insertions and SMN1 copy number. Specifically, SNVs and INDELs were called with DeepVariant. Annotation of inheritance patterns was done using “genmod models” (https://github.com/Clinical-Genomics/genmod), part of the Mutation Identification Pipeline, MIP.

Called variants were annotated and prioritized using “genmod”, an in-house pathogenicity scoring system integrated in MIP. This tool assigns a rank score to each variant based on multiple parameters, including allele frequency in the general population, predicted protein impact, gene intolerance scores, inheritance model compatibility, variant consequence, evolutionary conservation, variant call quality, predicted deleteriousness, clinical significance (e.g., ClinVar), and splicing predictions. Variants classified as Likely Pathogenic or Pathogenic in ClinVar with a gold star review status were always retained regardless of frequency or consequence. Only sequence variants with an allele frequency below 1% in the general population affecting exons or exon-intron boundaries were considered. The genes included in the analysis comprised all OMIM Morbid genes known at the time of analysis (n=4640), provided in supplementary table 4. Despite the large number of genes analyzed, the stringent filtering criteria and prioritization system ensure that only a small number of clinically relevant variants emerge per case.

Carriership for autosomal recessive diseases is not reported. Only variants classified as class 4 (Likely Pathogenic) or class 5 (Pathogenic), along with selected variants of uncertain significance (VUS) in strong candidate genes matching the patient phenotype, are reported. All clinically relevant findings - the diagnostic variants - are reported in the main results of the manuscript.

We added a new section where we describe “trio genome sequencing” under the methods section (lines 577-602, pages 27-28).

9. In the text (both abstract and Results), the authors are not very clear about the de novo nature of the DNMT3A variant. For example, in the Results (line 179) they state “a DNMT3A variant was identified by trio”. Apart from the fact that it is grammatically incomplete, it also does not make clear that the variant was de novo and why this variant was considered relevant.

Response: We thank the reviewer for this observation. We apologize for the unclear and grammatically incomplete sentence. We have revised both the abstract and the results section to explicitly state that the *DNMT3A* variant (NM_022552.5: c.2635A>G, p.(Asn879Asp)) was identified as a *de novo* event through trio-WGS and classified as likely pathogenic based on ACMG criteria. The revised text also clarifies that this case is reported separately from the ChiCaP panel findings.

We revised the abstract and the results section (lines 188-191, page 11).

10. The Methods section describes the standard-of-care somatic diagnostic tests, but not germline testing. Was there no standard approach for germline diagnostics? Please clarify.

Response: We thank the reviewer for this important observation.

No systematic germline testing was conducted within the clinical practice prior to this study and targeted germline testing has relied on the initiative of the clinicians or diagnostic laboratory to recommend follow-up analyses due to phenotype or somatic findings triggering genetic testing. Although we lack data on the extent of germline testing performed in Sweden in children with leukemia before the study, it is reasonable to expect that low hypodiploidy in BCP-ALL would trigger germline testing for *TP53*. Likewise, it is reasonable to expect that a finding of *CEBPA* mutation in AML with variant allele frequency (≥40%) or in a biallelic pattern would have triggered confirmation/exclusion of germline origin in a suitable germline sample.

We have added a clarification in the methods section (lines 636-638, page 29 and 655-657, page 30).

11. The authors compare their cohort with data from the Swedish Childhood Cancer Registry and (rightly) conclude that the representativeness of their cohort is good. However, it would also be informative to provide absolute numbers. How many of the 32 patients who were not enrolled in the study (based on SCCR data) were approached for participation? Is it known what the reasons were for declining study participation?

Response: We thank the reviewer for this important comment regarding the representativeness of our cohort. We updated the SCCR data. We have now included absolute numbers comparing enrollment across centers, leukemia subtypes, and age groups. See the tables below.

Table 1: Based on participating center

**Center**	**ChiCaP study**	**SCCR data**	**Difference**
**Gothenburg**	62	60	-2
**Lund**	30	36	6
**Uppsala**	27	44	17
**Stockholm**	27	32	5
**Linköping**	18	18	0
**Umeå**	17	23	6
**Total**	181	213	**32**

Table 2: Based on leukemia subtype

Leukemia type	ChiCaP study	SCCR data	Difference
**ALL (B + T)**	156	182	26
**AML**	22	26	4
**Other**	3	2	-1
**Unspecified**	0	3	3
**Total**	181	213	**32**

Table 3: Based on age group

Age	ChiCaP study	SBCR data	Difference
**0-5 years**	103	127	24
**6-10 years**	44	46	2
**11-15 years**	24	31	7
**16-18 years**	10	9	-1
**Total**	181	213	**32**

All eligible patients were approached for participation. The largest discrepancy by center was observed in Uppsala, where 27 patients were enrolled compared with 44 first leukemia diagnoses registered in the Swedish Childhood Cancer Registry (SCCR) during the same period. To understand this gap, we contacted the Uppsala pediatric oncology team. Initially, an additional 3 patients had been recruited but were excluded from the study due to unavailable germline WGS data. The remaining discrepancy of approximately 14 cases is primarily attributed to challenges in obtaining written informed consent and limited research nurse availability during parts of the enrollment period in Uppsala.

For the other five centers, the differences between the study cohort and the SCCR ranged from 0 to 6 cases, indicating high enrollment rates.

Regarding leukemia subtypes, the ALL group shows a difference of 26 patients between the SCCR and GMS ChiCaP. The largest difference by age group was observed in the 0–5 years category (24 of 32 missing patients). This likely reflects, at least in part, the consent challenges described above, and that parents of very young children may be particularly overwhelmed during the early diagnostic period.

We acknowledge that we are unable to provide a detailed breakdown of specific reasons for declining study participation, as declination reasons were not systematically recorded during the consent process. This is a limitation that we have now noted in the discussion of the revised manuscript (lines 449-450, page 22).

We added the following sentence to the Methods section under the title of "Study Design, Patients, and Workflow" between lines 486-488 (page 23): "Not all eligible patients could be enrolled, primarily due to logistic challenges in obtaining written informed consent."

12. In the Discussion, the authors state that the prevalence of ChiCaP syndromes (3.3%) in their cohort is lower than that reported in previous studies, such as Byrjalsen et al. and Bakhuizen et al. Which definition of a ChiCaP syndrome did the authors use for this comparison?

For their own cohort, only pediatric-onset leukemia predisposition syndromes are used for this comparison. In contrast, Byrjalsen et al. distinguish between pediatric-onset and adult-onset cancer predisposition syndromes (CPSs), while Bakhuizen et al. classify CPSs as causative, possibly causative, or unlikely causative. Were these differences in classification considered when comparing prevalence across studies? The authors suggest that the observed differences may be due to stringent variant reporting criteria and the inclusion of only newly diagnosed cases. However, both the Danish cohort (Byrjalsen et al.) and the Dutch cohort (Bakhuizen et al.) also included only newly diagnosed cases, and we question whether their variant reporting criteria were substantially less stringent.

Response: We thank the reviewer for this thoughtful and important comment. We fully agree that differences in CPS classification across studies must be carefully considered when comparing prevalence estimates. We have revised our Discussion to address these differences. We agree with the reviewer that it is possible to compare our data with the Danish cohort (Byrjalsen et al.)[1] and the Dutch cohort (Bakhuizen et al.)[2] given that they also included newly diagnosed cases and that their variant reporting makes it possible to compare the results when the same definition of ChiCaP is used, i.e. P/LP variants in genes with an established association with childhood leukemia and consistent with known inheritance pattern.

To systematically address the reviewer's concern, we have constructed a detailed cross-study comparison table (below) that traces how CPS prevalence changes depending on which definition is applied, specifically for leukemia patients across the five relevant studies including Byrjalsen et al. and Bakhuizen et al. (now included as supplementary table 3)

	Zhang 2015[10] (excl. Down syndrome)	Byrjalsen 2020[1] (incl. Down syndrome)	Newman 2021[11] (excl. Down syndrome)	Bakhuizen 2023[12] (incl. Down syndrome)	Bakhuizen 2024[2] (Supplemental table 8 in the study)	Bakhuizen 2024[2] (Table 2 in the study) Newly sequenced cohort	This study (incl. Down syndrome)
Total number of children with leukemia in the study (n)	588	74	126	216	280	184	181
P/LP variants in all CPS genes (child+adult, genes for all cancer types) regardless of inheritance pattern	26 (4.4%)	11 (14.9%)	16 (12.7%)	12 (5.5%)	20 (7.1%)	7 (3.8%)	9 (5.0%)
P/LP variants in leukemia-associated CPS genes in line with inheritance pattern	16 (2.7%)	6 (8.1%)	9 (7.1%)	11(5%)	NA	1 (0.5%)	6 (3.3%)
P/LP variants in leukemia-associated CPS genes in line with inheritance pattern (excl. Down syndrome)	16 (2.7%)	3 (4%)	4 (3.2%)	2 (0.9%)	NA	1 (0.5%)	3 (1.7%)

When the comparison is restricted to P/LP variants in genes with an established association with childhood leukemia and matching inheritance pattern - the definition used for ChiCaP syndromes in our study -, the prevalence across studies narrows to 0.5-8.1%, and drops further to 0.5-4% when Down syndrome is excluded, with our 1.7% falling within both of these ranges.

First, the CPS prevalence figures (i.e. P/LP variants in all CPS genes (child+adult, genes for all cancer types) regardless of inheritance pattern) vary substantially across studies (3.8–14.9%), but much of this variation is driven by variants in adult-onset CPS genes and genes without an established causal link to leukemia (e.g., *CDC73, SDHC , CHEK2, MUTYH, APC, BARD1, RET, VHL, CDH1, SDHA, RTEL*) as well as how heterozygous carriership of autosomal recessive genes are reported. These variants are included in the broader CPS counts of Byrjalsen et al., Zhang et al., and Newman et al., but do not represent childhood leukemia predisposition syndromes as defined in our study. Bakhuizen et al. explicitly classified such findings as "unlikely causative" or "possibly causative".

Second, when the comparison is restricted to P/LP variants in genes/conditions with an established association with leukemia and following the expected inheritance pattern, the prevalence narrows considerably across studies: 7.1% (Newman), 8.1% (Byrjalsen, driven by Down syndrome cases), 3.3% (our study, also driven by Down syndrome cases), and 2.7% (Zhang et al).

Third, when Down syndrome is excluded, the leukemia-associated CPS prevalence in all studied ranges between 0.5% and 4%.

Fourth, regarding the reviewer's specific questions about variant reporting stringency and patient enrolment: we acknowledge that both the Danish (Byrjalsen et al.) and Dutch (Bakhuizen et al.) cohorts enrolled newly diagnosed patients and applied rigorous ACMG-based variant classification. What we meant was that we only included variants in leukemia associated genes while calculating the prevalence. We have removed this part and we no longer suggest that our lower prevalence is attributable to more stringent criteria.

We believe this revised, granular comparison demonstrates that our 1.7-3.3% prevalence is consistent with published literature when equivalent CPS definitions and leukemia-specific denominators are applied. Accordingly, we revised the second paragraph of the discussion between lines 336-349 (page 17).

13. In the Discussion, the authors state that "a phenotype-first approach based on ChiCaP criteria combined with reflex testing based on somatic findings would have identified all patients with pathogenic variants in leukemia predisposition genes." What did standard germline genetic diagnostics in children with leukemia look like prior to this study? Was this phenotype-driven? And was such testing no longer performed once patients were enrolled in this study? For example, was targeted TP53 germline testing no longer carried out in a patient with low-hypodiploid ALL?

Response: Please see our response to the question 10.

No systematic germline testing was conducted within the clinical practice prior to this study and targeted germline testing has relied on the initiative of the clinicians or diagnostic laboratory to recommend follow-up analyses due to phenotype or somatic findings triggering genetic testing. Although we lack data on the extent of germline testing performed in Sweden in children with leukemia before the study, it is reasonable to expect that low hypodiploidy in BCP-ALL would trigger germline testing for *TP53*.

We have added a clarification in the methods section (lines 655-657, page 30).

Minor comments:

• At what point were patients approached for participation in this study (i.e. the time interval between leukemia diagnosis and informed consent)?

Response: We thank the reviewer for this question. Families were approached for participation at the time of leukemia diagnosis. We recognize that this was not stated explicitly in the original manuscript and appreciate the opportunity to clarify. We have now

revised the Methods section under "Study Design, Patients, and Workflow" (lines 480-481, page 23).

• *The authors mention as a limitation of their study the "failure to detect mosaic variants present at levels below 20%" (lines 409–410). However, we do not find any description in the Methods section of a variant allele frequency (VAF)-based filter. Were variants with a VAF <20% directly filtered out? If so, please specify this explicitly in the Methods section.*

Response: We thank the reviewer for this observation. We acknowledge that the original Methods section lacked sufficient detail regarding variant allele frequency filtering and the practical sensitivity limits of our variant calling approach.

We have revised the Methods section to explicitly describe the variant callers used at each node, the relevant filtering parameters, and the rationale for the stated sensitivity limitation to detect mosaicism (lines 531-547, pages 25-26).

• *Line 417: What do the authors mean by "patients meeting high-risk clinical criteria"? Please specify which criteria are being referred to.*

Response: We agree that the phrase "patients meeting high-risk clinical criteria" was vague and potentially confusing in the context of our conclusion. We have revised the entire conclusion paragraph in line with other comments from the reviewers and explained high-risk clinical criteria under "Study Design, Patients, and Workflow" section (lines 506-508, page 24).

Reviewer #2 (Remarks to the Author):

I co-reviewed this manuscript with one of the reviewers who provided the listed reports. This is part of the Nature Communications initiative to facilitate training in peer review and to provide appropriate recognition for Early Career Researchers who co-review manuscripts.

Thank you for taking your time and reviewing our manuscript. Please see our answers to your colleague above.

Reviewer #3 (Remarks to the Author):

This manuscript describes the clinical yield of universal predisposition screening on childhood acute leukemia patients. Given the generally accepted rate of ~5-15% predisposing-allele associated cancers, it would be helpful to know if applying universal sequencing would be clinically appropriate in a "real world" unselected population. The study found a low prevalence of predisposing alleles (~5%); however, most (6 of 9) would be missed without this screening. Most of the mutation positive (for known leukemia

genes) cases had met clinical criteria based on ChiCaP criteria, but the three with solid tumor mutations did not. The authors argue against universal screening for mutations but referral to screening if ChiCaP criteria eg., family history and/or other defined abnormalities) are met.

Comment: We thank the reviewer for this clear and accurate summary of our study and for highlighting its key findings. We particularly appreciate the recognition that (i) a substantial proportion of clinically relevant variants (6/9) were unknown at diagnosis and (ii) patients with variants in solid tumor predisposition genes would not have been identified using existing clinical criteria alone. However, we do not argue against universal screening. We apologize that our original conclusions and recommendations were insufficiently clear, and we have carefully revised them throughout the manuscript.

In well-resourced settings such as Sweden, where all children with acute leukemia already undergo tumor-normal WGS analysis at diagnosis as part of standard clinical practice, we believe continuing three-pronged approach to detect ChiCaP is beneficial. In settings with limited resources, a phenotype-guided selection strategy appears to be a reasonable alternative. However, some cases might escape detection, such as patients with i) *de novo* variants, ii) variants in low-penetrant genes, iii) no phenotypic features, and iv) no material for somatic analyses. We emphasize that the choice of testing strategy should reflect local policies and resources, including the capacity for pre-test genetic counselling, as well as the decision of whether to restrict analysis to genes directly relevant to the child's cancer or to extend it to a broader set encompassing adult-onset disease.

Major:

1. Lines 319-325; 334-335; 359-361: Given the relatively low prevalence of pathogenic variants identified using the ChiCaP framework, please discuss whether incorporating additional cancer predisposition gene lists or datasets (beyond the 189 ChiCaP genes) could complement the current analysis. In particular, comment on which alternative or expanded gene sets might be appropriate and how their inclusion could potentially influence variant yield and interpretation. The investigators settled on the pre-designed 189 gene set but a post-hoc analysis could be fruitful (and the data is available).

Response: We thank the reviewer for this insightful and constructive suggestion. We agree that the relatively low prevalence of pathogenic germline variants identified using the ChiCaP 189-gene list (3.3%; 6/181) raises the important question of whether additional predisposition genes, not yet included in the ChiCaP gene panel, may contribute to leukemogenesis in this cohort.

Our aim was to evaluate the diagnostic yield and clinical utility of a uniformly applied three-pronged diagnostic strategy across centers, performed at diagnosis, including systematic phenotyping, germline WGS-based *in silico* 189-gene panel encompassing clinically actionable ChiCaP genes combined with tumor sequencing of positive cases across an unselected, population-based cohort of all children diagnosed with acute leukemia in Sweden.

The ChiCaP gene list was selected as the primary analysis strategy because it has been built upon well-established cancer predisposition genes and is suitable for use in clinical settings. Expanding the gene list within this particular manuscript would shift the scope from the evaluation of a germline testing strategy feasible within clinical diagnostics to a discovery-oriented analysis, which we believe warrants a dedicated investigation including parental samples, multiomics studies, etc.

We have clarified the aim of the study in the abstract and the introduction (lines 143-148, page 9).

Minor:
2. Lines 160-161: The manuscript states that 176 samples were derived from cultured skin fibroblasts and five samples from remission blood or bone marrow. Please clarify whether any patients identified as having ChiCaP syndromes were among these five cases. If so, discuss whether the use of remission blood or bone marrow as a germline DNA source could introduce bias and why or why not.

Response: Germline DNA derived from remission blood/bone marrow rather than fibroblasts carries a risk of somatic-to-germline misclassification; however, in none of these six patients (SE_AL010, SE_AL029, SE_AL039, SE_AL105, SE_AL109, SE_AL171), no P/LP germline ChiCaP variants were identified in the 189 analyzed genes. The patient (SE_AL029) had a known Down syndrome with trisomy 21 mosaicism which was below our detection rate in the remission bone marrow. Please also see our response to Reviewer 1 on this specific case. We added information on this in the discussion (lines 451-458, page 22) as well as in the methods section (lines 573-576, page 27).

3.Table 1:
a. The use of "N" for the characteristic "ChiCaP criteria available" (rows 10–16, including the header) is potentially misleading, as it may be misinterpreted at first glance to indicate that the ChiCaP criteria available for 181 patients. Please revise the labeling/table to improve clarity.

Response: Thank you for these thoughtful suggestions.

a. We appreciate you pointing out that the use of "N" for the "ChiCaP criteria available" row could be misleading. To prevent any misunderstanding, we have removed this row from the table entirely, as this information is already presented in the text (lines 165–166, page 10): "Complete ChiCaP criteria forms were available for 174 of the 181 enrolled patients (96%)."

b. Add a citation for the ChiCaP criteria

Response: b. Thank you for this suggestion. A citation for the ChiCaP criteria has been included in the manuscript (lines 153–165, page 10), where we briefly describe the criteria and reference our previous publication: "At study enrollment, we collected family history of cancer, malignancy type, and clinical features suggestive of ChiCaP using the structured ChiCaP criteria form described in detail by Tesi et al. (2024)[13]."

c. As family history and detailed ChiCaP criteria are described later in the Results section and in the Supplementary Materials, please expand the table legend to briefly define how family history was assessed and include explicit references to the relevant supplementary tables or sections where the full criteria (including family history and additional features) are described.

Response: c. We thank the reviewer for this suggestion. Based on your suggestion, we have expanded the legend of Table 1 to briefly define how family history was assessed and to include the full ChiCaP criteria. ChiCaP criteria form described in detail by Tesi et al. (2024).

4. Lines 166-167: "The manuscript states that "trio WGS - sequencing of the proband and both unaffected parents- was performed in 11 cases." Please clarify whether all parents in these 11 trios were indeed free of any cancer diagnosis. Additionally, clarify whether the term "proband" refers exclusively to the affected child or could also include a parent with a cancer diagnosis.

Response: We thank the reviewer for this important clarification request. We acknowledge that the original wording was imprecise and could be misleading.

The term "proband" in our study refers exclusively to the child who received the leukemia diagnosis. Regarding the parents included in the trio analyses, at the time of WGS analysis, both parents in all 11 trios were clinically healthy and free of active malignancy. However, family cancer history varied across trios (Supplementary Table 2): 3 of 11 families had no family history of cancer, while 5 of 11 had at least one parent with a prior cancer diagnosis before the age of 50.

To avoid ambiguity, we have revised the term "unaffected parents" to "parents" and added further clarification in the manuscript (lines 174-176, page 10). Trio analysis has been performed with all possible inheritance patterns including autosomal dominant.

5. Lines 237-242; 347-349: Please indicate whether there are previous publications reporting associations between the mutated genes identified in this study and specific leukemia subtypes. If exists, expand the discussion to place the current findings in the context of prior reports.

Response: We thank the reviewer for this insightful suggestion. We have conducted a thorough literature review to contextualize our findings regarding the three solid tumor predisposition genes - *MSH6*, *SDHA*, and *PALB2* - identified in our cohort and we have expanded the discussion with the following text (lines 370-380, pages 18-19):

"A review of the existing literature reveals no confirmed leukemia-associated monoallelic germline variants in these three genes. Hematological malignancy associated with *MSH6* and *PALB2* has predominantly been described in the context of biallelic inactivation[14, 15], whereas evidence for childhood leukemia in monoallelic carriers is limited to isolated case reports[11, 16, 17] and a comprehensive review of germline *SDHA* variants across cancer types found no cases of hematological malignancy among affected individuals[18, 19]. Taken together with the absence of second hits in the leukemias of these patients, the lack of established evidence linking heterozygous variants in these genes to childhood BCP-ALL support the interpretation that these findings are likely unrelated to leukemia development[20]. Larger cohorts and functional studies are required to establish whether leukemia falls within the phenotypic spectrum associated with these solid tumor predisposition genes."

6. Lines 263-265: As the ChiCaP criteria constitute a central methodological component of this study, the description of the use of "the structured ChiCaP criteria form described in detail by Tesi et al. (2024)" should be introduced earlier in the results section, rather than appearing only at the last part of the results.

Response: We thank the reviewer for this suggestion. We agree that the ChiCaP criteria form is a central methodological component and should be introduced earlier. Based on your suggestion in question 3c, we have expanded the legend of Table 1 to briefly define the ChiCaP criteria. The ChiCaP criteria is described in detail in the methods section under the title of "Study Design, Patients, and Workflow" (lines 489-508, pages 23-24).

7. The 181 patients were lower than expected for the ~28+ months of recruitment – what reason were the 32 patients missed? (ie., did they get treatment outside of Sweden, or

refused consent, or other reasons?). There is some confusion about diagnosis date and referral to the laboratory dates – is this the reason? (paragraph after line 558)

Response: We thank the reviewer for raising this important point, which was also addressed in response to Reviewer 1. We provide a summary here.

We updated the SCCR data. We have now included absolute numbers comparing enrollment across centers, leukemia subtypes, and age groups.

See the tables below.

Table 1: Based on participating center

**Center**	**ChiCaP study**	**SCCR data**	**Difference**
**Gothenburg**	62	60	-2
**Lund**	30	36	6
**Uppsala**	27	44	17
**Stockholm**	27	32	5
**Linköping**	18	18	0
**Umeå**	17	23	6
**Total**	181	213	**32**

Table 2: Based on leukemia subtype

	**ChiCaP study**	**SCCR data**	**Difference**
**ALL (B + T)**	156	182	26
**AML**	22	26	4
**Other**	3	2	-1
**Unspecified**	0	3	3
**Total**	181	213	**32**

Table 3: Based on age group

**Age**	**ChiCaP study**	**SBCR data**	**Difference**
**0-5 years**	103	127	24
**6-10 years**	44	46	2
**11-15 years**	24	31	7
**16-18 years**	10	9	-1
**Total**	181	213	**32**

All eligible patients were approached for participation at leukemia diagnosis. We do not think the differences between diagnosis date and referral to the laboratory dates can explain the missing 32 samples. The largest discrepancy by center was observed in Uppsala, where 27 patients were enrolled compared with 44 first leukemia diagnoses registered in the SCCR during the same period. To understand this gap, we contacted the Uppsala pediatric oncology team. Initially, an additional 3 patients had been recruited but were excluded from the study due to unavailable germline WGS data. The remaining discrepancy of approximately 14 cases is primarily attributed to challenges in obtaining written informed consent and limited research nurse availability during parts of the enrollment period in Uppsala.

For the other five centers, the differences between the study cohort and the SCCR ranged from 0 to 6 cases, indicating high enrollment rates.

Regarding leukemia subtypes, the ALL group shows a difference of 26 patients between the SCCR and GMS ChiCaP. The largest difference by age group was observed in the 0–5 years category (24 of 32 missing patients). This likely reflects, at least in part, the consent challenges described above, and that parents of very young children may be particularly overwhelmed during the early diagnostic period.

We acknowledge that we are unable to provide a detailed breakdown of specific reasons for declining study participation, as declination reasons were not systematically recorded during the consent process. This is a limitation that we have now noted in the discussion of the revised manuscript (lines 449-451, page 22).

We added the following sentence to the Methods section under the title of "Study Design, Patients, and Workflow" between lines 486-488 (page 23): "Not all eligible patients could be enrolled, primarily due to logistic challenges in obtaining written informed consent." We are not aware whether any patient received treatment abroad.

8. Unclear why the DMT3A mutation was not found in the individual case, but then found in the trio analysis? Actually this reviewer just figured out that DNMT3A was not part of the 189 gene panel. Which brings up another question - why not evaluate all patients with the OMIM list (without needing to do the trio).

Response: We thank the reviewer for this important question. The reviewer is correct that *DNMT3A* is not included in the 189-gene clinical ChiCaP panel used for singleton WGS analysis, which is why the pathogenic variant was only identified through clinical trio WGS. This gene was not an established leukemia predisposition gene at the time when we designed the gene panel.

The aim of our study was to evaluate the diagnostic yield and clinical utility of a uniformly applied three-pronged diagnostic strategy across centers, performed at diagnosis, including systematic phenotyping, germline WGS-based *in silico* 189-gene panel encompassing clinically actionable ChiCaP genes combined with tumor sequencing of positive cases across an unselected, population-based cohort of all children diagnosed with acute leukemia in Sweden. In addition, we performed trio-WGS with OMIM gene list for 11 high suspicion families.

Regarding the suggestion to evaluate all patients using the full OMIM gene list (~4,640 genes) without trio analysis: singleton short-read WGS with OMIM-wide filtering is technically feasible and would indeed increase gene coverage beyond current targeted panels. However, the interpretive challenges are substantial, including the increased risk of incidental findings. Without parental data, it is not possible to determine whether a rare variant is *de novo* or not. Furthermore, when two variants are identified in the same gene, singleton analysis cannot resolve whether they reside on the same allele (in cis) or on opposite alleles (in trans), which is critical for establishing biallelic pathogenicity in autosomal recessive conditions. Combined with the large volume of variants of uncertain significance generated across ~4,640 genes, these limitations considerably reduce clinical actionability. Trio analysis addresses each of these challenges by enabling *de novo* identification, phase resolution, and segregation analysis, thereby substantially reducing the interpretive burden. We therefore advocate for trio or family-based analysis where possible, particularly when novel or unexpected findings arise.

We believe future research approaches should integrate long-read whole-genome sequencing, comprehensive gene panels encompassing all OMIM disease genes, and deep exome sequencing to identify mosaicism, ideally anchored in both detailed phenotyping and somatic aberration analysis to guide germline interpretation. We have revised our concluding paragraph to make this point more explicit.

REFERENCES

1. Byrjalsen A*, et al.* Nationwide germline whole genome sequencing of 198 consecutive pediatric cancer patients reveals a high incidence of cancer prone syndromes. *PLoS Genet* **16**, e1009231 (2020).

2. Bakhuizen JJ*, et al.* Comparison of clinical selection-based genetic testing with phenotype-agnostic extensive germline sequencing to diagnose genetic predisposition in children with cancer: a prospective diagnostic study. *Lancet Child Adolesc Health* **8**, 751–761 (2024).

3. Lundin C*, et al*. High frequency of BTG1 deletions in acute lymphoblastic leukemia in children with down syndrome. *Genes Chromosomes Cancer* **51**, 196–206 (2012).

4. Hanada I*, et al.* Gene alterations involving the CRLF2-JAK pathway and recurrent gene deletions in Down syndrome-associated acute lymphoblastic leukemia in Japan. *Genes Chromosomes Cancer* **53**, 902–910 (2014).

5. Waanders E*, et al*. The origin and nature of tightly clustered BTG1 deletions in precursor B-cell acute lymphoblastic leukemia support a model of multiclonal evolution. *PLoS Genet* **8**, e1002533 (2012).

6. Buitenkamp TD*, et al*. Outcome in children with Down's syndrome and acute lymphoblastic leukemia: role of IKZF1 deletions and CRLF2 aberrations. *Leukemia* **26**, 2204–2211 (2012).

7. Aldoss I, Douer D, Pullarkat V. Therapy-related acute lymphoblastic leukemia: Where do we stand with regards to its definition and characterization? *Blood Rev* **37**, 100584 (2019).

8. Hofer KD*, et al.* Mutational patterns in therapy-related acute lymphoblastic leukemia subgroups: one step closer to unveiling the genetic odyssey. *Leuk Lymphoma* **65**, 808–815 (2024).

9. Stranneheim H*, et al*. Integration of whole genome sequencing into a healthcare setting: high diagnostic rates across multiple clinical entities in 3219 rare disease patients. *Genome Med* **13**, 40 (2021).

10. Zhang J*, et al*. Germline Mutations in Predisposition Genes in Pediatric Cancer. *New England Journal of Medicine* **373**, 2336–2346 (2015).

11. Newman S*, et al.* Genomes for Kids: The Scope of Pathogenic Mutations in Pediatric Cancer Revealed by Comprehensive DNA and RNA Sequencing. *Cancer Discov* **11**, 3008–3027 (2021).

12. Bakhuizen JJ*, et al*. Assessment of Cancer Predisposition Syndromes in a National Cohort of Children With a Neoplasm. *JAMA Netw Open* **6**, e2254157 (2023).

13. Tesi B*, et al.* Diagnostic yield and clinical impact of germline sequencing in children with CNS and extracranial solid tumors-a nationwide, prospective Swedish study. *Lancet Reg Health Eur* **39**, 100881 (2024).

14. Ripperger T, Schlegelberger B. Acute lymphoblastic leukemia and lymphoma in the context of constitutional mismatch repair deficiency syndrome. *Eur J Med Genet* **59**, 133–142 (2016).

15. McReynolds LJ, Biswas K, Giri N, Sharan SK, Alter BP. Genotype-cancer association in patients with Fanconi anemia due to pathogenic variants in FANCD1 (BRCA2) or FANCN (PALB2). *Cancer Genet* **258-259**, 101–109 (2021).

16. Onder G*, et al.* Genetic heterogeneity in childhood leukemia/lymphoma: a Turkish cohort with strong predisposition. *Front Genet* **16**, 1624306 (2025).

17. McGee RB*, et al.* Pathogenic Variants in Adult-Onset Cancer Predisposition Genes in Pediatric Cancer: Prevalence and Impact on Tumor Molecular Features and Clinical Management. *Clin Cancer Res* **29**, 1243–1251 (2023).

18. Dubard Gault M*, et al.* Germline SDHA mutations in children and adults with cancer. *Cold Spring Harb Mol Case Stud* **4**, (2018).

19. Casey RT*, et al.* SDHA related tumorigenesis: a new case series and literature review for variant interpretation and pathogenicity. *Mol Genet Genomic Med* **5**, 237–250 (2017).

20. Weijers DD*, et al.* Molecular analysis of cancer genomes in children with Lynch syndrome: Exploring causal associations. *Int J Cancer* **154**, 1455–1463 (2024).

NCOMMS-25-103638B Diagnostic Yield of Cancer Predisposition in Childhood Acute Leukemia: A Nationwide Prospective Swedish Study

REVIEWER COMMENTS & RESPONSES TO THE COMMENTS

Reviewer #1 (Remarks to the Author):

We thank the authors for extensively addressing our comments. We believe that the revisions made have substantially improved the readability of the manuscript. The aim of the study is clearer and conclusions are better in line with the results. The validity of this three-pronged diagnostic strategy in clinical practice is still a matter of debate. In the Swedish context, where WGS is already implemented as standard clinical practice, this strategy makes perfect sense, but for many other locations it will not be cost-effective.

We thank the Reviewer for their positive assessment and for recognizing the improvements made to the manuscript. We agree that the cost-effectiveness of this strategy depends on the local context, and that its rationale is strongest in settings such as Sweden where WGS is already established as standard clinical practice.

We have a few additional points that the authors may take into consideration:
- The authors have explained, in response to our questions, that all (likely) pathogenic variants that have now been detected with WGS would, in theory, also have been detectable with WES. However, this information is still missing in the manuscript. In our view, it would be useful to briefly mention this in the discussion.

Response: We thank the Reviewer for this suggestion, and we fully agree that this point deserves explicit mention in the Discussion. This clarification was incorporated into the revised manuscript (lines 429–431):

"Although the pathogenic variants identified in this study were confined to exonic regions and would have been detectable by exome sequencing or targeted panels, WGS enables detection of variant classes beyond the reach of exome-based approaches."

We apologize for not having explicitly drawn attention to this addition in our previous rebuttal letter, which understandably made it difficult to locate. We are grateful to the Reviewer for their careful reading and hope that the revised text addresses this point satisfactorily.

In addition, the methods section still lacks further explanation of how intronic variants were assessed. For example, was RNA-seq data used for intronic variants with suspected splice predictions? Precisely because this study uses WGS, this is relevant and useful information.

Response: We thank the Reviewer for this point, which is especially relevant given the genome wide nature of our data, and we have revised the Methods to make our variant-assessment strategy explicit.

At each participating center, sequencing data were analyzed following routine clinical diagnostic practice for rare germline variant detection, with interpretation focused on coding variants and variants in the canonical splice sites and adjacent splice regions of the 189-ChiCaP panel genes. Predicted splice effects were evaluated using *in silico* tools: 1. SpliceAI, added as a separate annotation within the analysis pipelines where available, and 2. the panel of splice-prediction algorithms implemented in Alamut Visual Plus, together with population allele frequencies from gnomAD. Variant review was extended to deeper intronic regions on a case-by-case basis, either where a specific clinical or genetic suspicion warranted closer inspection, or where intronic variants had previously been reported as (likely) pathogenic in ClinVar. Using this approach, no clinically significant deep intronic variants were identified in the genes of interest.

Regarding functional follow-up: where a strong intronic candidate variant of uncertain significance requires orthogonal validation of a predicted splicing or expression effect, we are able to perform RNA sequencing on fibroblast-derived RNA through a dedicated, publicly available national pipeline (Tomte; https://github.com/genomic-medicine-sweden/tomte), with Clinical Genomics Stockholm currently serving as the national center offering this service. In the present cohort, however, no intronic candidate variants emerged that warranted RNA-seq-based assessment of splicing or expression.

These clarifications have been added to the Methods (lines 556–563).

- Regarding our question related to the identity of the duplicated chr 21 in the different malignancies in the patient suspected of mosaic trisomy 21. We believe, however, that the statement: “establishing a shared constitutional origin” (page 15) is too strong because the chance that this has occurred coincidently cannot be ignored. Instead, “..is in line with..” may be a better formulation.

Response: We thank the Reviewer for this observation. We agree that "establishing a shared constitutional origin" is too strong, as an independent recurrent event cannot be excluded. We have revised the sentence accordingly (lines 234-236):

“Although the mosaicism was undetectable in peripheral blood and remission bone marrow, analysis of 24 informative SNPs across chromosome 21 confirmed duplication of the same chromosome 21 parental homolog in fibroblasts, AML, and BCP-ALL, which is in line with a shared constitutional origin, although an independent recurrent event involving the same homolog cannot be formally excluded.”

- On page 13 they refer to “…a TP53-related cancer predisposition syndrome…”. Although this wording may follow the rend to use gene-based references to syndromes, the authors may wish to consider whether Li–Fraumeni syndrome should be referred to explicitly by name in this section. Given its prominence as one of the best-recognized hereditary cancer predisposition syndromes, this would help to make the clinical context clearer for the reader.

Response: Thanks for this suggestion. We replaced TP53-related cancer predisposition syndrome with Li–Fraumeni syndrome at three occasions: lines 193, 264 and 347

Reviewer #2 (Remarks to the Author):
I co-reviewed this manuscript with one of the reviewers who provided the listed reports. This is part of the Nature Communications initiative to facilitate training in peer review and to provide appropriate recognition for Early Career Researchers who co-review manuscripts.

Thank you for taking your time and reviewing our manuscript. Please see our answers to your colleague above.

Reviewer #3 (Remarks to the Author):
The authors thoughtfully considered the comments and revised the manuscript with these in mind. This did not lead to any changes in the approach or the data presented, but will aid the reader in gaining an accurate assessment of the study purpose and yield. As the studies purpose did not include "discovery" of potential new relevant variants outside of the gene panel selected, such discovery potential is unrealized; but certainly falls out of the intended scope.

We thank the Reviewer for their careful and constructive engagement with our manuscript throughout the review process. We are pleased that the revisions have helped clarify the study's purpose and yield for the reader.
